# Innate immune responsiveness predicts enhanced cellular immunity and symptomatic disease after controlled human influenza infection

**DOI:** 10.1038/s41591-026-04483-7

**Published:** 2026-07-01

**Authors:** Loukas Papargyris, Jiayun Xu, Claire Broderick, Ao Huang, Trupti Gore, Arnold Reynaldi, Pete Dayananda, Ashley M. Collins, Stephanie Ascough, Nathan Wong, Jon Guy, Jihye Song, Satwik Kar, Emma Bergstrom, Lydia Slater, Zoe Gardener, Suzanna Paterson, Mahdi Moradi Marjaneh, Samuel J. Nichols, Victoria J. Wright, Min Kyu Park, Richard McKendry, Brad Nicholson, Micah McClain, Thomas W. Burke, Helen Wagstaffe, Jelle Klein, Michael Levin, Alba Grifoni, Christopher W. Woods, Miles P. Davenport, John S. Tsang, Benny Chain, Myrsini Kaforou, Christopher Chiu

**Affiliations:** 1 Department of Infectious Disease, Imperial College London, London, UK; 2 Center for Systems and Engineering Immunology (CSEI) and Department of Immunobiology, Yale University, New Haven, CT USA; 3 Program in Computational Biology and Biomedical Informatics, Yale University, New Haven, CT USA; 4 Division of Infection and Immunity, University College London, London, UK; 5 Kirby Institute, University of New South Wales, Kensington, New South Wales Australia; 6 Department of Medicine, Duke University School of Medicine, Durham, NC USA; 7 SGS Clinical Pharmacology Unit, Edegem, Belgium; 8 Center for Vaccine Innovation, La Jolla Institute for Immunology (LJI), La Jolla, CA USA; 9 Department of Biomedical Engineering, Yale University, New Haven, CT USA; 10 Department of Computer Science, University College London, London, UK

**Keywords:** Infection, Innate immunity, Influenza virus

## Abstract

Controlled human influenza infection studies can uniquely interrogate the early immune factors associated with clinical outcome. In this study, 27 healthy volunteers with low strain-specific serum neutralizing antibody levels were challenged with influenza A/H3N2 virus. Twenty-two became infected, with 18 developing mild-to-moderate symptoms and four remaining asymptomatic. Local and systemic immune profiling revealed innate pathways that engaged more rapidly and to a higher level in symptomatic participants. Earlier monocyte and dendritic cell activation correlated with higher symptom scores but also enhanced natural killer and CD8^+^ T cell activation thereafter. At baseline, peripheral blood mononuclear cells from symptomatic participants were more responsive to in vitro challenge, indicating a predisposition to divergent immunological outcomes at the time of virus exposure that was subsequently modulated by infection. These results show that human innate cell responsiveness is a predeterminant of both symptomatic disease and cellular immune responses known to promote viral clearance, suggesting potential targets for therapeutic intervention if decoupled.

## Abstract (web summary)

Local and systemic immune profiling after a controlled human influenza infection challenge in humans found that the participants with increased innate cell responsiveness were more likely to become symptomatically infected.

## Main

Disease severity after influenza infection depends on a complex interplay among virus, host and environmental factors that remains incompletely understood. Although newly emergent viruses can be highly pathogenic, especially in immunologically naive hosts, seasonal strains are also responsible for substantial morbidity in some people^1–3^. Observational studies suggest that severe disease is associated with excessive inflammatory responses, including hypercytokinemia, but why some develop this and not others when infected with the same virus is unknown^4–6^. Conversely, pro-inflammatory immune responses are also positively associated with enhanced adaptive immunity and are likely necessary for generation of robust protection.

Well-recognized patient-related susceptibility factors include extremes of age, comorbidities, obesity, socioeconomic background, pregnancy and genetic predispositions^7–10^. Furthermore, virus-specific antibodies and T cells are important in protection^11–13^, although they do not fully explain the interindividual differences seen after virus exposure. Innate immune responses may have an impact on protection and disease either in concert with adaptive immunity or independently^6,14–17^. However, with such responses appearing so early after virus exposure, most translational studies have been unable to examine these in depth.

Controlled human infection (CHI) studies provide unique opportunities to closely monitor symptom development and immune responses, permitting the investigation of early immune kinetics in the context of mild-to-moderate self-limiting disease^18,19^. In the present study, we longitudinally analyzed the immune profiles of 27 healthy adult volunteers inoculated with influenza A/H3N2. We show that early innate responses correlate with both higher symptom scores and subsequent cellular immune response, revealing a pattern of quicker and greater immune activation to which individuals who proceed to symptomatic disease are predisposed at the point of virus exposure.

## Results

### Viral load fails to explain asymptomatic infection in influenza A/H3N2-inoculated volunteers

**Fig. 1:**
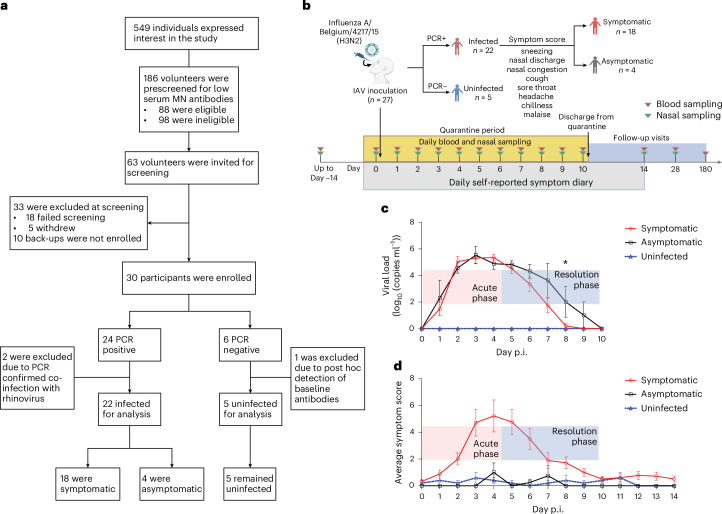
Study setup and clinical outcomes. Healthy adult volunteers were enrolled, assessed and sampled periodically before and after inoculation with a GMP-manufactured influenza A(H3N2) virus. **a**, CONSORT diagram showing participant enrollment, infection outcomes and development of symptomatic disease. **b**, Diagram showing the study setup and sampling timepoints. Diagram created in BioRender; Xu, J. https://biorender.com/xtooyrz (2026). **c**, Average viral shedding of symptomatic (*n* = 22), asymptomatic (*n* = 4) and uninfected (*n* = 5) participants as determined by M gene qPCR from nasal lavage. Significance between symptomatic and asymptomatic participants for each timepoint was tested by two-sided Mann−Whitney *U*-tests (day 8 p.i. *P* = 0.026, *P < 0.05). **d**, Self-reported total daily symptom score for symptomatic (*n* = 22), asymptomatic (*n* = 4) and uninfected (*n* = 5) participants. Data in **c**,**d** show mean ± s.e.m. MN, microneutralization assay; IAV, influenza A virus.

Thirty healthy participants aged 18−55 years, with low strain-specific serum neutralizing antibody titers (≤1:20 by microneutralization), were inoculated with 5 × 10^5^ 50% tissue culture infectious dose (TCID_50_) of influenza A/Belgium/4217/2015 intranasally (Fig. 1a,b). Of these, three were excluded due to viral co-infection or seroconversion before inoculation. After inoculation, participants were quarantined for up to 10 days with daily sampling and follow-up at days 14, 28 and 180 (Fig. 1b). Symptoms were monitored using self-reported symptom diaries. Of the 27 participants, 22 (81.5%) developed polymerase chain reaction (PCR)-confirmed infection and five (18.5%) remained uninfected. Infected participants were categorized using the modified Jackson criteria^20^ into symptomatic (18/22; 81.8%) and asymptomatic (4/22; 18.2%) groups. No significant differences were observed in demographics between groups and no differences related to sex or gender; male and female participants were, therefore, analyzed together (Extended Data Table 1).

In symptomatic participants, viral load (VL) and symptom score both peaked at day 4 post-inoculation (p.i.), thus defining an acute phase from day 0 to day 4 and a resolution phase from day 5 onwards (Fig. 1c,d). In asymptomatically infected participants, VL peaked at day 3, but symptoms remained essentially zero throughout. Uninfected participants did not develop any detectable VL or symptoms (Fig. 1c,d and Supplementary Fig. 1). Symptomatic participants showed a trend toward more rapid viral clearance, with significantly lower VL on day 8 p.i. (Mann−Whitney, *P* = 0.026) although not statistically significant by linear mixed-effects modeling. However, no differences were observed between the VLs of symptomatic participants and asymptomatic participants during the acute phase.

### Symptomatic infection is associated with early innate transcriptional changes

**Extended Data Fig. 1:**
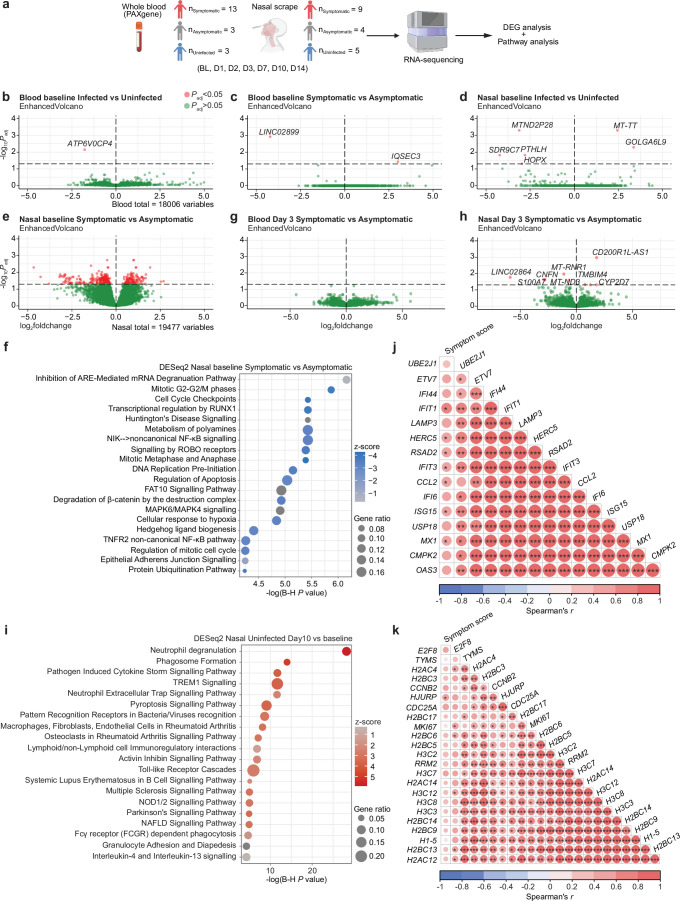
Comparison of DEGs between groups in the blood and the nasal mucosa at baseline and day 3 p.i. **a**, Whole blood and nasal mucosal samples were analysed by RNA sequencing. Created in BioRender; Xu, J. https://biorender.com/767ge4v (2026). **b-e**, Unpaired comparison between groups was performed for differential expression analysis using DESeq2. Volcano plots represent DEGs (*P*_adj_ < 0.05) between infected and uninfected (**b**) and symptomatic and asymptomatic (**c**) participants in the blood, and infected and uninfected (**d**) and symptomatic and asymptomatic (**e**) participants in the nasal mucosa at baseline (day 0 and day -14 pre-inoculation for blood and nasal mucosa, respectively). **f**, Differential IPA pathway enrichment in the nasal mucosa pre-inoculation between symptomatic and asymptomatic participants. **g,h**, Unpaired comparison between groups was performed for differential expression analysis using DESeq2. Volcano plots represent DEGs (*P*_adj_ < 0.05) between symptomatic and asymptomatic in the blood (**g**) and in the nasal mucosa (**h**) at day 3 p.i. **i**, Nasal DEG-enriched pathways of uninfected participants at day 10 post-inoculation compared to baseline. Top 20 significant IPA pathways enriched in DEGs in the nasal mucosa of uninfected participants at day 10 post-inoculation compared to baseline (Benjamini−Hochberg *P*_adj_ < 0.05). **j**, Spearman’s correlation between normalised gene counts (AUC from baseline to day 3) of DEGs from cluster 2 and modified Jackson symptom scores. **k**, Spearman’s correlation between normalised gene counts (day 7 p.i. fold change from baseline) of DEGs from cluster 5 and modified Jackson symptom scores. For **b-e,g,h**, significance between groups was tested by a two-sided Wald test (genes shown as red dots for *P*_adj_ < 0.05 or green dots for *P*_adj_ > 0.05). For **e**, the full list of the DEGs (*n* = 431) is shown in Supplementary Table 2. For j,k, significance was assessed using Spearman’s rank correlation coefficient (two-sided). *P < 0.05, **P < 0.01, ***P < 0.001. BL, baseline; D, day; B-H, Benjamini−Hochberg.

To reveal pathways potentially explaining these differential clinical outcomes, whole blood (systemic) and inferior turbinate tissue (nasal mucosa) samples were analyzed by RNA sequencing (Extended Data Fig. 1a). Differentially expressed gene (DEG) analysis by DESeq2 revealed minimal pre-inoculation differences in gene expression of blood associated with clinical outcome (Extended Data Fig. 1b,c and Supplementary Table 1). In nasal mucosa at baseline, only six DEGs were identified comparing infected and uninfected, whereas 431 DEGs appeared between symptomatic and asymptomatic (Extended Data Fig. 1d,e and Supplementary Tables 1 and 2). However, on detailed inspection, no interferons (IFNs), interferon-stimulated genes (ISGs) or cytokines/chemokines associated with viral infection were present among these baseline DEGs, and Ingenuity Pathway Analysis (IPA) indicated only one enriched pathway that was directly immune related: NIK-related non-canonical NF-κB signaling (Extended Data Fig. 1f). Further unpaired analysis comparing symptomatic and asymptomatic participants at peak VL/day 3 p.i. revealed no statistically significant differences in blood and only nine DEGs in the nasal mucosa (Extended Data Fig. 1g,h and Supplementary Table 1).

**Fig. 2:**
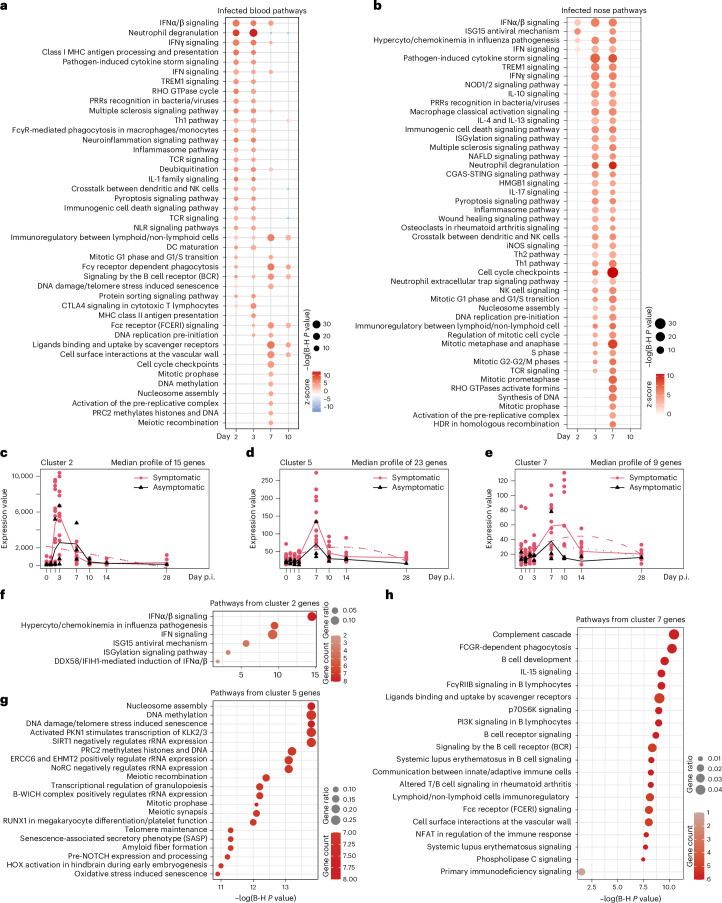
Symptomatic infection is associated with early local and systemic innate transcriptional changes that precede cellular activation signatures. **a**, The most significantly DEG-enriched pathways (Benjamini−Hochberg *P*_adj_ < 10^−5^, *z*-score > 3.5) in the blood p.i. are shown by IPA. **b**, The most significantly DEG-enriched pathways (Benjamini−Hochberg *P*_adj_ < 10^−6^, *z*-score > 3.5) in the nasal mucosa p.i. are shown by IPA. **c**−**e**, Gene expression clusters 2 (**c**, *n* = 15), 5 (**d**, *n* = 23) and 7 (**e**, *n* = 9) were identified by maSigPro. Solid lines connect the median DEG expression values for each group of participants, and dashed lines show the regression curves fitted to the data. **f**−**h**, DEG-enriched pathways (Benjamini−Hochberg *P*_adj_ < 0.05) with DEGs from clusters 2 (**f**), 5 (**g**, top 20 pathways out of 49) and 7 (**h**) are shown by IPA. For **a**,**b**,**f**−**h**, significance was assessed using right-tailed Fisherʼs exact tests. The *P* values were adjusted using the Benjamini−Hochberg method for multiple hypothesis test correction. For **b**, the most significantly DEG-enriched pathways using as cutoff Benjamini−Hochberg *P*_adj_ < 10^−5^ and *z*-score > 3.5 are shown in Supplementary Fig. 5. B-H, Benjamini−Hochberg.

Next, pairwise comparisons were undertaken to identify DEGs between each p.i. timepoint and pre-inoculation. In whole blood from uninfected participants, no significant DEGs were identified during the acute phase (Supplementary Fig. 2 and Supplementary Table 3). During convalescence at day 14, 38 DEGs were identified, but IPA showed no relevant functional enrichment (Supplementary Tables 4 and 5). By contrast, among infected participants, 4,030 DEGs were identified at day 2 and 6,560 at day 3 p.i. Hierarchical clustering of the top DEGs (*n* = 179, log_2_ fold change > 2 and adjusted *P* value (*P*_adj_) < 0.01) visualized by heatmap revealed two major clusters, with a group of 161 DEGs peaking at day 2 (46) or day 3 (115) p.i. and a smaller group of 18 DEGs peaking at day 7 p.i. (Supplementary Fig. 2; DEGs detailed in Supplementary Table 6). Using IPA, top DEG-enriched pathways at days 2−3 p.i. were mostly involved in pro-inflammatory and innate immune processes, with a pattern of quicker and greater induction in symptomatic participants (Fig. 2a and Supplementary Fig. 3). By contrast, at day 7 p.i., top pathways were primarily involved in cell cycle and DNA replication, indicative of cellular proliferation (Fig. 2a).

Surprisingly, transcriptional responses in nasal tissue were delayed compared to the circulation. After inoculation, 46 DEGs at day 2, 2,775 DEGs at day 3 and 3,496 DEGs at day 7 were found in the nasal mucosa. In uninfected participants, minimal differential gene expression was observed except at day 10 p.i. where 240 DEGs were seen (driven by a single outlying asymptomatic participant, resulting in enrichment of neutrophil-related pathways by IPA) (Extended Data Fig. 1i). After infection, hierarchical clustering of the top DEGs (*n* = 264) was visualized by heatmap (Supplementary Fig. 4; DEGs detailed in Supplementary Table 6). Pathway analysis of significant nasal DEGs using IPA revealed similar pathways as blood, but these were slower to resolve, persisting to at least day 7 p.i. (Fig. 2b).

### Cellular activation signatures are preceded by early antiviral transcriptional responses

To further investigate longitudinal patterns of transcriptomic change, a temporal model was generated using maSigPro, clustering significant DEGs based on their similarity in expression patterns over time and between groups. This generated nine clusters with distinct temporal patterns of expression (Supplementary Fig. 6; corresponding DEG-enriched pathways in Supplementary Fig. 7). Gene clusters generated from nasal tissue revealed no consistent patterns; further analysis, therefore, focused on blood. Three clusters (clusters 2, 5 and 7) demonstrated clear responses to infection (Fig. 2c–e), consistent with earlier pairwise comparisons. Cluster 2 was dominated by IFN signaling, with 15 DEGs grouped by IPA as primarily ISGs (Fig. 2f and Supplementary Table 7). Supporting this unsupervised analysis, a panel of canonical ISGs, including those in cluster 2, was selected for individual-gene longitudinal tracking, with both analyses again showing an early peak in symptomatic participants around day 3 and lower response for asymptomatic participants (Fig. 2c and Supplementary Fig. 8a). Cumulative expression values during the acute phase (area under the curve (AUC), baseline to day 3) of seven of 15 DEGs in cluster 2 correlated significantly with symptom scores (Extended Data Fig. 1j). By contrast, ISGs in the nasal mucosa showed trends toward higher peak expression in asymptomatic participants but with substantially greater variability, thus limiting interpretation (Supplementary Fig. 8b).

In cluster 5, 23 DEGs peaked at day 7 with higher expression levels in symptomatic participants (Fig. 2d). These enriched for cell cycle, DNA repair and epigenetic modification pathways (Fig. 2g). The day 7 fold change (from baseline) of normalized gene counts of 15 of 23 DEGs in cluster 5 correlated with symptom scores, implying a relationship between symptomatology and later enhancement of cellular responses (Extended Data Fig. 1k). Additionally, nine DEGs in cluster 7 peaked at day 7 for asymptomatic participants and at day 10 for symptomatic participants where gene expression was more prolonged and pronounced (Fig. 2e). Cluster 7 DEGs mostly enriched for complement cascade, Fcγ receptor-dependent phagocytosis and B cell development pathways (Fig. 2h). Thus, gene expression analyses imply that greater early innate activation correlates with both concurrent symptoms and subsequent cellular activation/proliferation.

### Soluble mediators are induced earlier and to higher levels in symptomatic participants

**Fig. 3:**
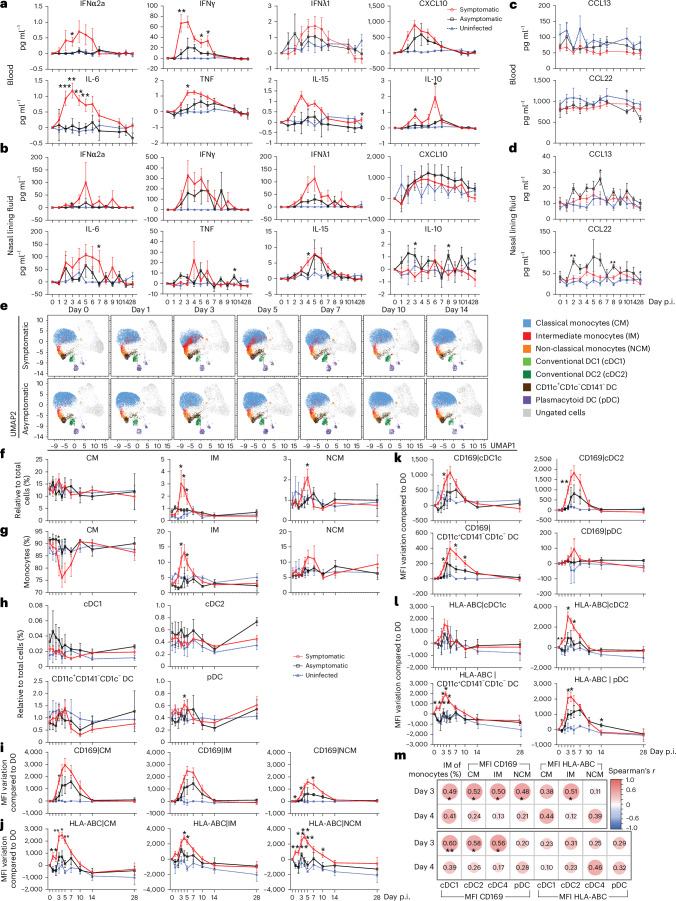
Early soluble mediators and monocyte/DC activation are associated with symptoms. **a**−**d**, Soluble mediators (**a**,**b**) and CCL13 and CCL22 (**b**,**d**) in plasma (**a**,**c**) and nasosorption (**b**,**d**) samples from symptomatic (*n* = 18), asymptomatic (*n* = 4) and uninfected (*n* = 5) participants were measured by MSD. Data are mean ± s.e.m. **e**, UMAP analysis was undertaken on gated live lineage^neg^ cells based on the markers CD14, CD16, CD11c, CD1c, CD141, CD123 and HLA-DR, for symptomatic and asymptomatic participants. Monocyte and DC subgroups were manually gated and overlayed onto the corresponding UMAP plots. **f**,**g**, Relative percentages of monocyte subgroups compared to total cells (**f**) or total monocytes (**g**) acquired by flow cytometry from symptomatic, asymptomatic and uninfected participants. **h**, Frequency of DC subgroups compared to total cells. **i**,**j**, Surface expression of CD169 (**i**) and HLA-ABC (**j**) on monocyte subgroups. **k**,**l**, Surface expression of CD169 (**k**) and HLA-ABC (**l**) on DC subgroups. **m**, Correlation matrix showing Spearmanʼs *r* numbers between the relative percentage of IMs (out of total monocytes), CD169 and HLA-ABC MFI at day 3 and day 4 p.i. in monocyte and DC subgroups and the modified Jackson symptom score for all available infected participants (day 3: *n* = 19 and day 4: *n* = 16). Significance was assessed using Spearmanʼs rank correlation coefficient (two-sided). For **a**−**d**,**f**−**l**, significance between symptomatic and asymptomatic participants was tested by a two-way linear mixed-effects model (REML) with Geisser−Greenhouse correction, and post hoc pairwise comparisons were adjusted using the Holm−Sidak method. Results are shown as mean ± s.e.m. No statistical tests are shown for uninfected participants. For **a**,**b**,**i**−**l**, data were normalized to baseline (day 0 values were abstracted from each timepoint). For **e**,**f**,**l**, data were generated from *n* = 18 symptomatic, *n* = 4 asymptomatic and *n* = 5 uninfected participants (*n* numbers per timepoint are detailed in the Methods). *P < 0.05, **P < 0.01, ***P < 0.001, ****P < 0.0001. MFI, median fluorescence intensity; D0, day 0; REML, restricted maximum likelihood.

**Extended Data Fig. 2:**
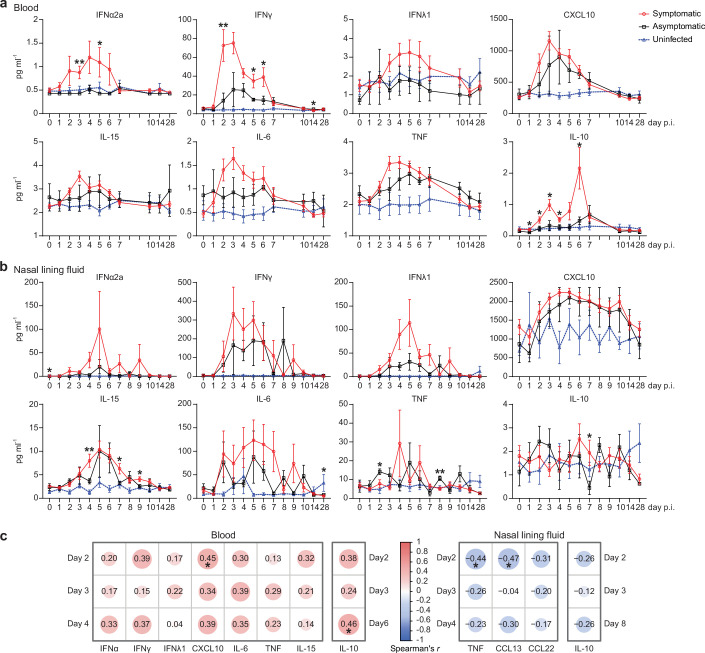
Soluble mediator concentrations in plasma and nasal lining fluid. **a-b**, Cytokine and chemokine levels from plasma samples (**a**) and nasosorption samples (**b**) from symptomatic (*n* = 18), asymptomatic (*n* = 4) and uninfected (*n* = 5) participants as measured by Meso Scale Discovery. Data are raw concentrations at pg/ml and results are shown as mean concentrations ± s.e.m., every day from day 0 (pre-inoculation) until day 7 and at days 10, 14 and 28 p.i. for plasma samples, or from day 0 (pre-inoculation) until day 10 and at days 14 and 28 p.i. for nasosorption samples. Undetectable sample values were given the average lower limit of detection (LLOD) among all plates. Significance between symptomatic and asymptomatic participants was tested by a two-way linear mixed-effects model (REML) with Geisser−Greenhouse correction and post hoc pairwise comparisons were adjusted using the Holm−Šidák method. No statistical tests are shown for uninfected participants. **c**, Correlation matrix showing Spearman’s r numbers between soluble mediator concentrations at different timepoints in the blood (left) and nasal lining fluid (right) and the modified Jackson symptom scores for all infected participants (n = 22). Significance was assessed using Spearman’s rank correlation coefficient (two-sided). *P < 0.05, **P < 0.01, ***P < 0.001, ****P < 0.0001. REML, restricted maximum likelihood.

With cytokine signaling pathways having dominated the transcriptomic response, soluble mediators in plasma and nasal lining fluid were next analyzed. At baseline, no significant differences were observed in the levels of major inflammatory mediators (IFNγ, IFNλ1/IL-29, CXCL10/IP-10, IL-6, TNF, IL-15), IL-10 or the chemokines CCL13/MCP-4 and CCL22/MDC (Extended Data Fig. 2a,b; other soluble mediators in Supplementary Figs. 9 and 10). After inoculation, no responses were seen in uninfected participants, but greater and quicker elevation in several soluble mediators was observed acutely in symptomatic participants, with significantly higher systemic IFNγ and IL-6 by day 2 and IFNα2a, TNF and IL-10 by day 3 p.i. and IL-15 showing a similar trend (Fig. 3a). Additionally, IL-10 and IFNγ showed biphasic responses with a second peak of significantly increased plasma concentrations at day 6 in symptomatic participants (Fig. 3a), aligned with and potentially reflecting the cluster of DEGs upregulated at day 7 (Fig. 2d).

Analysis of the aforementioned mediators in nasal mucosa confirmed a delayed inflammatory response, with IFNα2a, IFNγ, IL-6 and IL-15 peaking 1−2 days after blood. Here, only IL-6 and IL-15 intermittently showed significantly higher expression in symptomatic participants (Fig. 3b). By contrast, in a reversal of the pattern seen in blood, there were significantly higher levels of nasal IL-10 by day 3, of nasal CCL13 at day 6 and of nasal CCL22 at day 2 in the asymptomatic group (Fig. 3a–d). Significant negative correlations between day 2 nasal TNF and CCL13 concentrations with symptom scores were identified, suggesting potentially protective associations, whereas day 2 plasma CXCL10 and day 6 IL-10 concentrations were positively correlated with symptom scores, suggesting that both pro-inflammatory and anti-inflammatory mediators may be involved with symptom development (Extended Data Fig. 2c).

### Symptoms are associated with early myeloid activation and antigen cross-presenting capacity

**Extended Data Fig. 3:**
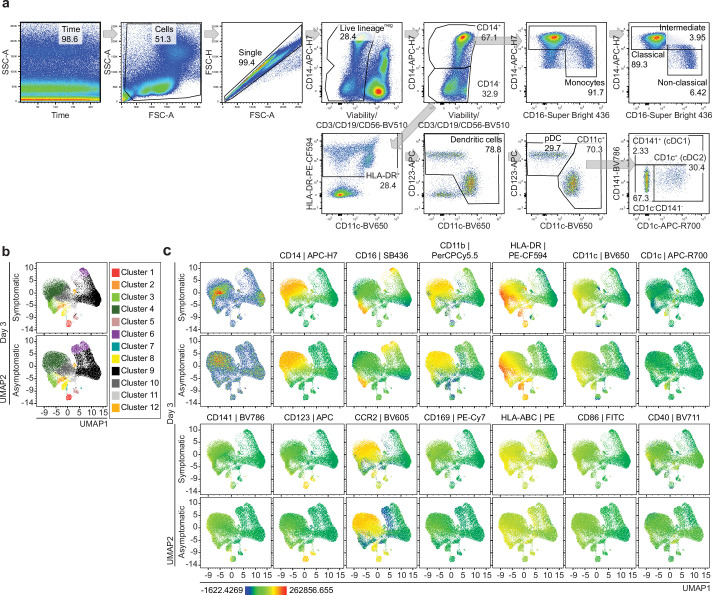
Flow cytometry gating strategy and extended dimensionality reduction analysis of monocytes and DCs. **a**, Gating strategy for PBMC subpopulation phenotyping. Live monocytes have been assigned as CD14^+/dim^ excluding other lineage positive and CD14^dim^CD16^−^ cells and have been separated into three subgroups: CD14^+^CD16^−^ classical monocytes (CMs), CD14^+^ CD16^+^ intermediate monocytes (IMs) and CD14^dim^CD16^+^ non-classical monocytes (NCMs). Live DCs have been assigned as CD14^−^HLA-DR^+^ after excluding other lineage positive cells. DCs were then separated into: CD11c^+^CD141^+^ conventional DC1s (cDC1s), CD11c^+^CD141^−^CD1c^+^ conventional DC2s (cDC2s), CD11c^+^CD141^−^CD1c^−^ DCs and CD123^+^ plasmacytoid DCs (pDCs). **b**, UMAP and FlowSOM analysis on gated live lineage^neg^ cells based on the markers CD14, CD16, CD11c, CD1c, CD141, CD123 and HLA-DR, for symptomatic and asymptomatic participants at day 3 p.i. **c**, Heatmap UMAP plots showing expression of innate cell markers at day 3 p.i. For **b,c**, numbers of samples analysed are detailed in the Methods.

**Extended Data Fig. 4:**
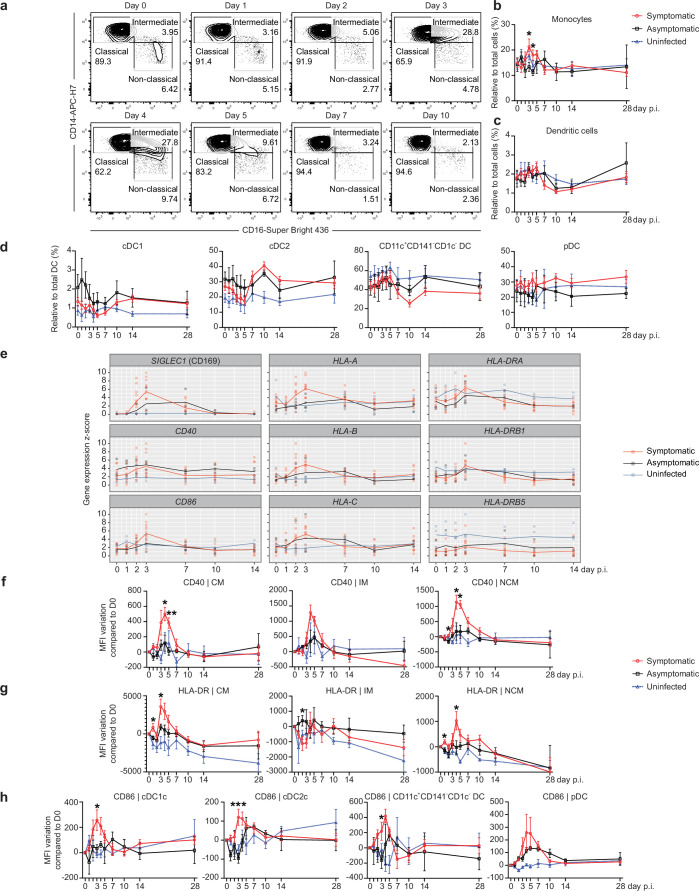
Extended phenotyping of monocytes and DCs and single gene expression levels in the blood. **a**, Plots showing the changing ratio of monocyte subgroups from day 0 to day 5 and at days 7 and 10 post-inoculation for a representative symptomatic participant. **b,c**, Monocyte (**b**) and DC (**c**) frequencies in PBMCs, from symptomatic, asymptomatic and uninfected participants, relative to total cells acquired by flow cytometry. **d**, Relative percentages of DC subgroups compared to total DCs. **e**, Normalized gene expression levels (standardised into z-scores) in the blood are longitudinally visualized for symptomatic (*n* = 13), asymptomatic (*n* = 3) and uninfected (*n* = 3) participants. **f-h**, Surface expression of CD40 (**f**) and HLA-DR (**g**) on monocyte subgroups and CD86 (**h**) on DC subgroups. Surface marker expression levels are shown as MFI variation compared to day 0 (day 0 MFI has been abstracted from each later timepoint). For **b-d,f-h**, data were generated from *n* = 18 symptomatic, *n* = 4 asymptomatic and *n* = 5 uninfected participants (*n* numbers per timepoint are detailed in the Methods). Data are mean ± s.e.m., every day from day 0 until day 5 and at days 7, 10, 14 and 28 p.i. Significance between symptomatic and asymptomatic participants was tested by a two-way linear mixed-effects model (REML) with Geisser−Greenhouse correction and post hoc pairwise comparisons were adjusted using the Holm−Šidák method. No statistical tests are shown for uninfected participants. *P < 0.05, **P < 0.01, ***P < 0.001. MFI, median fluorescence intensity; D0, day 0; REML, restricted maximum likelihood.

With multiple myeloid and antigen presentation pathways enriched for DEGs, we proceeded to investigate innate cells by flow cytometry (Extended Data Fig. 3a and Supplementary Table 8). Monocytes are divided into three subgroups: CD14^+^CD16^−^ classical monocytes (CMs), CD14^+^CD16^+^ intermediate monocytes (IMs) and CD14^dim^CD16^+^ non-classical monocytes (NCMs). Dendritic cells (DCs) are defined as CD14^−^HLA-DR^+^ and divided into four subgroups: CD123^+^ plasmacytoid DC (pDC), CD11c^+^CD141^+^ conventional DC1 (cDC1), CD11c^+^CD141^−^CD1c^+^ conventional DC2 (cDC2) and a CD11c^+^CD141^−^CD1c^−^ less-characterized DC subgroup^21^. To visualize changes, dimensionality reduction by uniform manifold approximation and projection (UMAP) gated on live, lineage^neg^ (CD3^−^CD19^−^CD56^−^) cells was performed. Unsupervised clustering by FlowSOM did not distinguish between monocyte subgroups (day 3 p.i.; Extended Data Fig. 3b,c), but manual gating showed CMs as the dominant population in blood throughout infection, with expansion of IMs and NCMs occurring during the acute phase, suggesting that these might have emerged from the CM pool (Fig. 3e and Extended Data Fig. 4a).

In symptomatic participants only, a significant increase in total circulating monocytes was seen peaking at day 3 and persisting until day 5 (Extended Data Fig. 4b), but no significant differences were observed in total DCs (Extended Data Fig. 4c). Although all monocyte subgroups increased as a percentage of total cells, this was greatest in IMs followed by NCMs that peaked 2 days later at day 5 (Fig. 3f). Similar differences were seen by percentage of total monocytes (Fig. 3g). The DC response to infection was predominantly marked by a significantly greater frequency of pDCs in symptomatic participants that peaked at day 4 p.i. (Fig. 3h and Extended Data Fig. 4d).

**Extended Data Fig. 5:**
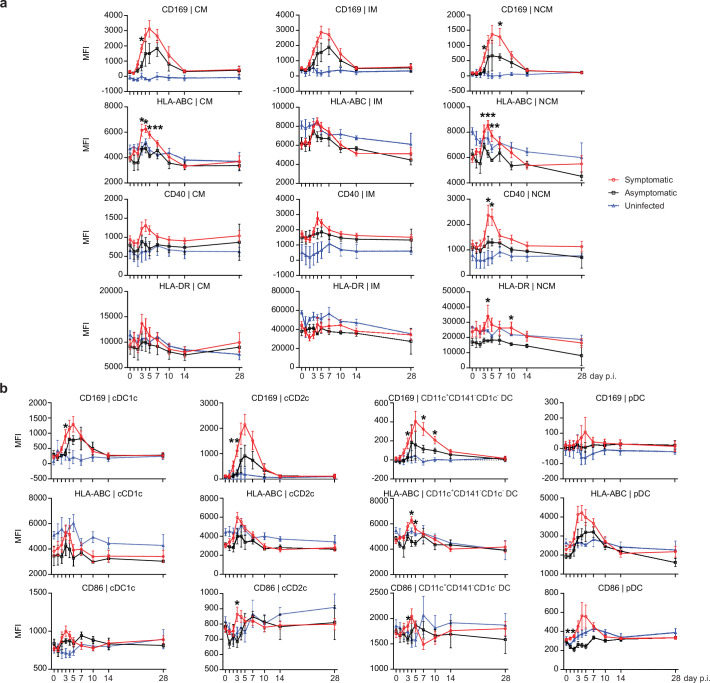
Extended data showing expression of surface markers by flow cytometry. **a**, Surface expression of CD169, HLA-ABC, CD40 and HLA-DR on monocyte subgroups and **b**, surface expression of CD169, HLA-ABC, CD86 on DC subgroups. Data were generated from *n* = 18 symptomatic, *n* = 4 asymptomatic and *n* = 5 uninfected participants (*n* numbers per timepoint are detailed in the Methods). Expression levels are shown as MFI. Data are mean ± s.e.m., every day from day 0 until day 5 and at days 7, 10, 14 and 28 p.i. Significance between symptomatic and asymptomatic participants was tested by a two-way linear mixed-effects model (REML) with Geisser−Greenhouse correction and post hoc pairwise comparisons were adjusted using the Holm−Šidák method. No statistical tests are shown for uninfected participants. *P < 0.05, **P < 0.01, ***P < 0.001. MFI, median fluorescence intensity; REML, restricted maximum likelihood.

Comparison of DEG kinetics had highlighted the more rapid upregulation of genes such as *SIGLEC1* (CD169), *HLA-A*, *HLA-B*, *HLA-C* (HLA-ABC), *CD86*, *CD40* and *HLA-DRA, HLA-DRB1, HLA-DRB5* (HLA-DR) in symptomatic participants (Extended Data Fig. 4e). Here again by flow cytometry, the pattern of quicker and greater increase in expression associated with symptomatic infection was shown for CD169, HLA-ABC and CD40 within 4 days p.i. on all three monocyte subgroups and CD169, HLA-ABC and CD86 on all four DC subgroups (Fig. 3i–l and Extended Data Fig. 4f,h (normalized) and Extended Data Fig. 5 (non-normalized)). Expression of HLA-DR on monocytes was similarly consistent with the exception of IMs, where expression in symptomatic individuals showed a short-lived decrease with a trough at day 2 that was significantly lower than asymptomatic participants, which may be due to transition of CMs, with their lower baseline expression of HLA-DR, to IMs (Extended Data Fig. 4g). Significant correlation between the frequency of IMs or MFI of CD169 and HLA-ABC and symptoms was observed at days 3−4 p.i. in all infected participants (Fig. 3m). Thus, symptom scores significantly correlated with day 3 IM percentage, with day 3 CD169 MFI on all monocyte and DC subgroups except pDCs and with day 3 HLA-ABC MFI on IMs.

Therefore, earlier and greater activation of circulating monocytes and DCs alongside increased expression of markers involved in antigen presentation/cross-presentation distinguished symptomatic from asymptomatic influenza infection, with frequency of IMs and expression level of CD169 on monocytes and cDCs potentially representing symptomatic correlates.

### Natural killer and CD8^+^ T cell proliferation correlates with early systemic innate cell activation

**Fig. 4:**
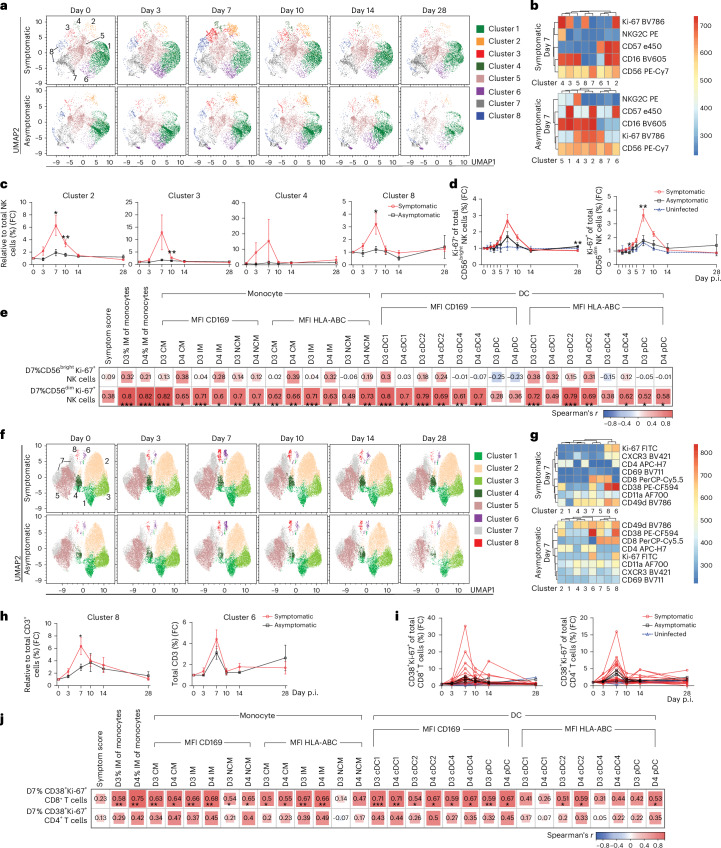
NK and CD8^+^ T cell activation and proliferation correlate with systemic innate cell activation. **a**, UMAP analysis and FlowSOM clustering were undertaken on gated NK cells based on the markers CD56, CD16, CD57, NKG2C and Ki-67. **b**, Heatmaps of marker expression by FlowSOM NK cell clusters for symptomatic (top) and asymptomatic (bottom) participants at day 7. **c**, Longitudinal analysis of NK cell FlowSOM cluster percentage fold change out of total NK cells. **d**, Percentage fold change for each timepoint p.i. compared to day 0 of CD56^bright^Ki-67^+^ NK cells out of total CD56^bright^ NK cells (left) and CD56^dim^Ki-67^+^ NK cells out of total CD56^dim^ NK cells (right) in the blood of symptomatic, asymptomatic and uninfected participants. **e**, Heatmap of correlation matrix showing Spearmanʼs *r* values for correlation of the day 7 p.i. percentages of CD56^bright^Ki-67^+^ NK cells (out of total CD56^bright^ NK cells) and CD56^dim^Ki-67^+^ NK (out of total CD56^dim^ NK cells) with the modified Jackson symptom score and day 3 and 4 p.i. innate activation markers, the percentage of IM monocytes out of total monocytes, and the CD169 and HLA-ABC MFI of monocyte and DC subgroups, for all available infected participants (day 3, *n* = 19; day 4, *n* = 16). **f**, UMAP analysis and FlowSOM clustering were undertaken on gated T cells based on the markers CD4, CD8, CD69, CD38, Ki-67, CD11a, CD49d and CXCR3. **g**, Heatmaps of marker expression by FlowSOM T cell clusters for symptomatic (top) and asymptomatic (bottom) participants at day 7. **h**, Longitudinal analysis of T cell FlowSOM cluster percentage fold change out of total T cells. **i**, Percentage fold change for each timepoint p.i. compared to day 0 of CD38^+^Ki-67^+^CD8^+^ of total CD8^+^ T cells (left) and CD38^+^Ki-67^+^CD4^+^ of total CD4^+^ T cells (right) in the blood of symptomatic, asymptomatic and uninfected participants. **j**, Heatmap of correlation matrix showing Spearmanʼs *r* values for correlation of day 7 p.i. percentages of CD38^+^Ki-67^+^CD8^+^ T cells (out of total CD8^+^ T cells) and CD38^+^Ki-67^+^CD4^+^ T cells (out of total CD4^+^ T cells) with the modified Jackson symptom score and day 3 and 4 p.i. innate activation markers, the percentage of IM monocytes out of total monocytes, and the CD169 and HLA-ABC MFI of monocyte and DC subgroups, for all available infected participants (day 3, *n* = 19; day 4, *n* = 16). For **a**,**b**,**f**,**g**, numbers of samples analyzed are detailed in the Methods. For **c**,**d**,**h**, results are shown as mean ± s.e.m. For **c**,**d**,**h**,**i**, data were generated from *n* = 18 symptomatic, *n* = 4 asymptomatic and *n* = 5 uninfected (**d**,**i** only) participants (*n* numbers per timepoint are detailed in the Methods). Significance between symptomatic and asymptomatic participants was tested by a two-way linear mixed-effects model (REML) with Geisser−Greenhouse correction, and post hoc pairwise comparisons were adjusted using the Holm−Sidak method. No statistical tests are shown for uninfected participants. For **e**,**j**, significance was assessed using Spearmanʼs rank correlation coefficient (two-sided). *P < 0.05, **P < 0.01, ***P < 0.001. D, day; FC, fold change; REML, restricted maximum likelihood.

**Extended Data Fig. 6:**
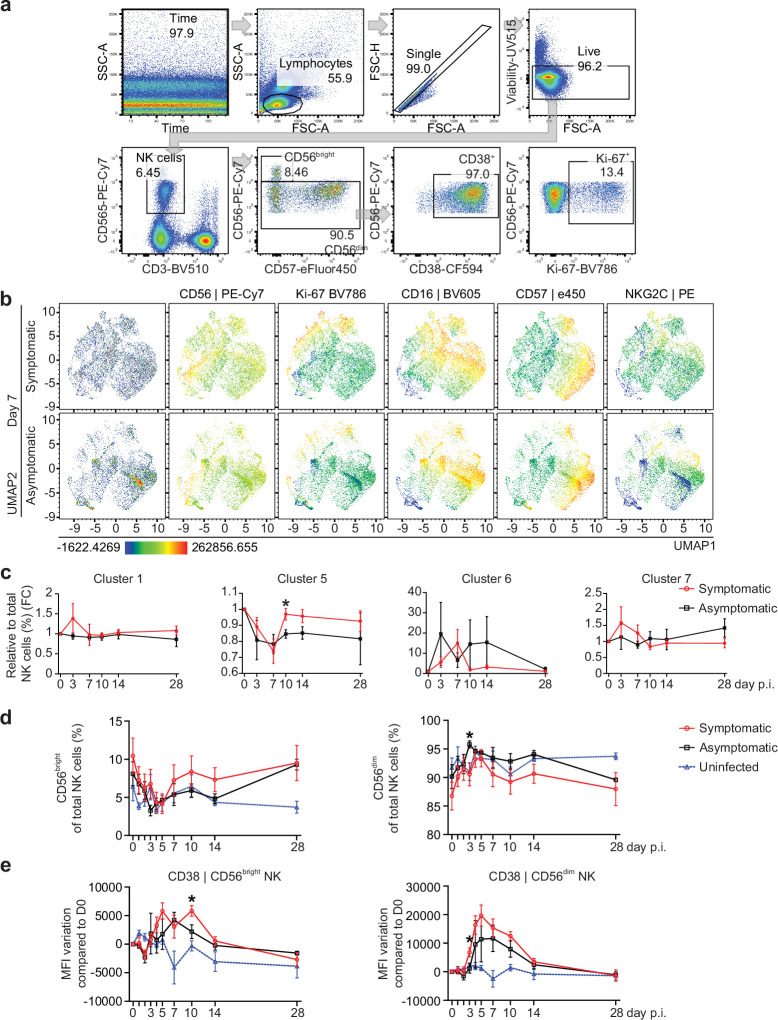
Flow cytometry gating strategy and extended dimensionality reduction and phenotyping analysis of NK cells. **a**, Gating strategy for PBMC subpopulation phenotyping. Live NK cells were assigned as CD56^+^ and have been separated into CD56^bright^ and CD56^dim^. CD38^+^ and Ki-67^+^ CD56^bright^ and CD56^dim^ NK cells have been assessed as percentage of total CD56^bright^ and CD56^dim^ NK cells, respectively. **b**, Heatmap UMAP plots showing expression of NK cell markers at day 7 p.i. **c**, Longitudinal analysis of NK cell FlowSOM cluster percentage fold-change out of total NK cells. **d,e**, Plots showing frequencies of total CD56^bright^ and CD56^dim^ out of total NK cells (**d**, data shown as percentages) and CD38 expression on CD56^bright^ and CD56^dim^ NK cells (**e**, data shown as MFI variation compared to day 0). For **b**, numbers of samples analysed are detailed in the Methods. For **c-e**, data were generated from *n* = 18 symptomatic, *n* = 4 asymptomatic and *n* = 5 uninfected participants (n numbers per timepoint are detailed in the Methods). Data are mean ± s.e.m. Significance between symptomatic and asymptomatic participants was tested by a two-way linear mixed-effects model (REML) with Geisser−Greenhouse correction and post hoc pairwise comparisons were adjusted using the Holm−Šidák method (*P < 0.05). No statistical tests are shown for uninfected participants. MFI, median fluorescence intensity; D0, day 0; REML, restricted maximum likelihood.

During the early resolution phase, a cluster of DEGs involved in cell proliferation pathways was identified distinguishing symptomatic participants from asymptomatic participants (Fig. 2d,g). We hypothesized that this second phase of the peripheral blood DEG response reflected proliferation of cells involved in viral clearance, such as natural killer (NK) and CD8^+^ T cells, and was influenced by innate activation during the acute phase. The activation of circulating NK cells was, therefore, assessed by co-expression of Ki-67 (Supplementary Table 8). UMAP and FlowSOM clustering on gated NK cells revealed four clusters with high Ki-67 expression (clusters 2, 3, 4 and 8; Fig. 4a,b and Extended Data Fig. 6a,b). The frequency of these clusters generally peaked at day 7 and was significantly higher in symptomatic participants at day 7 for clusters 2 and 8 and at day 10 p.i. for clusters 2 and 3 (Fig. 4c; clusters 1 and 5−7 in Extended Data Fig. 6c) compared to asymptomatic participants. Additionally, gated circulating NK cells were manually divided into two subgroups: CD56^bright^ and CD56^dim^ (Extended Data Fig. 6a). After infection, the frequency of the dominant CD56^dim^ NK cells increased, with a significantly higher percentage of these cells in asymptomatic participants at day 3 p.i., with corresponding decrease in CD56^bright^ NK cells (Extended Data Fig. 6d). In line with other innate cell markers, the activation marker CD38 expression on CD56^dim^ NK cells followed the pattern of quicker and greater increase in symptomatic participants (not seen in CD56^bright^ NK cells; Extended Data Fig. 6e). Furthermore, at the later day 7 timepoint, greater expansion of NK cells expressing Ki-67 was seen in both NK cell subgroups in symptomatic participants (Fig. 4d). Finally, correlation analysis revealed strongly significant correlations between the frequency of IMs at days 3−4 as well as CD169 and HLA-ABC expression on monocytes and DC subgroups with the percentage of CD56^dim^Ki-67^+^ NK cells at day 7 (Fig. 4e).

Previous studies showed correlations between pre-existing influenza-specific T cells in blood and reduced symptoms after infection^12,13,22^. To assess for potential confounding by T cell memory responses, IFNγ ELISpot was performed using cryopreserved peripheral blood mononuclear cells (PBMCs) and two previously established influenza peptide pools biased toward CD4^+^ and CD8^+^ T cells, respectively^23^. At baseline, no significant differences in IFNγ-producing CD4^+^ or CD8^+^ T cells were observed between infected and uninfected participants or between symptomatic and asymptomatic participants (Supplementary Fig. 11a–d). Furthermore, in participants who became infected, no significant correlations were observed between baseline T cell frequencies and symptom scores or VL (Supplementary Fig. 11e–h), suggesting that, for this cohort, there was no strong relationship between pre-existing T cells and clinical outcome.

**Extended Data Fig. 7:**
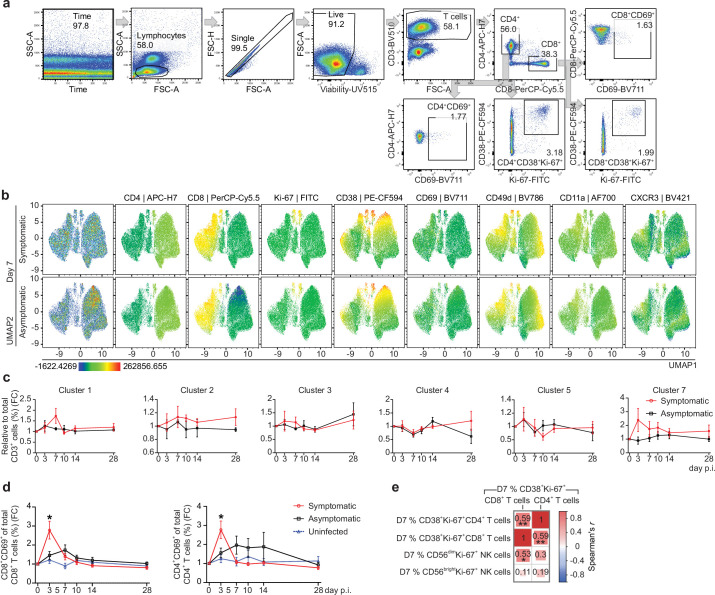
Flow cytometry gating strategy and extended dimensionality reduction and phenotyping analysis of T cells. **a**, Live T cells were assigned as CD3^+^ and have been separated into CD4^+^ and CD8^+^ T cells. CD69^+^ and CD38^+^Ki-67^+^ double positive and CD4^+^ and CD8^+^ T cells were assessed as percentage of total CD4^+^ and CD8^+^ T cells, respectively. **b**, Heatmap UMAP plots showing expression of T cell markers at day 7 p.i. **c**, Longitudinal analysis of T cell FlowSOM cluster percentage fold change out of total T cells. **d**, Plots showing frequencies of CD69^+^ CD8^+^ and CD4^+^ T cells out of total CD8^+^ and CD4^+^ T cells (data shown as percentage fold change). **e**, Heatmap of correlation matrix showing Spearman’s *r* values between day 7 p.i. percentages of CD38^+^Ki-67^+^CD8^+^ T cells (out of total CD8^+^ T cells) and CD38^+^Ki-67^+^CD4^+^ T cells (out of total CD4^+^ T cells) with each other and with day 7 p.i. percentages of CD56^bright^Ki-67^+^ NK cells (out of total CD56^bright^ NK cells) and CD56^dim^Ki-67^+^ NK (out of total CD56^dim^ NK cells), for all available infected participants (day 3: n = 19 and day 4: n = 16). Significance was assessed using Spearman’s rank correlation coefficient (two-sided; *P < 0.05, **P < 0.01). For **b-d**, numbers of samples analysed are detailed in the Methods. For **c,d**, data were generated from *n* = 18 symptomatic, *n* = 4 asymptomatic and *n* = 5 uninfected participants (*n* numbers per timepoint are detailed in the Methods). Data are mean ± s.e.m. Significance between symptomatic and asymptomatic participants was tested by a two-way linear mixed-effects model (REML) with Geisser−-Greenhouse correction and post hoc pairwise comparisons were adjusted using the Holm−Šidák method (*P < 0.05). No statistical tests are shown for uninfected participants. FC, fold change; REML, restricted maximum likelihood.

Immunophenotyping of circulating CD4^+^ and CD8^+^ T cells was undertaken by flow cytometry (Supplementary Table 8). UMAP and FlowSOM clustering revealed clusters of CD8^+^ (cluster 8) and CD4^+^ T cells (cluster 6) upregulating high levels of CD38 and Ki-67 (Fig. 4f,g and Extended Data Fig. 7a,b). In symptomatic participants, the CD8^+^ T cell cluster peaked earlier at day 7 p.i. and to significantly higher frequencies compared to asymptomatic participants (Fig. 4h; clusters 1−5 and 7 in Extended Data Fig. 7c). These patterns of activation/proliferation were supported by manual gating, with a trend toward greater increases in the frequency of CD38^+^Ki-67^+^ T cells in symptomatic participants (Fig. 4i). Furthermore, analysis of the early activation marker CD69 demonstrated significantly higher frequencies of CD69^+^CD8^+^ and CD4^+^ T cells at day 3 p.i. in symptomatic participants (Extended Data Fig. 7d). As with CD56^dim^Ki-67^+^ NK cells, the frequency of CD38^+^Ki-67^+^CD8^+^ T cells at day 7 p.i. significantly correlated with the percentage of IMs at days 3−4, along with myeloid activation marker MFI at days 3−4 (Fig. 4j). Day 7 CD38^+^Ki-67^+^CD4^+^ T cells correlated with day 7 CD38^+^Ki-67^+^CD8^+^ T cells, with the latter further correlating with day 7 Ki-67-expressing CD56^dim^ NK cells (Extended Data Fig. 7e).

Together, these findings suggest that an exaggerated innate response at the peak of influenza VL (days 3−4 p.i.) is associated with subsequently greater NK and T cell activation/proliferation, which could be responsible for the somewhat accelerated viral clearance seen in symptomatic participants.

### Integrative analyses reveal temporal dependencies across anatomical sites and immune modalities

**Fig. 5:**
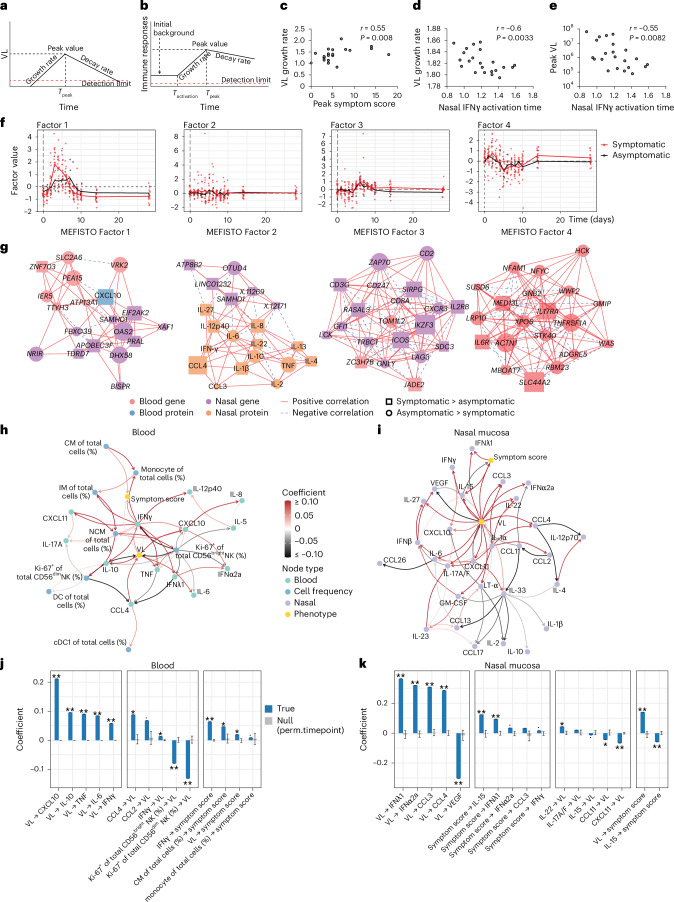
Integrative analyses reveal temporal dependencies across immune modalities. **a**,**b**, Model schematics showing VL (**a**) and immune response (**b**) kinetics analyzed by piecewise linear regression. *T*_peak_, the time point after inoculation when the measured analyte reaches its maximum value; *T*_activation_, The time point after inoculation when the measured analyte is first detected above its baseline level (or detection limit). **c**−**e**, Spearmanʼs correlation’s between VL growth rate and peak daily symptoms (**c**) or activation time of nasal IFNγ (**d**) and between peak daily VL and activation time of nasal IFNγ (**e**). Significance was assessed using Spearmanʼs rank correlation coefficient (two-sided). **f**, MEFISTO-identified factors and their temporal patterns throughout the infection for symptomatic and asymptomatic participants. Dots represent inferred factor values per participant and timepoint. **g**, MEFISTO-generated conditional dependency networks showing correlations among the top 20 features in each factor. **h**,**i**, Network representation of the conditional temporal dependencies among immune factors in the blood (**h**) and nasal mucosa (**i**) compartments, analyzed by multivariate time series modeling of *n* = 16 independent participants (13 symptomatic and three asymptomatic). Directed edges represent coefficients estimated using a VAR framework (Methods), where a positive coefficient indicates that the source node positively predicts the value of the target node at the subsequent timepoint conditional on all other features in the model. Nodes within two network steps of the ‘Viral load’ and ‘Symptom score’ nodes and the edges connecting them (absolute coefficient ≥ 0.01 and permutation-based *P* < 0.05) are shown. **j**,**k**, Top predictive edges involving ‘Viral load’ and ‘Symptom score’, in the blood (**j**) and in nasal mucosa (**k**) compartments, ranked by coefficient values. Error bars represent the mean ± s.d. of the null distribution generated by *n* = 100 permutation runs, where permutation is done by random shuffling of timepoint label pairing within each individual to disrupt temporal dependencies. For **h**−**k**, empirical two-sided *P* values were determined by comparing observed coefficients (‘True’) to null distributions generated by permutation of timepoint label pairing (100 permutations, ^·^*P* < 0.1, *P < 0.05, **P < 0.01). perm., permutation.

With these data highlighting the importance of timing for the coordinated response to infection, piecewise mixed-effects linear regression modeling was used to test the association of kinetic features, including activation time, peak time and magnitude, and growth and decay rates, with soluble mediators, VL and symptoms (Fig. 5a,b). This revealed strong correlations between VL growth rate and maximum symptom score (Fig. 5c) as well as activation time of IFNγ in the nasal mucosa with reduced VL growth rate and peak (Fig. 5d,e).

**Extended Data Fig. 8:**
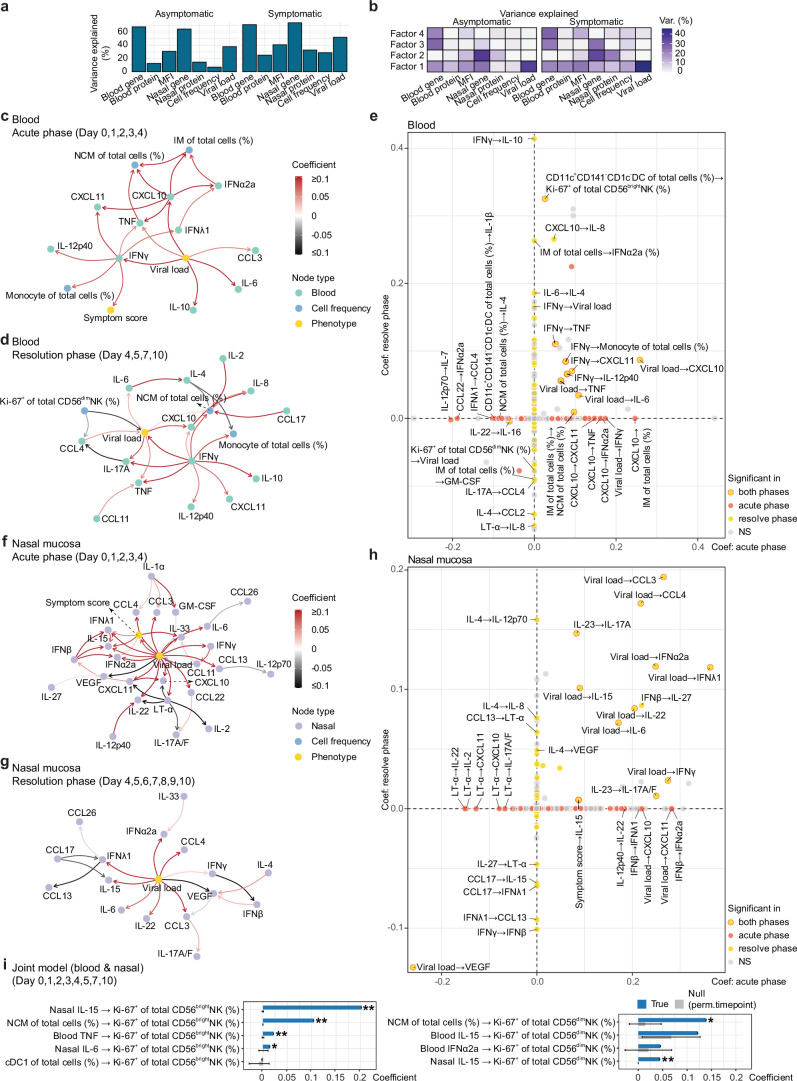
Extended integrative analyses. **a,b**, MEFISTO-generated plots showing overall variance percentages across analysed modalities for symptomatic and asymptomatic participants (**a**) and individual variance percentages per inferred factor (**b**). **c,d**, Network representation of the conditional temporal dependencies among immune factors during the acute phase (**c**) and the resolution phase (**d**) in blood, as analysed by multivariate time-series modelling of *n* = 16 independent subjects (13 symptomatic, 3 asymptomatic). **e**, Scatter plot comparing coefficient magnitude and significance level of each inferred edge between the acute-phase and resolution-phase models in blood. **f,g**, Network representation of the conditional temporal dependencies among immune factors during the acute phase (**f**) and the resolution phase (**g**) in nasal mucosa, as analysed by multivariate time-series modelling of *n* = 16 independent subjects (13 symptomatic, 3 asymptomatic). **h**, Scatter plot comparing coefficient magnitude and significance level of each inferred edge between the acute-phase and resolution-phase models in nasal mucosa. **i**, Top features predictive of subsequent of subsequent NK cell activation inferred from a joint VAR model including immune factors from both blood and nasal compartments. Error bars represent the mean ± standard deviation of the null distribution generated by n = 100 permutation runs, where permutation is done by random shuffling of timepoint label pairing within each subject to disrupt temporal dependencies. Empirical two-sided *P* values were determined by comparing observed coefficients (‘True’) to null distributions generated by permutation of timepoint label pairing (100 permutations, *P < 0.05, **P < 0.01). For **c,d,f,g**, nodes within two network steps of the ‘Viral load’ node and the edges connecting them (absolute coefficient ≥ 0.01 and permutation-based *P* < 0.05) are shown. For **e,h**, edges with permutation-based *P* < 0.05 are considered significant. For visual clarity, edge labels are shown only for edges that are significant in both acute and resolution phases, and the top five (positive and negative) edges in either acute or resolution phases. NS, not significant; perm., permutation.

To further explore potential drivers of immune response and disease control, integration of gene expression, VL, soluble mediator and cellular phenotyping data was undertaken using MEFISTO^24^ for temporal dimensionality reduction and interpolation modeling. This revealed four patterns of variation (factors) (Fig. 5f and Extended Data Fig. 8a,b) for which conditional dependency networks of the top features contributing to each factor were generated (Fig. 5g). Factor 1 showed the most pronounced differences between symptomatic and asymptomatic participants. Its top-weighted features primarily included DEGs in the nasal mucosa that limit viral replication (for example, EIF2AK2/protein kinase R, SAMHD1 and APOBEC3F higher in symptomatic participants and OAS2 higher in asymptomatic participants) and cellular proliferation/apoptosis in the blood around the time of peak VL. CXCL10 protein level in blood appeared as a central node connecting nasal and blood compartments. Although differences in Factor 2 were modest, these also centered around pro-inflammatory cytokines in the nasal mucosa. Factor 3 peaked around day 7, including genes involved in adaptive immune responses, whereas Factor 4, like Factor 1, showed an early peak for symptomatic participants, mainly including pro-inflammatory cytokine receptor genes.

To refine these putative regulatory frameworks, a multivariate time-series-based machine learning approach was developed using a vector autoregression (VAR) framework that infers directed edges between features (*x* → *y* if the state of *x* at an earlier time predicts the state of *y* at a subsequent timepoint)^25^. We excluded the large-volume gene expression data that dominated MEFISTO analysis and focused on VL, cellular and protein data from the first 10 days p.i. to infer temporal dependencies in blood and nasal samples (Fig. 5h,i). Unsurprisingly, in both compartments, top features that were positively predicted by VL were enriched for antiviral/inflammatory mediators. In blood, these included CXCL10, IFNγ and TNF (Fig. 5j), whereas, in nasal mucosa, they included IFNλ1/IL-29, IFNα2a, CCL3/MIP-1α and CCL4/MIP-1β (Fig. 5k). In blood, several features predicted symptom score, particularly IFNγ and the frequency of CMs (Fig. 5h,j), with VL also predicting symptom score (Fig. 5j,k). Notably, blood IL-10 was predicted by both VL and blood IFNγ levels (Fig. 5h). Similarly, IFNγ was connected to IM frequency, which, in turn, predicted NCM frequency, and the frequency of NCMs positively predicted subsequent NK cell proliferation/activation. This, in turn, was negatively associated with VL, consistent with a temporal relationship between NK cell activation and viral control. In the nasal compartment, VL and symptom score both predicted subsequent IL-15 (Fig. 5i,k), which is known to activate NK cells, whereas IL-15 levels negatively predicted symptom score and VL, the latter more weakly (permutation *P* = 0.059). Together, these suggest an IL-15-mediated negative feedback circuit: IL-15 increases after VL/symptoms, which then activates NK cells that reduce VL.

To assess whether the inferred temporal network differed between acute and resolution phases, we applied our VAR framework to early and late timepoints separately in blood and nasal compartments (Extended Data Fig. 8c–h). In both compartments, VL-associated pro-inflammatory/antiviral edges were observed in both early/acute and late/resolution models, suggesting broad conservation of regulatory relationships. Finally, a joint compartment model integrating blood and nasal immune features (Extended Data Fig. 8i) confirmed nasal IL-15 and blood NCMs as strong predictors of NK cell activation in the blood. Together, these data suggest that early induction of mucosal cytokines (notably IL-15) and myeloid responses drive NK cell proliferation/activation to promote viral clearance.

### Susceptibility to symptomatic influenza is associated with greater pre-infection responsiveness to innate stimulation

**Fig. 6:**
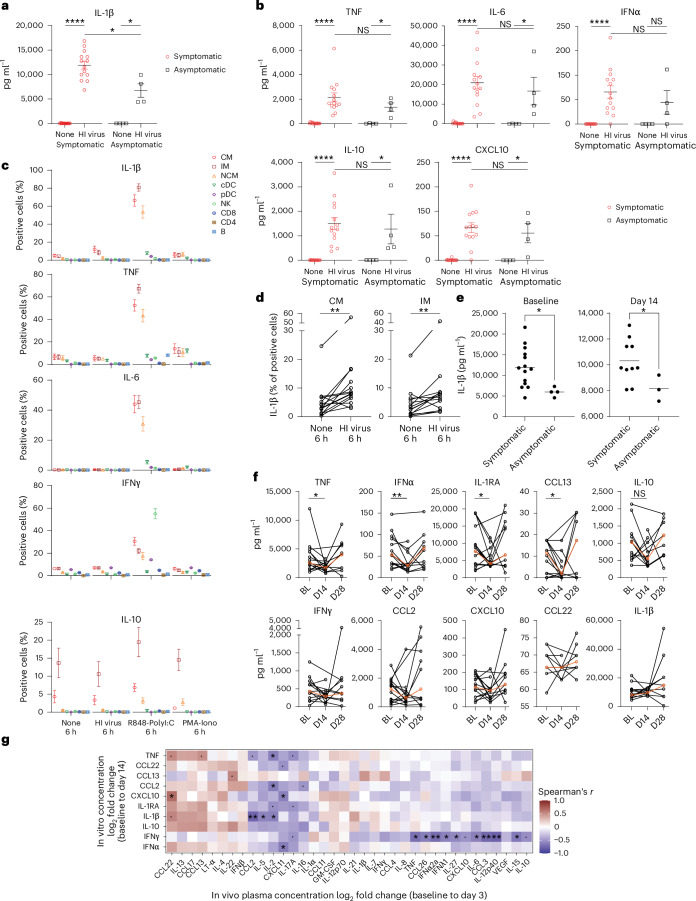
Susceptibility to symptomatic influenza is associated with greater pre-infection responsiveness to innate stimulation. **a**,**b**, IL-1β (**a**) and TNF, IL-6, IFNα, IL-10 and CXCL10 (**b**) concentrations in culture supernatants of pre-inoculation PBMCs from symptomatic (*n* = 14) and asymptomatic (*n* = 4) participants, before (none) and after (HI virus) stimulation with HI virus for 24 hours. Data are mean concentrations ±s.e.m. Significance was tested by two-sided Mann−Whitney *U*-tests. **c**, Cytokine-producing PBMC populations without or after stimulation with HI virus, TLR ligands R848 and PolyI:C or PMA and ionomycin for 6 hours, shown as percentage of cytokine-positive cells (*n* = 14). Comparison between symptomatic and asymptomatic participants was not done because of the low number of asymptomatic participants (*n* = 2 of 14 tested). No statistical tests were performed. **d**, IL-1β-producing CMs and IMs without or after stimulation with HI virus for 6 hours, shown as percentage of IL-1β-positive cells (*n* = 14). Data are mean ± s.e.m. Significance was tested by two-sided Wilcoxon matched-pairs signed-rank tests. **e**, IL-1β concentration in culture supernatants of pre-inoculation/baseline (symptomatic participants: *n* = 11, asymptomatic participants: *n* = 3) and day 14 p.i. (symptomatic participants: *n* = 10, asymptomatic participants: *n* = 3) PBMCs after stimulation with HI virus for 24 hours. Data are mean concentration ± s.e.m. Significance between symptomatic participants and asymptomatic participants was tested by two-sided Mann−Whitney *U*-tests. **f**, Cytokine and chemokine levels in culture supernatants of pre-inoculation/baseline (IL-1β, *n* = 14; rest of mediators, *n* = 15), day 14 p.i. (*n* = 13) and day 28 p.i. (*n* = 10) PBMCs of infected participants, after stimulation with HI virus for 24 hours. The orange line connects each timepointʼs median. Significance was tested by two-sided Wilcoxon matched-pairs signed-rank tests. **g**, Heatmap of Spearmanʼs correlations and corresponding two-sided *P* values between baseline to day 3 log_2_FC of soluble mediator concentrations in the blood (in vivo) and baseline to day 14 log_2_FC of soluble mediator concentrations in culture supernatants of HI virus-stimulated PBMCs (in vitro). In vivo features with low temporal variability (variance < 0.1) were excluded. For in vitro measurements, a small offset was added prior to log transformation to avoid zero values (*ε* = 0.1 × minimum non-negative value across all features). *P < 0.05, **P < 0.01, ***P < 0.001, ****P < 0.0001. BL, baseline; HI, heat-inactivated; Iono, ionomycin; NS, not significant.

**Extended Data Fig. 9:**
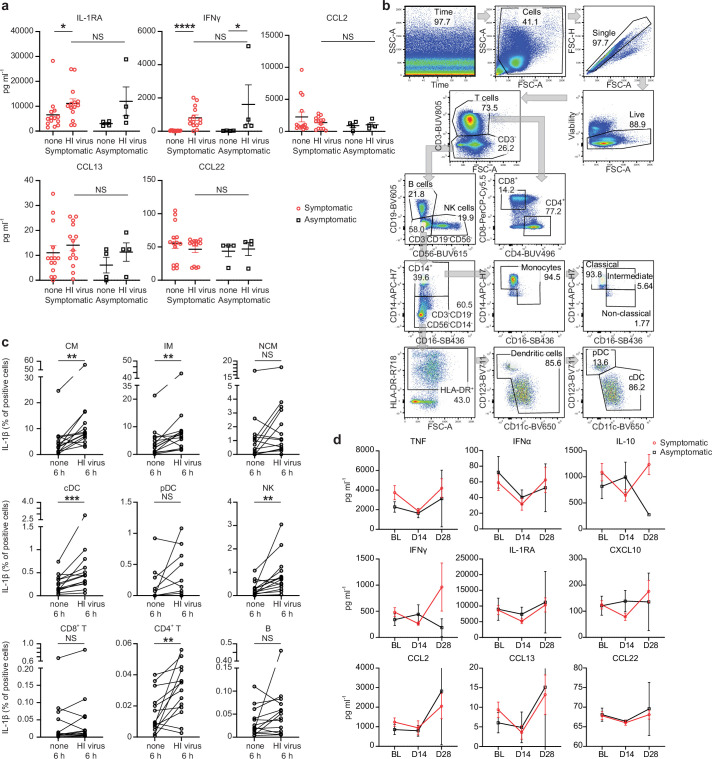
Extended soluble mediator analysis and flow cytometry gating strategy after in vitro PBMC challenge. **a**, Cytokine and chemokine levels in culture supernatants of pre-inoculation PBMCs from symptomatic (*n* = 14) and asymptomatic (*n* = 4) participants, pre- (none) and post- (HI Virus) stimulation with HI virus for 24 hours. Data are mean concentrations ± s.e.m. Significance was tested by two-sided Mann−Whitney U−tests. **b**, Gating strategy for analysis of intracellular production of cytokines in PBMCs. Live T cells were assigned as CD3^+^ and have been separated into CD4^+^ and CD8^+^ T cells. Live NK cells were assigned as CD3^−^CD56^+^ and live B cells as CD3^−^CD19^+^. Live monocytes have been assigned as CD14^+/dim^ excluding other lineage positive and CD14^dim^CD16^−^ cells and have been separated into three subgroups: CD14^+^CD16^−^ classical monocytes (CMs), CD14^+^ CD16^+^ intermediate monocytes (IMs) and CD14^dim^CD16^+^ non-classical monocytes (NCMs). Live DCs have been assigned as CD14^−^HLA-DR^+^ after excluding other lineage positive cells and separated into two populations: CD123^+^ plasmacytoid DCs (pDCs) and CD11c^+^CD123^−^ conventional DC (cDCs). **c**, IL-1β producing PBMC subpopulations without or after stimulation with HI virus for 6 hours, shown as percentage of IL-1β positive cells (*n* = 14). Data are mean ± s.e.m. Significance was tested by two-sided Wilcoxon matched-pairs signed-rank tests. **d**, Cytokine and chemokine levels in culture supernatants of pre-inoculation (symptomatics: *n* = 12, asymptomatics: *n* = 3), day 14 p.i. (symptomatics: *n* = 10, asymptomatics: *n* = 3) and day 28 p.i. (symptomatics: *n* = 8, asymptomatics: *n* = 2) PBMCs after stimulation with HI virus for 24 hours. Data are mean concentrations ± s.e.m. Significance between symptomatic and asymptomatic participants was tested by a two-way linear mixed-effects model (REML) with Geisser−Greenhouse correction and post hoc pairwise comparisons were adjusted using the Holm−Šidák method. *P < 0.05, **P < 0.01, ***P < 0.001, ****P < 0.0001. HI, heat-inactivated; NS, not significant; h, hours; BL, baseline; D, day; REML, restricted maximum likelihood.

To test the hypothesis that greater responsiveness of myeloid cells at the point of viral exposure predisposes to symptomatic disease, pre-inoculation PBMCs were cultured with heat-inactivated virus and soluble mediator concentrations measured after 24 hours. In culture supernatants, significantly higher levels of IL-1β were detected from those who would later go on to symptomatic infection (Fig. 6a). A similar trend was observed with TNF, IL-6, IFNα, IL-10 and CXCL10, although not reaching statistical significance (Fig. 6b), whereas the rest of tested mediators showed no consistent patterns (Extended Data Fig. 9a).

We next challenged available cells for 6 hours in vitro with heat-inactivated virus, Toll-like receptor (TLR) ligands that mimic viral activation or PMA/ionomycin for non-specific T cell activation, and we used flow cytometry to assess cytokines in monocytes, DCs, T cells, B cells and NK cells (Extended Data Fig. 9b). Although the number of available samples did not allow for comparison between symptomatic and asymptomatic participants, this analysis showed that monocytes were the main producers of IL-1β, TNF, IL-6 and IL-10 after TLR ligand stimulation, whereas DCs responded to a much lesser degree (Fig. 6c). NK cells were the main producers of IFNγ, followed by monocytes and pDCs, and IMs were the main producers of IL-10. Stimulation with virus induced much less cytokine production in monocytes and DCs compared to TLR ligand stimulation (Fig. 6c), with increases mainly in IL-1β production in CMs and IMs (Fig. 6d and Extended Data Fig. 9c).

Finally, to test whether differential monocyte responsiveness was intrinsically fixed or acquired and modifiable, we compared virus-stimulated soluble mediator secretion from PBMCs collected before inoculation and at days 14 and 28 p.i. from infected participants. This confirmed that virus-stimulated pre-inoculation PBMCs from symptomatic participants secreted significantly more IL-1β compared to asymptomatic participants both before inoculation and at day 14 p.i. (Fig. 6e and Extended Data Fig. 9d). However, compared to the pre-inoculation timepoint, PBMCs from day 14 p.i. showed a generalized refractoriness (Fig. 6f) with significantly lower TNF, IFNα, IL-1RA and CCL13. Of note, day 28 PBMC secretory capacity after stimulation was restored to pre-inoculation levels, indicating the transitory nature of the earlier decreases. Correlation analysis between in vivo soluble mediator concentrations during the infection and in vitro PBMC mediator secretion after viral stimulation revealed a negative correlation between day 3/baseline fold change of in vivo mediators and day 14/baseline fold change of those in vitro (mainly IFNγ), implying that early systemic innate activation after infection is associated with refractoriness to secondary viral stimulation (Fig. 6g). Thus, the sensitivity of monocytes to stimulation by viral infection is likely influenced by recent exposures, potentially explaining the varied susceptibility to symptomatic infection between individuals and within a short timeframe.

## Discussion

After controlled human influenza infection, we show how the speed and magnitude of the early innate immune response are associated with both positive and negative effects, enhancing infection resolution but also unwanted symptoms. Although participants were all ostensibly similar healthy young adults, differences in innate responsiveness at the time of virus exposure indicated individual predispositions to symptomatic or asymptomatic disease that were subsequently modulated by inflammatory signals. Differences in symptom score were not explained by variations in VL but, rather, by differential upregulation of IFN-driven transcriptional responses, which is a characteristic feature of respiratory virus infection^26,27^.

Here, this appeared more quickly in blood than in nasal mucosa, challenging the assumption that systemic responses simply track those in infected tissues. This was also observed after primary controlled human SARS-CoV-2 infection^27,28^, where activated circulating myeloid cells were likely the major contributor to this signature^29^. Differences in gene expression were paralleled by quicker induction and higher peaks of soluble mediators in symptomatic participants. Assessment of such early timepoints is rarely feasible in natural infection studies, but circulating pro-inflammatory mediators, such as CXCL10, have been shown to be raised later in severe influenza^30^. Indeed, CXCL10 appeared as a central node linking local and systemic immune features as part of our integrative analysis. Furthermore, severe disease requiring hospitalization has been associated with increased serum TNF, IL-6 and chemokines, including CCL2, CCL3, CCL4 and CCL22 (refs. ^6,31^). In our study, the clear temporal relationship between the early upregulation of soluble mediators and later symptom scores further supports a causal link and highlights their potential as prognostic biomarkers.

Immune responses in nasal mucosa early after exposure were previously linked to protection, with rapid expression of nasal TNF and IL-17 after inoculation and pre-existing CCL13 with early nasopharyngeal innate cellular recruitment associated with resistance to respiratory syncytial virus (RSV) and SARS-CoV-2 infection, respectively^29,32,33^. Here, asymptomatic infection was associated with significantly higher IL-10, CCL22 and CCL13 levels at various timepoints in the nose. This differs from natural infection studies, which have suggested higher nasal CCL7, IFNα2 and plasma IL-10 concentration as a predictor of disease severity, although, in these studies, patients are already at or after peak VL when assessed^34^. Indeed, natural infection studies previously highlighted both beneficial and harmful effects of innate cells^14,34^ as well as associations between inflammatory monocytes and disease severity^31,34^. In hospitalized patients with influenza during the 2009 pandemic, M1 monocytes were associated with more severe disease and high TNF expression after in vitro lipopolysaccharide stimulation^15^. In our system, increases in IMs, along with monocyte and DC activation, were also markers of symptom severity.

In addition, myeloid responses correlated with enhanced viral resolution characterized by cytolytic NK and CD8^+^ T cells known to directly kill virus-infected cells^35–37^. NK cells are recruited to the infected airways by local mediators, including CXCL10 and IL-15 (refs. ^2,38^), both of which were higher in symptomatic participants and highlighted as key factors in integrative analysis. Here we showed that not only increased frequency but also upregulation of activation markers CD169 and HLA-ABC correlated with subsequent T cell responses. These analyses highlight how cell-mediated immunity may be promoted by enhancing DC and monocyte cross-presentation^39–41^.

Although differences in innate responsiveness may be governed by inherited factors, they can also be altered as a result of epigenetic changes resulting in ‘trained immunity’^42^. A hallmark of this is more pronounced and rapid responses to secondary immune stimulation, a pattern seen in symptomatic infection throughout our study^43^. This has also been shown with influenza vaccine adjuvantation, which induces persistent epigenetic changes in human monocytes that enhance antiviral responses^44,45^. By contrast, PBMCs from participants 2 weeks after infection showed a generalized refractoriness in mediator secretion. This finding is supported by a previous study using the same challenge agent, where histone modifications in innate cells indicated both increased and decreased accessibility of genes including type I IFNs and IL-6 (ref. ^46^), thus showing the potential for both positive and negative manipulation of ‘trained’ innate cells.

The main limitation of this study is the participant numbers, especially in the asymptomatic group, which reflects field estimates of asymptomatic influenza infection (<20%) and is, therefore, fundamentally difficult to study^47^. To mitigate for low numbers, we used longitudinal statistical and integrative tools that increase power by considering all available timepoints. These, along with the consistency of results across a range of modalities, support the robustness of the conclusions. However, the prioritization of safety in the study design means that any extrapolation to patients with more severe or life-threatening influenza must be undertaken cautiously. Although comparisons in this study did not demonstrate significant pre-inoculation immunological differences between groups in vivo associated with complete protection, the ability to define baseline differences was likely additionally limited by the unbalanced numbers of infected and uninfected participants.

One key question is whether promoting long-lived adaptive immunity by driving innate responses (such as through more pro-inflammatory adjuvants) can be decoupled from reactogenicity. Recent evidence from preclinical studies that combine the use of adjuvant with NF-κB modulators to limit inflammation while still enhancing vaccine-induced responses suggest that this is feasible^48–50^. Our findings show the importance of early innate immune responses in finely balancing viral clearance mechanisms with the symptoms caused by inflammation. Thus, in healthy people with mild-to-moderate symptoms, these may have important socioeconomic impacts, and modulating differential early innate responses could play a critical role in limiting progression to more severe disease in high-risk patients with less reserve, with clinical implications for host-directed diagnostics and predictors of disease course, intranasal vaccines and immunomodulators for prevention or treatment.

## Methods

### Ethics statement

The study was conducted in accordance with the protocol; the consensus ethical principles derived from international guidelines, including the Declaration of Helsinki and Council for International Organizations of Medical Sciences (CIOMS) ethical guidelines; applicable International Council for Harmonisation (ICH) Good Clinical Practice guidelines; and applicable laws and regulations. The study was approved by the UK Health Research Authority (Fulham Research Ethics Committee, references 11/LO/1826 and 19/LO/1441). Written informed consent was obtained from all volunteers prior to screening and study enrollment. The study was not defined or regulated as a clinical trial as there was no investigational medicinal product, and its objective was to describe clinical and immunological responses to infection only.

### Study design and conduct

Healthy adult volunteers, aged 18−55 years with low serum neutralizing antibody levels against the challenge strain (microneutralization titer ≤1:20), were enrolled. Race, ethnicity and sex or gender information was based on self-reporting. There were no exclusions based on race, ethnicity, sex or gender, and there was equal representation of males and females (Extended Data Table 1). Participants were inoculated on day 0 with influenza A/Belgium/4217/2015 (H3N2) at a dose of 5 × 10^5^ TCID_50_, administered as nasal drops. The virus was manufactured by SGS (SGS Clinical Pharmacology Unit) according to current Good Manufacturing Practice (GMP). After inoculation, participants were quarantined until day 10 and returned for assessment and sampling at days 14, 28 and 180. Participants were combined from two sequential studies with identical inclusion/exclusion criteria and processes (ClinicalTrials.gov IDs NCT02755948 and NCT04204993). Nasal wash collected from participants at day −1 (the day before inoculation) and at day 4 p.i. was tested by multiplex PCR performed in a National Health Service (NHS) diagnostic laboratory for common respiratory viruses and bacteria (influenza A and B viruses, RSV, rhinovirus/enterovirus, parainfluenza 1−4 viruses, adenovirus, human metapneumovirus, *Bordetella pertussis*, *Bordetella holmesii*, a subset of *Bordetella bronchiseptica* and *Mycoplasma pneumoniae*). A negative PCR test at day −1 was required for inclusion in the study. Influenza infection was defined as at least two consecutive PCR-positive p.i. samples.

### Symptom assessment

Symptoms were monitored using self-reported symptom diaries. Upper respiratory, lower respiratory and systemic symptoms were scored on a scale of 0−3, and symptoms were assessed by calculating the mean (±s.e.) for each day as described by Jackson et al.^51^. Additionally, a composite total symptom score was calculated for each participant based on the Jackson symptom scoring system of eight symptoms: sore throat, sneezing, cough, nasal discharge, nasal obstruction, headache, chillness and malaise. Symptomatic participants were classified according to the modified Jackson criteria^20^ as having total adjusted Jackson symptom score of 6 or higher over the first six p.i. days and reporting having had a cold/flu or reporting nasal discharge on three or more consecutive p.i. days.

### Viral shedding

Nasal lavage samples were collected daily from each participant during the confinement period, and VL was assessed via quantitative PCR (qPCR). RNA was extracted from nasal lavage using the QIAamp Viral Mini Kit (Qiagen) following the manufacturerʼs instructions. This was carried out from a total sample volume of 250 µl. The extracted RNA was eluted in 60 µl of nuclease-free water and converted into cDNA using the High-Capacity cDNA Reverse Transcription Kit with RNase Inhibitor (Applied Biosystems), following the manufacturerʼs instructions. A T100 Thermal Cycler (Bio-Rad) was used to run cDNA conversion under the following conditions: 10 minutes at 25 °C, 120 minutes at 37 °C and 6 minutes at 85 °C. The AriaMx Real-Time PCR System (Agilent Technologies) was used to perform qPCR reaction on the converted cDNA to quantify the number of copies of viral genome, based on M gene presence. Pan-influenza A virus M gene primers and probes were used as previously (forward primer 5′-3′: GACCRATCCTGTCACCTCTGAC, reverse primer 5′-3′: AGGGCATTYTGGACAAAKCGTCTA, probe 5′-3′: TGCAGTCCTCGCTCACTGGGCACG)^18^.

### Microneutralization assay

Serum samples for antibody titration were thawed and heat inactivated. Heat-inactivated sera were subjected to two-fold serial dilutions in 96-well neutralization plates and mixed with 100 TCID_50_/50 μl of H3N2 virus. The plates were then incubated for 1 hour at 37 °C. After neutralization, the virus−serum mixtures were transferred into PBS-washed MDCK-SIAT1 cells seeded in 96-well plates and incubated for 90 minutes at 37 °C. The inoculum was then removed and replaced with plaque medium containing 1.6% carboxymethylcellulose overlay. Plates were incubated for an additional 60 hours at 37 °C. Cells were washed with PBS and fixed with 4% paraformaldehyde, followed by permeabilization with 0.5% Triton X-100. ELISA-style staining was performed using a primary anti-influenza A virus nucleoprotein antibody (Tokyo Chemical Industry), followed by an HRP-conjugated goat anti-mouse secondary antibody (SeraCare). After color development with TMB substrate and stop of the reaction with 2 N sulfuric acid, the content was transferred to new plates for reading. Absorbance was measured at 450 nm using an Omega microplate reader.

### Transcriptional profiling

Whole blood and nasal scrape samples were collected for RNA sequencing before inoculation and on days 1, 2, 3, 7, 10 and 14 after challenge. Blood samples were collected into PAXgene tubes (BD Biosciences). Nasal scrape samples were collected into RNAse/DNAse-free cryotubes containing TRIzol (Invitrogen), using Rhino-Pro curettes (Arlington Scientific). Nasal scrape samples from 21 participants and blood samples from 19 participants were sent for sequencing by a commercial provider (GENEWIZ). Two individuals who were found to be co-infected by rhinovirus, and one who seroconverted in the interval period between screening and inoculation, were excluded from analysis. After quality control, one and three blood and nasal scrape samples, respectively, were also excluded from analysis. Total RNA was extracted from PAXgene tubes using the QIAsymphony PAXgene Blood RNA Kit (Qiagen). After total RNA extraction, DNase I treatment and cleanup was undertaken using the RNA Clean and Concentrator-96 Kit (Zymo Research), and NEBNext Globin & rRNA Depletion Kits (New England Biolabs) were used for globin mRNA and rRNA depletion. For both the whole blood and nasal scrape RNA samples, DNA library construction was performed using the NEBNext Ultra II Directional RNA Library Prep Kit (New England Biolabs). Blood and nasal samples were sequenced using the Illumina NovaSeq 6000 with the NovaSeq 6000 S2 kit (200 cycles) and the Illumina HiSeq 4000 with the HiSeq 3000/4000 SBS kit, respectively. FastQC was used for the quality control for the reads of blood and nasal samples. After trimming adapter sequences using Trimmomatic (version 0.36), STAR aligner (version 2.7.1a) was used to map blood sample reads to the human GRCh38 reference genome. STAR (version 2.7.10a) and reference genome human GRCh38.p13 were used for nasal sample reads mapping. After mapping, transcript quantifications were performed using the featureCounts tool from the Subread package (version 1.5.2) to generate read counts.

### Transcriptomic analysis

Downstream analyses were conducted in R version 4.1.3. Annotation with Ensembl gene ID and gene names was performed using the biomaRt Bioconductor package (version 2.50.3)^52,53^. Lowly expressed genes (total counts <10), globin and ribosomal genes were filtered out, and the remaining genes were then normalized using the DESeq2 package (version 1.34.0)^54^. Normalized genes with fewer than three samples with a normalized read count of at least 20 were also considered lowly expressed and were removed, leaving a total of 18,006 genes for subsequent analyses. Principal component analysis was performed on the normalized counts and visualized using ggplot2 (version 3.3.6)^55^. DESeq2 was used for differential gene expression analysis between day 0 (pre-inoculation, baseline) and each day p.i. expression samples, using default parameters. Adjustment for false discovery rate was performed using the Benjamini−Hochberg procedure^56^ with genes having Benjamini−Hochberg *P*_adj_ < 0.05 considered significantly differentially expressed. The log_2_ fold change differences of DEGs (log_2_ fold change > 2 and *P*_adj_ < 0.01) were standardized to *z*-scores, for heatmap visualization using the ComplexHeatmap package (version 2.10.0)^57^. The DEGs identified above (*P*_adj_ < 0.05) were subjected to pathway enrichment analysis using IPA (Qiagen). Significantly enriched pathways (Benjamini−Hochberg *P*_adj_ < 0.05) were identified, and, for each pathway, the number of involved DEGs and IPA *z*-scores (direction and magnitude of enrichment) were visualized using the ggplot2 package. Longitudinal differential gene expression analyses were conducted using the maSigPro package (version 1.66.0)^58^, which conducts a two-step regression model to identify DEGs (Benjamini−Hochberg *P*_adj_ < 0.05) between groups and over time. The package then uses hierarchical clustering to cluster together significant DEGs that follow similar temporal expression patterns, providing pattern visualization and lists of clustered genes. For each gene cluster, pathway enrichment analysis was performed using IPA and visualized using ggplot2. Correlations between expression levels of DEGs and symptom scores were calculated with Spearmanʼs correlation test using GraphPad Prism (version 10.1.1) and visualized using the corrplot package (version 0.92)^59^.

### Soluble mediators

Cytokine and chemokine concentrations in cryopreserved plasma and nasosorption (nasal lining fluid) samples were measured using Meso Scale Discovery (MSD) multiplex immunoassays (Meso Scale Diagnostics). Four panels were used per the manufacturer’s instructions: (1) V-PLEX Cytokine Panel 1 Human Kit (Cytokine Panel), GM-CSF, IL-1α, IL-5, IL-7, IL-12, IL-15, IL-16, IL-17A, LT-α (lymphotoxin-α), VEGF; (2) V-PLEX Chemokine Panel 1 Human Kit (Chemokine Panel), CCL11 (eotaxin), CCL4 (MIP-1β), CCL26 (eotaxin-3), CCL17 (TARC), CXCL10 (IP-10), CCL3 (MIP-1α), IL-8, CCL2 (MCP-1), CCL22 (MDC), CCL13 (MCP-4); (3) V-PLEX Pro-inflammatory Panel 1 Human Kit (Pro-inflammatory Panel), IFNγ, IL-10, IL-12p70, IL-13, IL-1β, IL-2, IL-4, IL-6, IL-8, TNF; and (4) U-PLEX Panel IFNα2a, CXCL11 (I-TAC), IL-17, IL-21, IL-22, IL-23, IL-27, IFNλ1 (IL-29), IL-33, IFNβ. Undetectable sample values were assigned the average lower limit of detection (LLOD) among all plates.

### Flow cytometry

Whole blood samples collected with heparin-containing tubes were diluted 1: 1 in PBS and overlayered onto Histopaque 1077 (Sigma-Aldrich) and centrifuged for 30 minutes at 400*g*, following the manufacturer’s instructions. Immediately after isolation, PBMCs were aliquoted in cell suspensions of 1 ml, at 5−10 × 10^6^ per ml, into freezing tubes (Corning) and stored in FBS (Sigma-Aldrich) supplemented with 10% DMSO at −80 °C overnight and then transferred into liquid nitrogen until use. Antibody panels for the analysis of the phenotype and activation status of monocytes and DCs, T cells and NK cells are detailed in Supplementary Table 8. UMAP and FlowSOM analysis on ‘Innate cell panel’ markers was performed on samples from symptomatic participants on days 0, 10, 14 (*n* = 16) and days 1, 3, 5, 7, 28 (*n* = 12) and from asymptomatic participants on days 0, 3, 7, 10, 14 (*n* = 4), days 1, 5 (*n* = 3) and day 28 (*n* = 2). UMAP and FlowSOM analysis on ‘NK cell panel’ markers was performed on samples from symptomatic participants on days 0, 10, 14 (*n* = 16), day 3 (*n* = 12), day 7 (*n* = 15) and day 28 (*n* = 8) and from asymptomatic participants on days 0, 3, 7, 10, 14 (*n* = 4) and day 28 (*n* = 2) and on ‘T cell panel’ markers on samples from symptomatic participants on days 0, 7, 10, 14 (*n* = 16) and days 3 and 28 (*n* = 12) and from asymptomatic participants on days 0, 3, 7, 10, 14 (*n* = 4) and day 28 (*n* = 2). Samples used in dimensionality reduction were reduced in event count prior to analysis and did not include samples from uninfected participants. Longitudinal data from manually gated cell subsets of all three panels were generated from a total *n* = 18 symptomatic participants (days 0, 10, 14: *n* = 18, days 1, 2, 4, 5: *n* = 13; day 3: *n* = 15, day 7: *n* = 16 and day 28: *n* = 14), *n* = 4 asymptomatic participants (days 0, 3, 7, 10, 14: *n* = 4, days 1, 2, 4, 5: *n* = 3 and day 28: *n* = 2) and *n* = 5 for those who remained uninfected (days 0, 7, 10, 14, 28: *n* = 5, days 1, 2, 4, 5: *n* = 3 and day 3: *n* = 4). For eight participants (five symptomatic, one asymptomatic and two uninfected), sampling on days 1, 2, 4, 5 was not included in the protocol. There were no available samples from day 3 of three symptomatic participants and one uninfected participant and from day 7 of two symptomatic participants. No samples were collected on day 28 from five participants (three symptomatic and two asymptomatic) due to the COVID-19 pandemic lockdown in UK in March 2020. Data are not shown for samples that were insufficient or failed quality control (low viability or acquisition problem). In brief, cryopreserved PBMCs were thawed in warm RPMI 1640 (Sigma-Aldrich), washed and counted using the Countess II cell counter (Thermo Fisher Scientific). Up to 10^6^ PBMCs per condition were stained with LIVE/DEAD Fix Aqua Kit (Invitrogen), for panel (1), or Zombie UV Fixable Viability Kit (BioLegend), for panels (2) and (3), for 20 minutes at room temperature followed by blockade of nonspecific binding using FcBlock (Miltenyi Biotec) for 10 minutes and then incubated with antibodies against surface markers in FACS buffer (PBS supplemented with 2% FBS and 2 mM EDTA) at 4 °C for 30 minutes. After washing with FACS buffer, cells were either fixed with BD CellFIX (BD Biosciences), for panel (1), or fixed and permeabilized for intracellular staining using Foxp3/Transcription Factor Staining Buffer Set (Thermo Fisher Scientific), for panels (2) and (3). Cells were washed in FACS buffer or permeabilization buffer and stored in FACS buffer until acquisition. Data were acquired using a FACSymphony A3 (BD Biosciences) and analyzed using FlowJo (version 10.10), DownSample (3.3.1 plugin), UMAP_R (version 4.0.4 plugin) and FlowSOM (version 4.1.0 plugin) software.

### ELISpot

Cryopreserved peri-inoculation PBMCs were thawed and counted. Due to insufficient pre-inoculation samples, PBMCs from day 1 and day 2 p.i. previously shown to precede T cell activation were used for a subset of participants (symptomatic: *n* = 6 pre-inoculation, *n* = 6 day 1 p.i. and *n* = 3 day 2 p.i.; asymptomatic: n = 2 pre-inoculation and *n* = 1 day 1 p.i.; uninfected: *n* = 2 pre-inoculation and *n* = 2 day 1). MultiScreen-IP plates (Millipore) were coated media only (negative control) or with 1 μg ml^−1^ anti-CD3 antibody (Tonbo Biosciences, positive control) or peptide pools and blocked for up to 8 hours before 2.5 × 10^5^ cells were added per condition in triplicate and incubated for 18−20 hours at 37 °C in a 5% CO_2_ incubator. Two separate universal influenza peptide megapools biased toward CD4^+^ and CD8^+^ T cells were used at 1 µg ml^−1^. These pools contained a combination of previously experimentally defined epitopes covering the influenza virus proteome available in the Immune Epitope Database (IEDB). IFNγ ELISpot antibody kit (Mabtech) was used for development before being read using an AID vSpot Spectrum Analyzer.

### Integrative analyses

Piecewise mixed-effects modeling was performed as previously described^28^. To appropriately handle censored observations (values below the limit of detection) in the data, censored mixed-effects regression was used to fit the VL model. All model fitting was performed using MonolixR2019b. The resulting individual parameter estimates were then used in correlation analyses.

MEFISTO analysis was performed after filtering the data to remove features that did not change significantly during the course of the infection and scaling the different data modalities (described as ‘views’ in the MEFISTO framework) (mean = 0, s.d. = 1). As the weights calculated for each factor are also dependent on the number of features in the view, an initial run of MEFISTO gave a set of four factors dominated almost exclusively by gene expression features. Gene expression features (from both blood and nasal mucosa) were, therefore, ranked by their initial weighting; the top 20 genes were selected by weight for each factor; and the model was rerun, resulting in all views capturing a significant proportion of the total variance. The model configuration for both the initial and the final analysis is shown in Supplementary Table 9. To investigate further the relationships among the main features contributing to each latent factor, network-based conditional dependency analysis was performed using the R package GeneNet. The top 20 features with the highest positive weights were selected to determine the correlation among the factors. Pairwise partial correlations were estimated using GeneNetʼs shrinkage-based covariance methodology. Then, the conditional dependency network for these features between the two groups was visualized using a threshold of 0.1 to retain only strong dependencies and improve readability of the graphs.

The VAR framework was used to estimate from longitudinal data how immune features at a given timepoint predict feature values at the subsequent timepoint. Because sampling was irregular for some assays, the model was formulated using paired observations between consecutive sampled timepoints rather than assuming a fully observed evenly sampled time series. For each participant, feature vectors at timepoint *t* were paired with feature vectors at the next available timepoint *t* + 1, and all such participant-level pairs were stacked to estimate a group-level transition relationship. This framework makes several key assumptions. First, dependencies are estimated at the group level, assuming a shared transition structure across participants. Second, only first-order temporal dependencies are modeled—that is, features depend only on features one timestep before. Third, transition coefficients are assumed to be time invariant within each model, reflecting an average dependency structure over the modeled phase. For each target feature, regression coefficients relating all features at time *t* to the target feature at *t* + 1 were estimated using LASSO implemented in the glmnet R package (version 4.1.8). The penalty parameter (*λ*) was selected from range 10^−4^ to 1 using five-fold cross-validation. For a given *λ*, a full coefficient matrix was estimated in which all target-feature regressions shared the same *λ*. The optimal *λ* was chosen to minimize the mean squared prediction error averaged across all target-feature regressions and cross-validation folds. Permutation-based testing was used to assess the statistical significance of inferred coefficients (directed ‘edges’ from source to target features). Specifically, the pairing between predictor timepoints (*t*) and response timepoints (*t* + 1) was permuted within participants to generate null distributions that disrupt temporal dependencies but preserve inter-participant structure (100 permutations), against which empirical *P* values were computed. Participants in the uninfected group and participants with longitudinal measurements missing in more than 5 days within the first 10 days were excluded, resulting in 16 participants (13 symptomatic and three asymptomatic). Immune features with low temporal variability (zero temporal variance in more than half of the participants) were filtered. Flow cytometry features measured only at sparse timepoints (days 0, 3, 7 and 10) were also excluded. Prior to model fitting, raw MSD (soluble mediators) data were log_2_ normalized to stabilize variance, and all features were standardized (mean = 0, s.d. = 1). Separate models were fitted for different compartments and infection phases (Supplementary Table 10). Blood and nasal models were first fitted separately using all available timepoints within each compartment to infer compartment-specific conditional temporal dependencies. To investigate phase-specific emergence of inferred dependencies, additional models were fitted separately for the acute and resolution phases, and a joint model incorporating immune features from both blood and nasal compartments was fitted to identify cross-compartment predictive relationships.

### PBMC stimulation in vitro and soluble mediator measurement

Cryopreserved PBMCs were defrosted and reconstituted in RPMI 1640 culture medium (Sigma-Aldrich) supplemented with 100 U ml^−1^ penicillin−streptomycin and 10 mM L-glutamine (Gibco). In vitro stimulations were performed in the presence of 2% human AB serum (Sigma-Aldrich) and 10 mM sodium pyruvate (Gibco). Stimulants for different conditions included heat-inactivated influenza A/Belgium/4217/2015 (H3N2) virus at 7.5 × 10^5^ plaque-forming units per milliliter (PFU ml^−1^), TLR3 ligand PolyI:C (Tocris Bioscience) at 10 μg ml^−1^ and TLR7/8 ligand R848/resiquimod (Sigma-Aldrich) at 3 μg ml^−1^. In total, 3 × 10^5^ cells per condition were incubated with or without stimulants in final volume of 200 µl in sealed 96-well round-bottom plates at 37 °C with 5% CO_2_ for 24 hours. After incubation, culture supernatants were collected and stored at −80 °C. The soluble mediators CCL2, CCL13, CCL22, CXCL10, IFNα, IFNγ, IL-1β, IL-1RA, IL-6, IL-10 and TNF were measured in stored supernatants using a customized Luminex Discovery Assay Human Premixed Multi-Analyte Kit (R&D Systems) according to the manufacturerʼs instructions, followed by plate reading using Bio-Plex 200 system (Bio-Rad).

### Flow cytometry after in vitro PBMC stimulation

In brief, PBMCs were defrosted and reconstituted in RPMI 1640 culture medium (Sigma-Aldrich) supplemented with 10U ml^−1^ penicillin−streptomycin and 10 mM L-glutamine (Gibco). Cell resting and in vitro stimulations were performed in the presence of 2% human AB serum (Sigma-Aldrich) and 10 nM sodium pyruvate (Gibco). Reconstituted PBMCs were rested at a concentration of 10^6^ cells per milliliter within 5-ml polypropylene tubes (Corning) at 37 °C with 5% CO_2_ for 2 hours. After resting, PBMCs were resuspended with 100 μl of media and further incubated with 100 μl of different stimulants. Stimulants for different conditions included heat-inactivated influenza A/Belgium/4217/2015 (H3N2) virus at 7.5 × 10^5^ PFU ml^−1^, TLR3 ligand PolyI:C (Tocris Bioscience) at 10 μg ml^−1^ and TLR7/8 ligand R848/resiquimod (Sigma-Aldrich) at 3 μg ml^−1^, PMA at 5 ng ml^−1^, ionomycin at 500 ng ml^−1^ and media only for unstimulated negative control. In total, 10^6^ cells per condition were incubated with or without stimulants in a final volume of 200 µl in capped 5-ml polypropylene tubes at 37 °C with 5% CO_2_ for 2 hours. Brefeldin A (eBioscience) at 3 µg ml^−1^ was then added to all conditions, and the tubes were returned to incubation for a further 4 hours. After incubation, cells were collected and transferred into sealed 96-well round-bottom plates for FcR blocking (Miltenyi Biotec), LIVE/DEAD Fix Aqua Kit viability staining (Invitrogen), extracellular staining, fixation and permeabilization (BD Biosciences) and intracellular staining. Surface markers were CD14, CD3, CD11c, CD16, CD19, CD56, CD123, HLA-DR, CD8 and CD4, and intracellular markers were IL-1β, TNF, IL-6, IFNγ and IL-10 (detailed in Supplementary Table 8). Stained samples were stored at 4 °C until acquisition using a FACSymphony A3 (BD Biosciences) and analyzed using FlowJo (version 10.10).

### Statistics and reproducibility

Statistical analysis was performed using GraphPad Prism version 10.1.1 and R version 4.1.3. Intra-participant and group differences during the course of the infection are described in the figure legends, with exact *P* values provided in the Supplementary Information. Correlations between analytes were examined using Spearmanʼs rank correlation to investigate possible causal relationships. Statistical significance was defined as *P* < 0.05, and *P* values were adjusted for multiple comparisons, where applicable, using the Benjamini−Hochberg or the Holm−Sidak methods. Missing data in soluble mediator measurement due to undetectable sample values were assigned the average LLOD among all plates tested for each mediator. Missing data in other modalities, due to unavailable samples or failed quality control, were not imputed, and summary statistics were based on observed data.

This study is a controlled human influenza infection post hoc analysis of participants allocated in groups based on their p.i. clinical outcome. No statistical method was used to predetermine sample size, and the number of enrolled participants was based on the previously established attack rate of 75% using this challenge virus, giving a number of more than 20 infected individuals. The investigators were not blinded to clinical outcome assessment and sample allocation during experiments.

### Reporting summary

Further information on research design is available in the Nature Portfolio Reporting Summary linked to this article.

## Online content

Any methods, additional references, Nature Portfolio reporting summaries, source data, extended data, supplementary information, acknowledgements, peer review information; details of author contributions and competing interests; and statements of data and code availability are available at 10.1038/s41591-026-04483-7.

## Supplementary information

Supplementary Information Figs. 1−11.

Reporting Summary

Peer Review File

Tables 1−10.

Supplementary exact P values Exact significant *P* values (<0.05) from figures, extended data figures and supplementary figures.

## Figures and Tables

**Extended Data Table 1:**
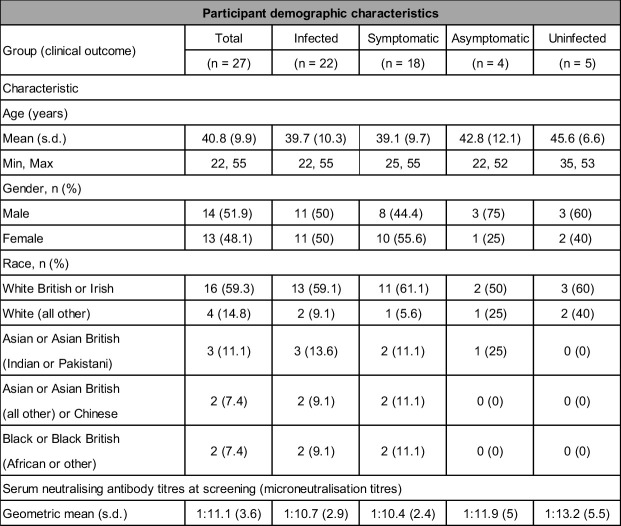
Participant demographic characteristics

## Data Availability

Individual participant data that underlie the results reported in this article after deidentification will be made available for individual participant data meta-analysis beginning 12 months and ending 5 years after article publication upon written request. Proposals should be directed to c.chiu@imperial.ac.uk. To gain access, data requestors will need to complete a data request form and sign a data access agreement. In compliance with data privacy restrictions, raw RNA sequencing data from blood samples are under managed access at the European Genome-Phenome Archive (https://ega-archive.org; accession number: EGAD50000000956). Data will be available for investigators whose request will be within the scope of participant consent subject to a data access agreement. RNA sequencing count data from nasal samples are available in the BioStudies database (https://www.ebi.ac.uk/biostudies/; accession number E-MTAB-13038).

## Code availability

MEFISTO codes are available on https://github.com/TruptiAG/MEFISTO. VAR framework codes are available on https://github.com/ah2426/h3n2-challenge.
