# Supplementary material for: Innate immune responsiveness predicts enhanced cellular immunity and symptomatic disease after controlled human influenza infection
Source: Nat Med. 2026 Jul 1;32(7):2556–69. doi: 10.1038/s41591-026-04483-7 (PMC13375583; doi:10.1038/s41591-026-04483-7)
Supplement: Supplementary file 1 — Figs. 1−11. [file 41591_2026_4483_MOESM1_ESM.pdf]

nature medicine

Supplementary information https://doi.org/10.1038/s41591-026-04483-7

Innate immune responsiveness predicts enhanced cellular immunity and symptomatic disease after controlled human influenza infection

In the format provided by the authors and unedited

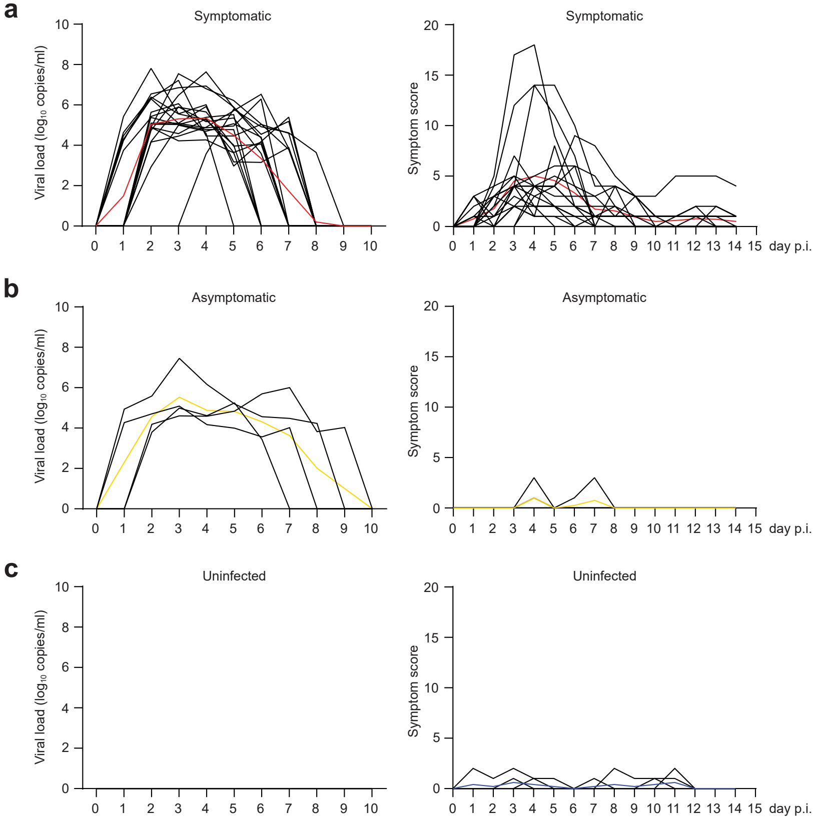

Supplementary Fig. 1. Individual viral loads and symptom scores. a-c, Individual viral shedding and symptom scores for symptomatic (n = 22) (**a**), asymptomatic (n = 4) (**b**), and uninfected (n = 5) participants (**c**). Coloured lines connect mean values per day.

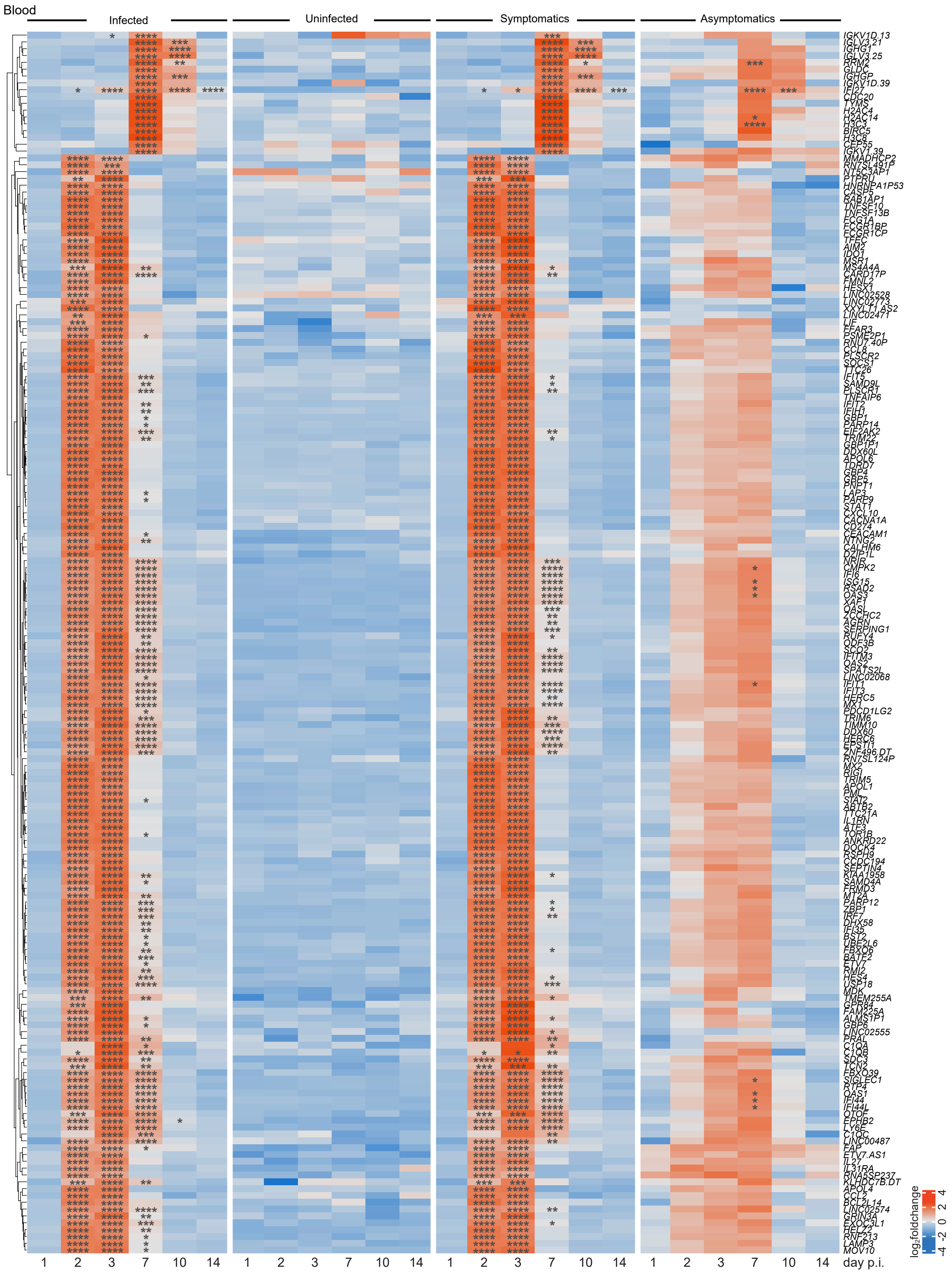

Blood
Infected
Uninfected
Symptomatics
Asymptomatics
IGKV1D.13
IGLV3.21
IGHG1
IGLV3.25
RRM2
GLDC
IGHGP
IGKV1D.39
IFI27
CDC20
TYMS
H2AC4
H2AC14
H3C3
BIRC5
H3C8
CEP55
IGKV1.39
MMADHCP2
RN7SL491P
NT5C3AP1
PTPRU
HNRNPA1P53
CASP5
RAB1AP1
TNFSF10
TNFSF13B
FCG1A
FCGR1BP
FCGR1CP
TFEC
AIM2
IDO1
MSR1
MS4A4A
CARD17P
FMNL2
HESX1
LINC02528
LINC02773
XXYLT1.AS2
LINC02471
LIF
FFAR3
PSME2P1
RNU7.40P
CCL8
PLSCR2
SOCS1
TTC26
IFIT5
SAMD9L
PLSCR1
TNFAIP6
IFIT2
IFIH1
GBP1
PARP14
EIF2AK2
TRIM22
GBP1P1
DDX60L
APOL6
TDRD7
GBP4
GBP5
PNPT1
LAP3
PARP9
STAT1
CXCL10
CACNA1A
CD274
CEACAM1
NTNG2
CALHM6
DZIP1L
NRIR
CMPK2
IFI6
ISG15
RSAD2
OAS3
XAF1
OASL
ZCCHC2
AGRN
SERPING1
RUFY4
ODF3B
SCO2
IFITM3
OAS2
SPATS2L
LINC02068
IFIT1
IFIT3
HERC5
MX1
PDCD1LG2
TRIM6
TIMM10
DDX60
HERC6
EPSTI1
ZNF496.DT
RN7SL124P
MX2
RIGI
TRIM5
APOL1
PML
STAT2
ABTB2
TTC21A
IL1RN
ATF3
TOR1B
ANKRD22
DOCK4
RSPH9
CCDC194
SEPTIN4
KIAA1958
SAMD4A
FRMD3
MT2A
PARP12
ZBP1
IRF7
DHX58
IFI35
BST2
UBE2L6
FBXO6
BATF2
ETV7
RMI2
HES4
USP18
MDK
TMEM255A
GPR84
FAM225A
ALMS1P1
GBP6
LINC02555
PRAL
C1QA
C1QB
SDC3
TCN2
FBXO39
SIGLEC1
RTP4
OAS1
IFI44
IFI44L
OTOF
EPHB2
LY6E
C1QC
LINC00487
FAP
ETV7.AS1
IL27
IL31RA
RNA5SP237
KLHDC7B.DT
APOL4
CCL2
BCL2L14
LINC02574
GRIN3A
EXOC3L1
HELZ2
RNF213
LAMP3
MOV10
1 2 3 7 10 14
1 2 3 7 10 14
1 2 3 7 10 14
1 2 3 7 10 14
day p.i.
log_2foldchange
-4 -2 0 2 4

Supplementary Fig. 2. Heatmap representation of the top DEGs in the blood post-inoculation. High-definition heatmap representation of the top DEGs ($\log_2$ fold-change > 2 and $P_{adj} < 0.01$) in the blood (n = 179) post-inoculation. The *P*-values reported are derived from DESeq2 analysis and indicate the statistical significance of differences in gene expression levels (normalised mean counts) between each post-inoculation timepoint and baseline (*$P < 0.05$, **$P < 0.01$, ***$P < 0.001$, ****$P < 0.0001$).

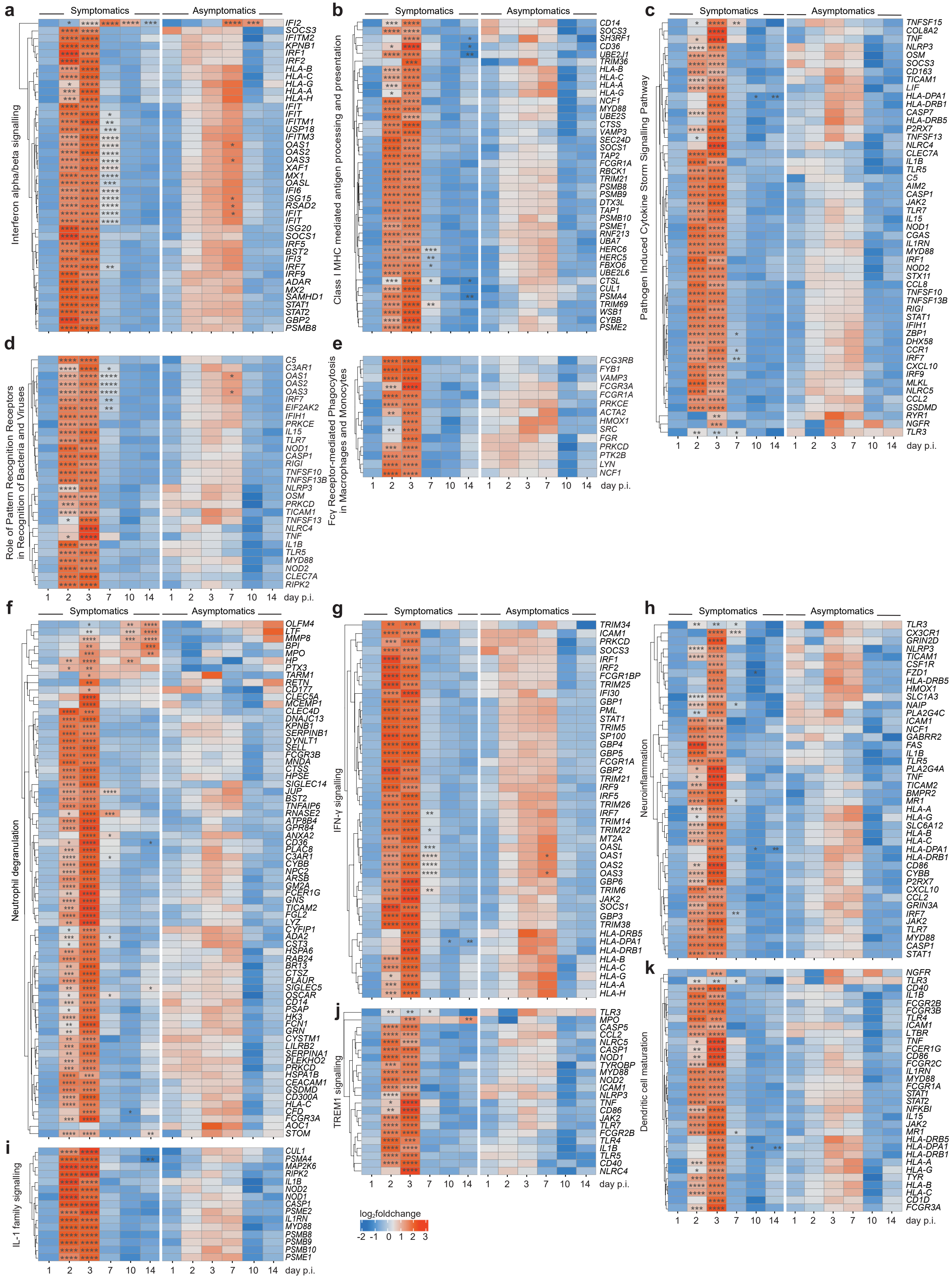

a
Symptomatics
Asymptomatics
Interferon alpha/beta signalling
IFI2 SOCS3 IFITM2 KPNB1 IRF1 IRF2 HLA-B HLA-C HLA-G HLA-A HLA-H IFIT IFIT IFITM1 USP18 IFITM3 OAS1 OAS2 OAS3 XAF1 MX1 OASL IFI6 ISG15 RSAD2 IFIT IFIT ISG20 SOCS1 IRF5 BST2 IFI3 IRF7 IRF9 ADAR MX2 SAMHD1 STAT1 STAT2 GBP2 PSMB8
b
Symptomatics
Asymptomatics
Class I MHC mediated antigen processing and presentation
CD14 SOCS3 SH3RF1 CD36 UBE2J1 TRIM36 HLA-B HLA-C HLA-A HLA-G NCF1 MYD88 UBE2S CTSS VAMP3 SEC24D SOCS1 TAP2 FCGR1A RBCK1 TRIM21 PSMB8 PSMB9 DTX3L TAP1 PSMB10 PSME1 RNF213 UBA7 HERC6 HERC5 FBXO6 UBE2L6 CTSL CUL1 PSMA4 TRIM69 WSB1 CYBB PSME2
c
Symptomatics
Asymptomatics
Pathogen Induced Cytokine Storm Signalling Pathway
TNFSF15 COL8A2 TNF NLRP3 OSM SOCS3 CD163 TICAM1 LIF HLA-DPA1 HLA-DRB1 CASP7 HLA-DRB5 P2RX7 TNFSF13 NLRC4 CLEC7A IL1B TLR5 C5 AIM2 CASP1 JAK2 TLR7 IL15 NOD1 CGAS IL1RN MYD88 IRF1 NOD2 STX11 CCL8 TNFSF10 TNFSF13B RIGI STAT1 IFIH1 ZBP1 DHX58 CCR1 IRF7 CXCL10 IRF9 MLKL NLRC5 CCL2 GSDMD RYR1 NGFR TLR3
1 2 3 7 10 14 1 2 3 7 10 14 day p.i.
d
Role of Pattern Recognition Receptors in Recognition of Bacteria and Viruses
C5 C3AR1 OAS1 OAS2 OAS3 IRF7 EIF2AK2 IFIH1 PRKCE IL15 TLR7 NOD1 CASP1 RIGI TNFSF10 TNFSF13B NLRP3 OSM PRKCD TICAM1 TNFSF13 NLRC4 TNF IL1B TLR5 MYD88 NOD2 CLEC7A RIPK2
1 2 3 7 10 14 1 2 3 7 10 14 day p.i.
e
Fcγ Receptor-mediated Phagocytosis in Macrophages and Monocytes
FCG3RB FYB1 VAMP3 FCGR3A FCGR1A PRKCE ACTA2 HMOX1 SRC FGR PRKCD PTK2B LYN NCF1
1 2 3 7 10 14 1 2 3 7 10 14 day p.i.
f
Symptomatics
Asymptomatics
Neutrophil degranulation
OLFM4 LTF MMP8 BPI MPO HP PTX3 TARM1 RETN CD177 CLEC5A MCEMP1 CLEC4D DNAJC13 KPNB1 SERPINB1 DYNLT1 SELL FCGR3B MNDA CTSS HPSE SIGLEC14 JUP BST2 TNFAIP6 RNASE2 ATP8B4 GPR84 ANXA2 CD36 PLAC8 C3AR1 CYBB NPC2 ARSB GM2A FCER1G GNS TICAM2 FGL2 LYZ CYFIP1 ADA2 CST3 HSPA6 RAB24 BR13 CTSZ PLAUR SIGLEC5 OSCAR CD14 PSAP HK3 FCN1 GRN CYSTM1 LILRB2 SERPINA1 PLEKHO2 PRKCD HSPA1B CEACAM1 GSDMD CD300A HLA-C CFD FCGR3A AOC1 STOM
g
Symptomatics
Asymptomatics
IFN-γ signalling
TRIM34 ICAM1 PRKCD SOCS3 IRF1 IRF2 FCGR1BP TRIM25 IFI30 GBP1 PML STAT1 TRIM5 SP100 GBP4 GBP5 FCGR1A GBP2 TRIM21 IRF9 IRF5 TRIM26 IRF7 TRIM14 TRIM22 MT2A OASL OAS1 OAS2 OAS3 GBP6 TRIM6 JAK2 SOCS1 GBP3 TRIM38 HLA-DRB5 HLA-DPA1 HLA-DRB1 HLA-B HLA-C HLA-G HLA-A HLA-H
h
Symptomatics
Asymptomatics
Neuroinflammation
TLR3 CX3CR1 GRIN2D NLRP3 TICAM1 CSF1R FZD1 HLA-DRB5 HMOX1 SLC1A3 NAIP PLA2G4C ICAM1 NCF1 GABRR2 FAS IL1B TLR5 PLA2G4A TNF TICAM2 BMPR2 MR1 HLA-A HLA-G SLC6A12 HLA-B HLA-C HLA-DPA1 HLA-DRB1 CD86 CYBB P2RX7 CXCL10 CCL2 GRIN3A IRF7 JAK2 TLR7 MYD88 CASP1 STAT1
i
IL-1 family signalling
CUL1 PSMA4 MAP2K6 RIPK2 IL1B NOD2 NOD1 CASP1 PSME2 IL1RN MYD88 PSMB8 PSMB9 PSMB10 PSME1
1 2 3 7 10 14 1 2 3 7 10 14 day p.i.
j
TREM1 signalling
TLR3 MPO CASP5 CCL2 NLRC5 CASP1 NOD1 TYROBP MYD88 NOD2 ICAM1 NLRP3 TNF CD86 JAK2 TLR7 FCGR2B TLR4 IL1B TLR5 CD40 NLRC4
1 2 3 7 10 14 1 2 3 7 10 14 day p.i.
log$_2$foldchange
-2 -1 0 1 2 3
k
Dendritic cell maturation
NGFR TLR3 CD40 IL1B FCGR2B FCGR3B TLR4 ICAM1 LTBR TNF FCER1G CD86 FCGR2C IL1RN MYD88 FCGR1A STAT1 STAT2 NFKBI IL15 JAK2 MR1 HLA-DRB5 HLA-DPA1 HLA-DRB1 HLA-A HLA-G TYR HLA-B HLA-C CD1D FCGR3A
1 2 3 7 10 14 1 2 3 7 10 14 day p.i.

Supplementary Fig. 3. Heatmaps of highly DEG-enriched pathways in blood associated with symptomatic and asymptomatic disease. a-k, Heatmap representation of DEGs involved in highly-enriched pathways in the blood of symptomatic (n = 13) and asymptomatic (n = 3) participants post-inoculation. The *P*-values reported are derived from DESeq2 analysis and indicate the statistical significance of differences in gene expression levels (normalised mean counts) between each post-inoculation timepoint and baseline (*$P < 0.05$, **$P < 0.01$, ***$P < 0.001$, ****$P < 0.0001$).

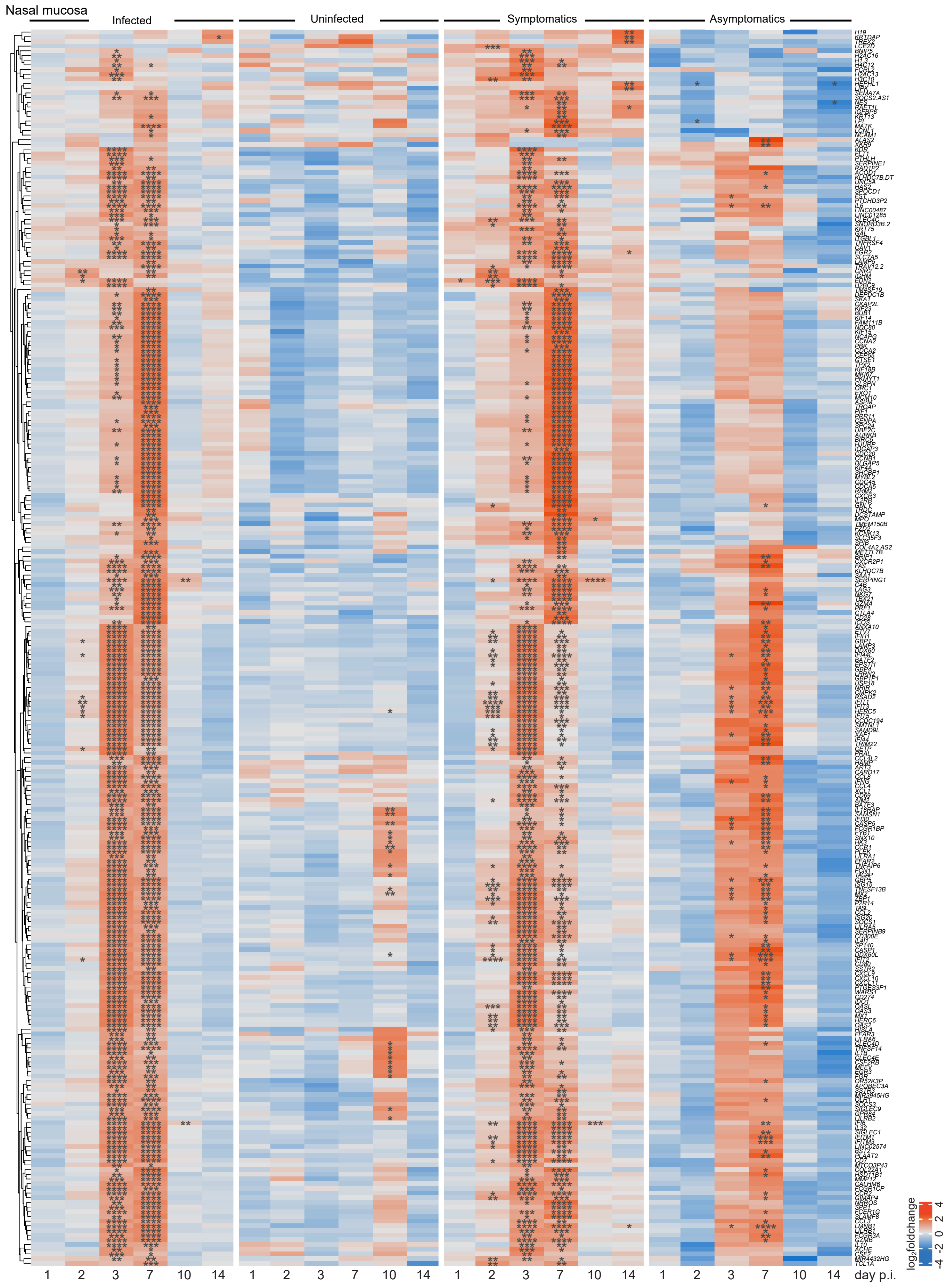
Nasal mucosa
Infected
Uninfected
Symptomatics
Asymptomatics
1 2 3 7 10 14
1 2 3 7 10 14
1 2 3 7 10 14
1 2 3 7 10 14
day p.i.
log2foldchange
-4 -2 0 2 4

Supplementary Fig. 4. Heatmap representation of the top DEGs in the nasal mucosa post-inoculation. High-definition heatmap representation of the top DEGs (log_2 fold-change > 2 and P_{adj} < 0.01) the nasal mucosa (n = 264) post-inoculation. The *P*-values reported are derived from DESeq2 analysis and indicate the statistical significance of differences in gene expression levels (normalised mean counts) between each post-inoculation timepoint and baseline (**P* < 0.05, ***P* < 0.01, ****P* < 0.001, *****P* < 0.0001).

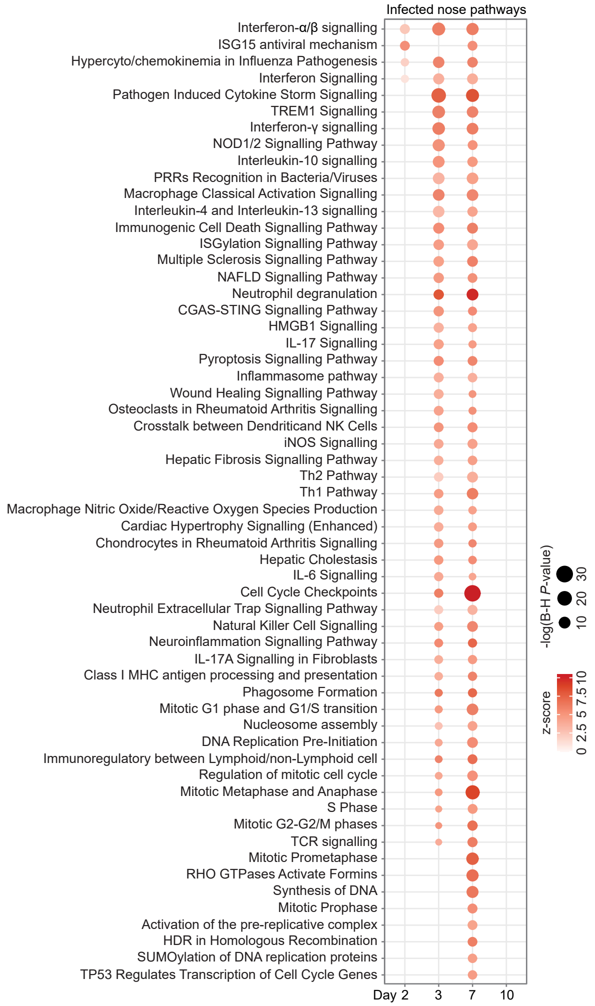

Supplementary Fig. 5. Extended analysis of the most significantly DEG-enriched pathways in the nasal mucosa post-inoculation. The most significantly DEG-enriched pathways (B-H $P_{adj} < 10^{-5}$, z-score > 3.5) in the nasal mucosa of infected (n = 9, symptomatic; n = 4, asymptomatic) participants post-inoculation are shown by IPA. Significance was assessed using a right-tailed Fisher's Exact Test. The *P*-values were adjusted using the Benjamini-Hochberg method for multiple hypothesis test correction.

a Blood

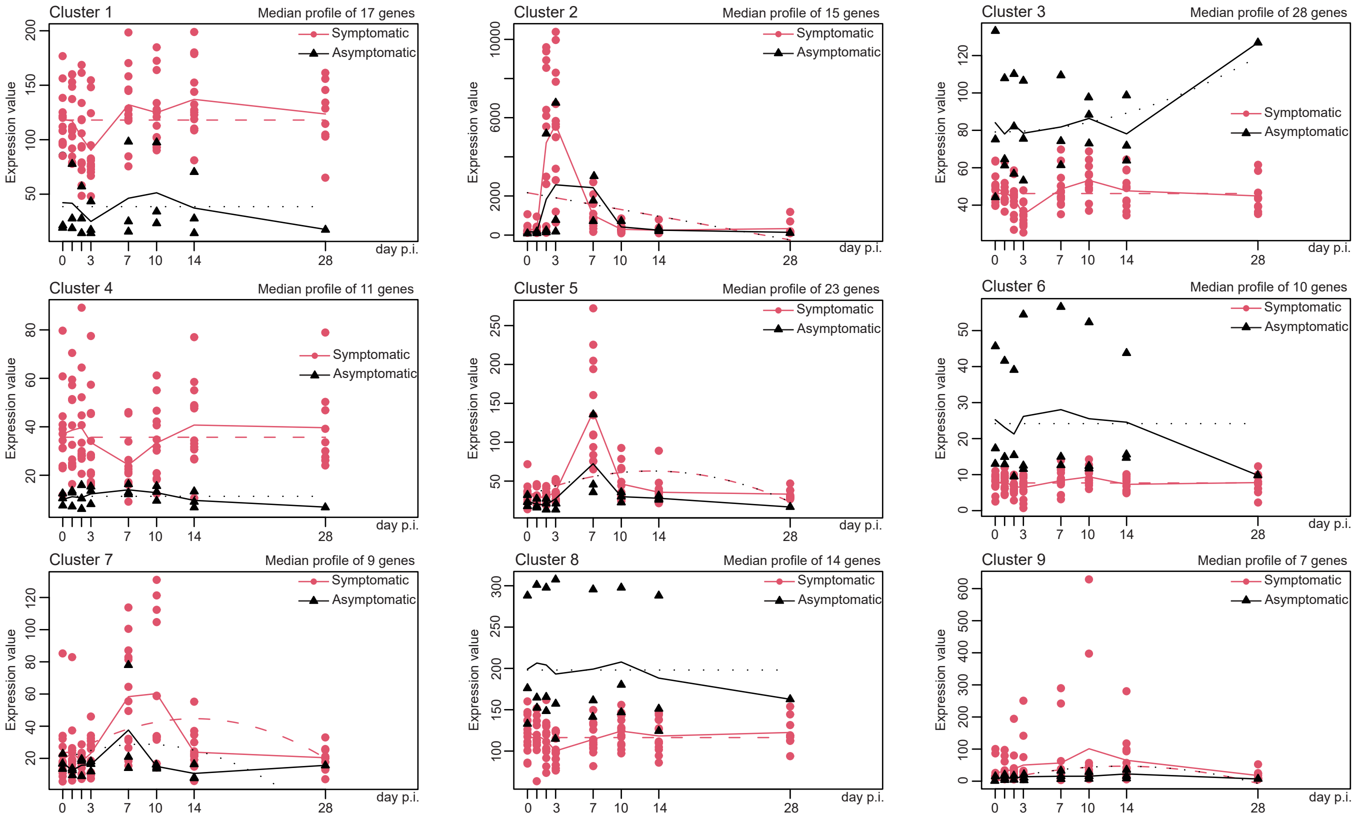

b Nasal mucosa

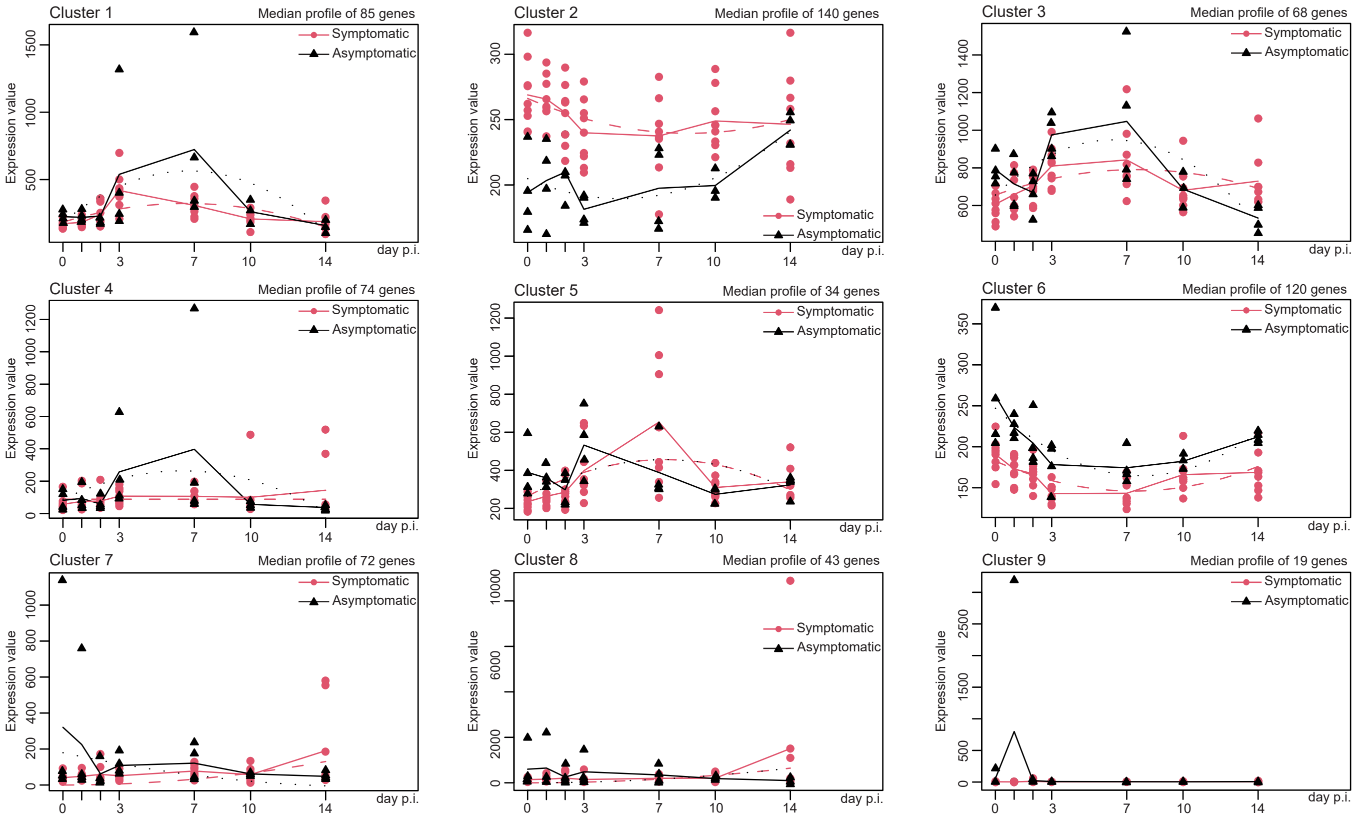

Supplementary Fig. 6. Gene clusters and temporal expression patterns of DEGs identified by maSigPro. a,b, Gene clusters and the temporal expression patterns were identified in the blood (**a**) of n = 13 symptomatic and n = 3 asymptomatic participants, and the nasal mucosa (**b**) of n = 9 symptomatic and n = 4 asymptomatic participants. Solid lines connect the median DEG expression values for each group of participants and the dashed lines show the regression curves fitted to the data.

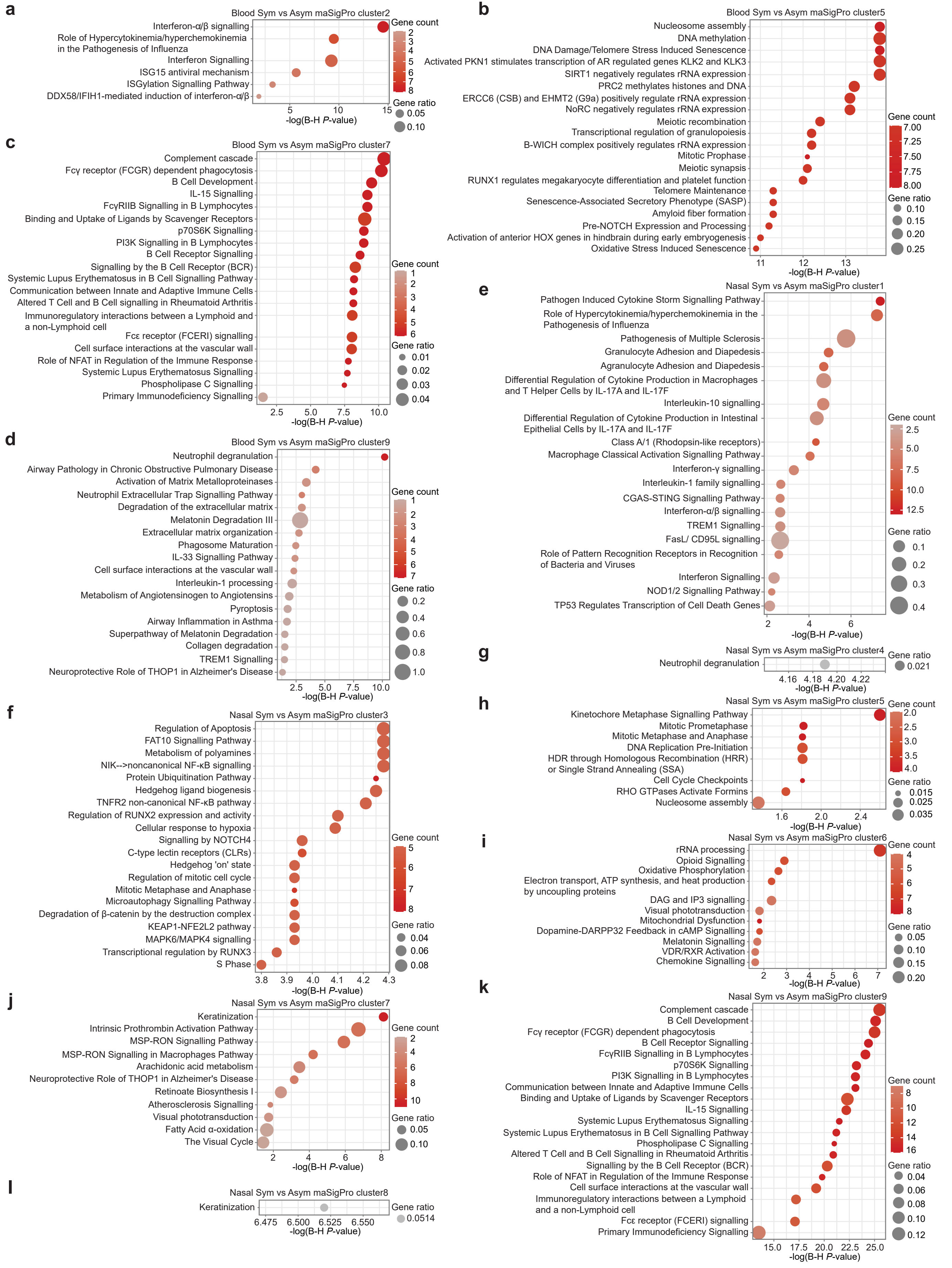
a
Blood Sym vs Asym maSigPro cluster2
Interferon-α/β signalling
Role of Hypercytokinemia/hyperchemokinemia in the Pathogenesis of Influenza
Interferon Signalling
ISG15 antiviral mechanism
ISGylation Signalling Pathway
DDX58/IFIH1-mediated induction of interferon-α/β
5
10
15
-log(B-H P-value)
Gene count
2
3
4
5
6
7
8
Gene ratio
0.05
0.10
b
Blood Sym vs Asym maSigPro cluster5
Nucleosome assembly
DNA methylation
DNA Damage/Telomere Stress Induced Senescence
Activated PKN1 stimulates transcription of AR regulated genes KLK2 and KLK3
SIRT1 negatively regulates rRNA expression
PRC2 methylates histones and DNA
ERCC6 (CSB) and EHMT2 (G9a) positively regulate rRNA expression
NoRC negatively regulates rRNA expression
Meiotic recombination
Transcriptional regulation of granulopoiesis
B-WICH complex positively regulates rRNA expression
Mitotic Prophase
Meiotic synapsis
RUNX1 regulates megakaryocyte differentiation and platelet function
Telomere Maintenance
Senescence-Associated Secretory Phenotype (SASP)
Amyloid fiber formation
Pre-NOTCH Expression and Processing
Activation of anterior HOX genes in hindbrain during early embryogenesis
Oxidative Stress Induced Senescence
11
12
13
-log(B-H P-value)
Gene count
7.00
7.25
7.50
7.75
8.00
Gene ratio
0.10
0.15
0.20
0.25
c
Blood Sym vs Asym maSigPro cluster7
Complement cascade
Fcγ receptor (FCGR) dependent phagocytosis
B Cell Development
IL-15 Signalling
FcγRIIB Signalling in B Lymphocytes
Binding and Uptake of Ligands by Scavenger Receptors
p70S6K Signalling
PI3K Signalling in B Lymphocytes
B Cell Receptor Signalling
Signalling by the B Cell Receptor (BCR)
Systemic Lupus Erythematosus in B Cell Signalling Pathway
Communication between Innate and Adaptive Immune Cells
Altered T Cell and B Cell signalling in Rheumatoid Arthritis
Immunoregulatory interactions between a Lymphoid and a non-Lymphoid cell
Fcε receptor (FCERI) signalling
Cell surface interactions at the vascular wall
Role of NFAT in Regulation of the Immune Response
Systemic Lupus Erythematosus Signalling
Phospholipase C Signalling
Primary Immunodeficiency Signalling
2.5
5.0
7.5
10.0
-log(B-H P-value)
Gene count
1
2
3
4
5
6
Gene ratio
0.01
0.02
0.03
0.04
d
Blood Sym vs Asym maSigPro cluster9
Neutrophil degranulation
Airway Pathology in Chronic Obstructive Pulmonary Disease
Activation of Matrix Metalloproteinases
Neutrophil Extracellular Trap Signalling Pathway
Degradation of the extracellular matrix
Melatonin Degradation III
Extracellular matrix organization
Phagosome Maturation
IL-33 Signalling Pathway
Cell surface interactions at the vascular wall
Interleukin-1 processing
Metabolism of Angiotensinogen to Angiotensins
Pyroptosis
Airway Inflammation in Asthma
Superpathway of Melatonin Degradation
Collagen degradation
TREM1 Signalling
Neuroprotective Role of THOP1 in Alzheimer's Disease
2.5
5.0
7.5
10.0
-log(B-H P-value)
Gene count
1
2
3
4
5
6
7
Gene ratio
0.2
0.4
0.6
0.8
1.0
e
Nasal Sym vs Asym maSigPro cluster1
Pathogen Induced Cytokine Storm Signalling Pathway
Role of Hypercytokinemia/hyperchemokinemia in the Pathogenesis of Influenza
Pathogenesis of Multiple Sclerosis
Granulocyte Adhesion and Diapedesis
Agranulocyte Adhesion and Diapedesis
Differential Regulation of Cytokine Production in Macrophages and T Helper Cells by IL-17A and IL-17F
Interleukin-10 signalling
Differential Regulation of Cytokine Production in Intestinal Epithelial Cells by IL-17A and IL-17F
Class A/1 (Rhodopsin-like receptors)
Macrophage Classical Activation Signalling Pathway
Interferon-γ signalling
Interleukin-1 family signalling
CGAS-STING Signalling Pathway
Interferon-α/β signalling
TREM1 Signalling
FasL/ CD95L signalling
Role of Pattern Recognition Receptors in Recognition of Bacteria and Viruses
Interferon Signalling
NOD1/2 Signalling Pathway
TP53 Regulates Transcription of Cell Death Genes
2
4
6
-log(B-H P-value)
Gene count
2.5
5.0
7.5
10.0
12.5
Gene ratio
0.1
0.2
0.3
0.4
f
Nasal Sym vs Asym maSigPro cluster3
Regulation of Apoptosis
FAT10 Signalling Pathway
Metabolism of polyamines
NIK-->noncanonical NF-κB signalling
Protein Ubiquitination Pathway
Hedgehog ligand biogenesis
TNFR2 non-canonical NF-κB pathway
Regulation of RUNX2 expression and activity
Cellular response to hypoxia
Signalling by NOTCH4
C-type lectin receptors (CLRs)
Hedgehog 'on' state
Regulation of mitotic cell cycle
Mitotic Metaphase and Anaphase
Microautophagy Signalling Pathway
Degradation of β-catenin by the destruction complex
KEAP1-NFE2L2 pathway
MAPK6/MAPK4 signalling
Transcriptional regulation by RUNX3
S Phase
3.8
3.9
4.0
4.1
4.2
4.3
-log(B-H P-value)
Gene count
5
6
7
8
Gene ratio
0.04
0.06
0.08
g
Nasal Sym vs Asym maSigPro cluster4
Neutrophil degranulation
4.16
4.18
4.20
4.22
-log(B-H P-value)
Gene ratio
0.021
h
Nasal Sym vs Asym maSigPro cluster5
Kinetochore Metaphase Signalling Pathway
Mitotic Prometaphase
Mitotic Metaphase and Anaphase
DNA Replication Pre-Initiation
HDR through Homologous Recombination (HRR) or Single Strand Annealing (SSA)
Cell Cycle Checkpoints
RHO GTPases Activate Formins
Nucleosome assembly
1.6
2.0
2.4
-log(B-H P-value)
Gene count
2.0
2.5
3.0
3.5
4.0
Gene ratio
0.015
0.025
0.035
i
Nasal Sym vs Asym maSigPro cluster6
rRNA processing
Opioid Signalling
Oxidative Phosphorylation
Electron transport, ATP synthesis, and heat production by uncoupling proteins
DAG and IP3 signalling
Visual phototransduction
Mitochondrial Dysfunction
Dopamine-DARPP32 Feedback in cAMP Signalling
Melatonin Signalling
VDR/RXR Activation
Chemokine Signalling
2
3
4
5
6
7
-log(B-H P-value)
Gene count
4
5
6
7
8
Gene ratio
0.05
0.10
0.15
0.20
j
Nasal Sym vs Asym maSigPro cluster7
Keratinization
Intrinsic Prothrombin Activation Pathway
MSP-RON Signalling Pathway
MSP-RON Signalling in Macrophages Pathway
Arachidonic acid metabolism
Neuroprotective Role of THOP1 in Alzheimer's Disease
Retinoate Biosynthesis I
Atherosclerosis Signalling
Visual phototransduction
Fatty Acid α-oxidation
The Visual Cycle
2
4
6
8
-log(B-H P-value)
Gene count
2
4
6
8
10
Gene ratio
0.05
0.10
k
Nasal Sym vs Asym maSigPro cluster9
Complement cascade
B Cell Development
Fcγ receptor (FCGR) dependent phagocytosis
B Cell Receptor Signalling
FcγRIIB Signalling in B Lymphocytes
p70S6K Signalling
PI3K Signalling in B Lymphocytes
Communication between Innate and Adaptive Immune Cells
Binding and Uptake of Ligands by Scavenger Receptors
IL-15 Signalling
Systemic Lupus Erythematosus Signalling
Systemic Lupus Erythematosus in B Cell Signalling Pathway
Phospholipase C Signalling
Altered T Cell and B Cell Signalling in Rheumatoid Arthritis
Signalling by the B Cell Receptor (BCR)
Role of NFAT in Regulation of the Immune Response
Cell surface interactions at the vascular wall
Immunoregulatory interactions between a Lymphoid and a non-Lymphoid cell
Fcε receptor (FCERI) signalling
Primary Immunodeficiency Signalling
15.0
17.5
20.0
22.5
25.0
-log(B-H P-value)
Gene count
8
10
12
14
16
Gene ratio
0.04
0.06
0.08
0.10
0.12
l
Nasal Sym vs Asym maSigPro cluster8
Keratinization
6.475
6.500
6.525
6.550
-log(B-H P-value)
Gene ratio
0.0514

Supplementary Fig. 7. maSigPro-clustered DEG-enriched pathways post-inoculation. a-l, Up to 20 most upregulated maSigPro-clustered DEG-enriched pathways in the blood (**a-d**) and the nasal mucosa (**e-l**) are visualised by IPA. Significance was assessed using right-tailed Fisher's Exact Tests. The *P*-values were adjusted using the Benjamini-Hochberg method for multiple hypothesis test correction. Only clusters that resulted in significantly enriched pathways (B-H $P_{adj} < 0.05$) are shown.

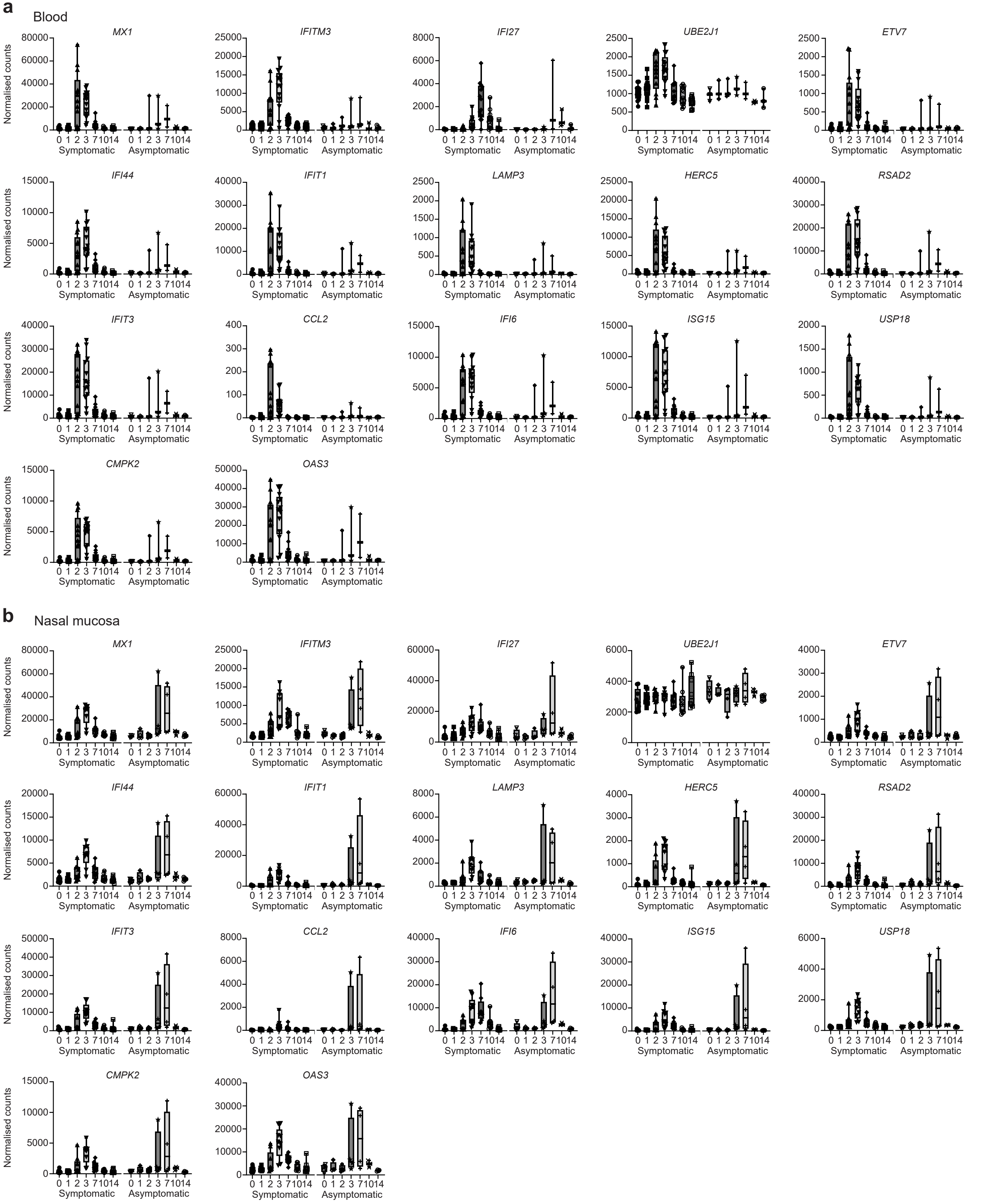

Supplementary Fig. 8. Longitudinal expression levels of canonical ISGs in the blood and the nasal mucosa. a,b, Gene counts of canonical ISGs on baseline (day 0 and day -14 pre-inoculation for blood [**a**] and nasal mucosa [**b**], respectively) and at days 1, 2, 3, 7, 10 and 14 p.i. for symptomatic (n = 13, blood; n = 9, nasal mucosa) and asymptomatic (n = 3, blood; n = 4, nasal mucosa) participants measured as described in Extended Data Fig. 1a. Data are min to max, showing all points.

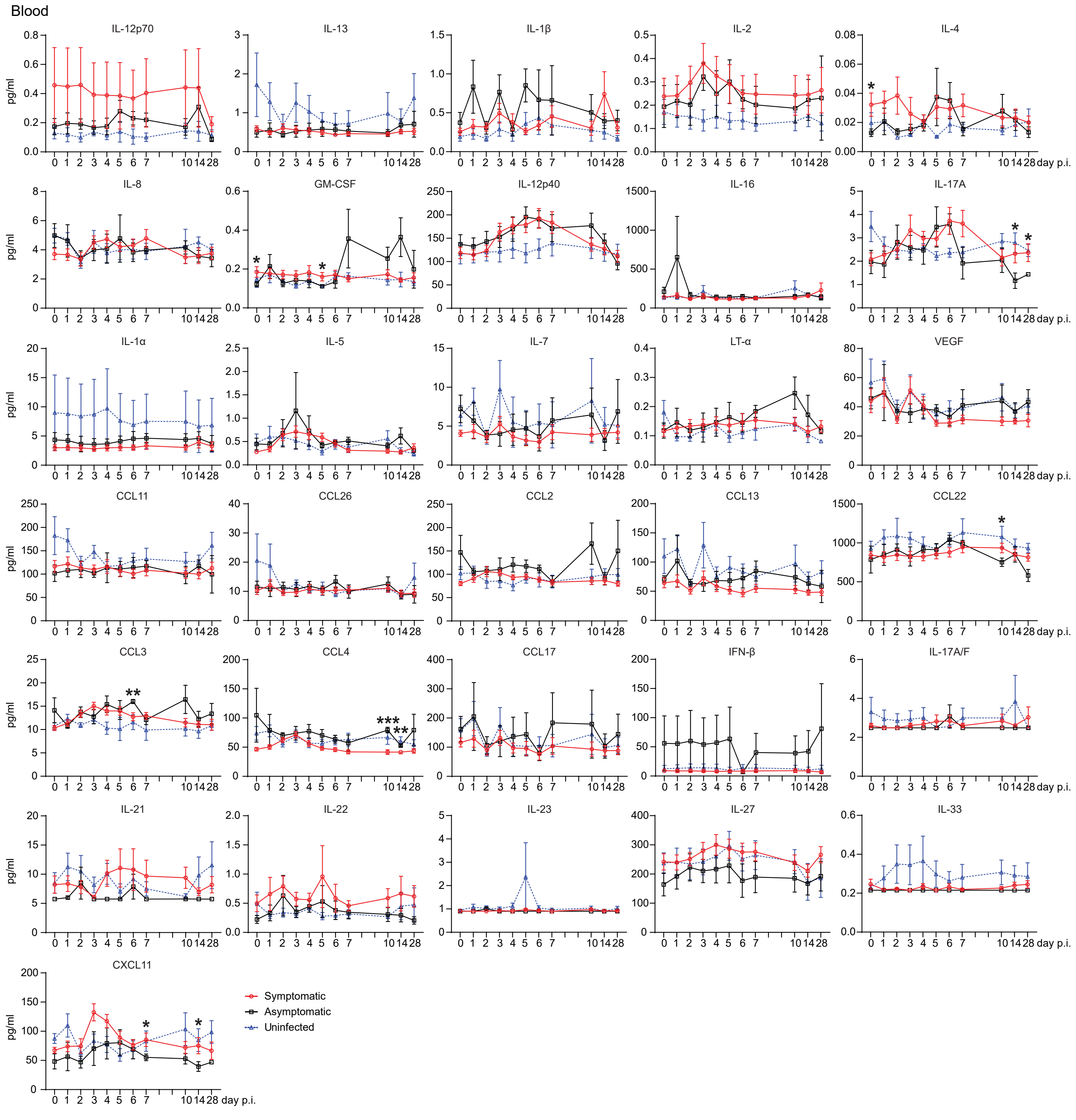

Supplementary Fig. 9. Additional soluble mediator concentrations in plasma. Cytokine and chemokine levels in plasma samples from symptomatic (n = 18), asymptomatic (n = 4) and uninfected (n = 5) participants as measured by Meso Scale Discovery. Data are raw concentrations at pg/ml and results are shown as mean concentrations ± S.E.M., every day from day 0 (pre-inoculation) until day 7 and at days 10, 14 and 28 p.i. Undetectable sample values were given the average lower limit of detection (LLOD) among all plates. Significance between symptomatic and asymptomatic participants was tested by a two-way linear mixed-effects model (REML) with Geisser-Greenhouse correction and post hoc pairwise comparisons were adjusted using the Holm-Šidák method (**P* < 0.05, ***P* < 0.01, ****P* < 0.001, *****P* < 0.0001). No statistical tests are shown for uninfected participants.

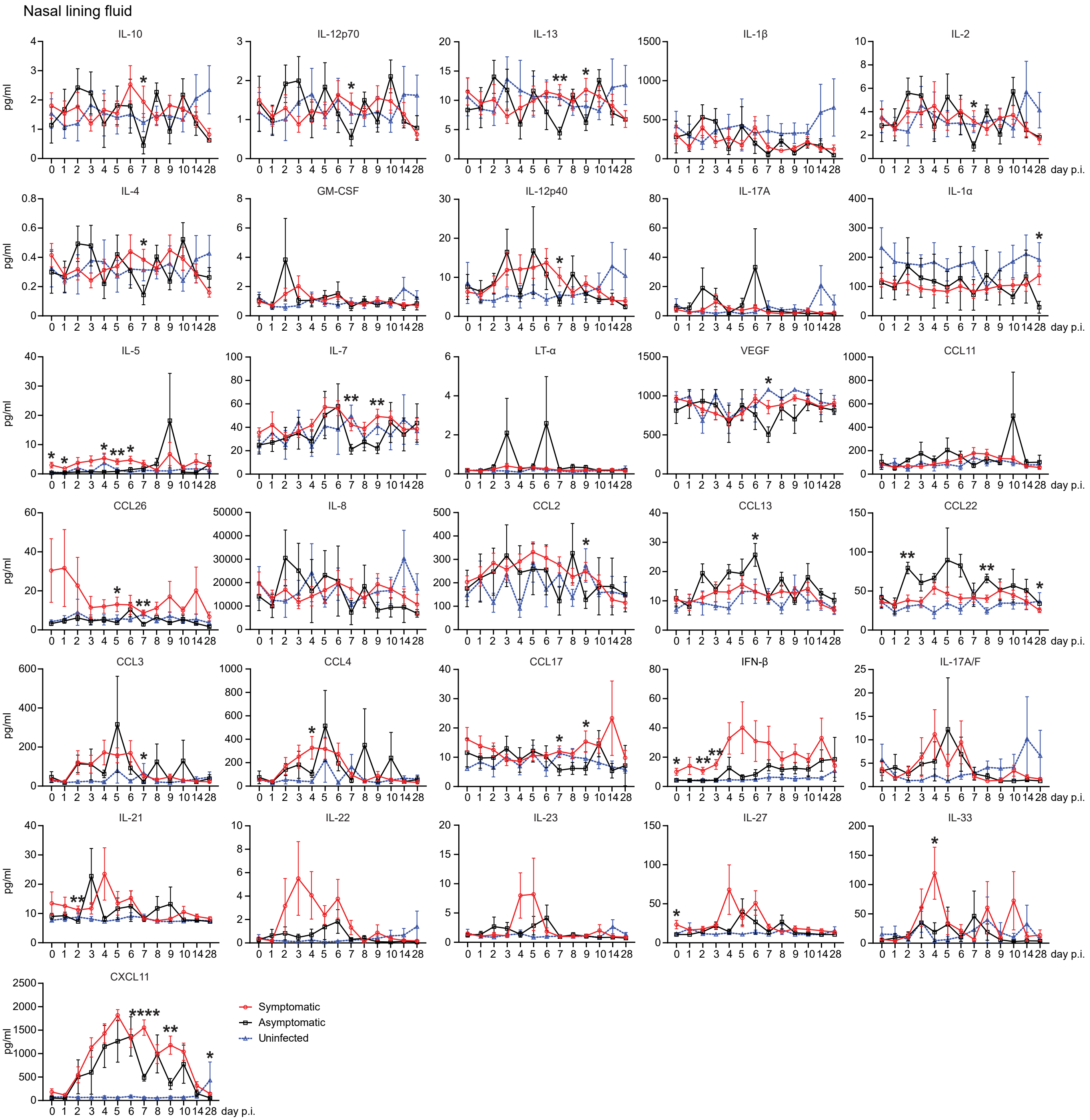

Supplementary Fig. 10. Additional soluble mediator concentrations in nasal lining fluid. Cytokine and chemokine levels in nasosorption samples from symptomatic (n = 18), asymptomatic (n = 4) and uninfected (n = 5) participants as measured by Meso Scale Discovery. Data are raw concentrations at pg/ml and results are shown as mean concentrations ± S.E.M., every day from day 0 (pre-inoculation) until day 10 and at days 14 and 28 p.i. Undetectable sample values were given the average lower limit of detection (LLOD) among all plates. Significance between symptomatic and asymptomatic participants was tested by a two-way linear mixed-effects model (REML) with Geisser-Greenhouse correction and post hoc pairwise comparisons were adjusted using the Holm-Šidák method (**P* < 0.05, ***P* < 0.01, ****P* < 0.001, *****P* < 0.0001). No statistical tests are shown for uninfected participants.

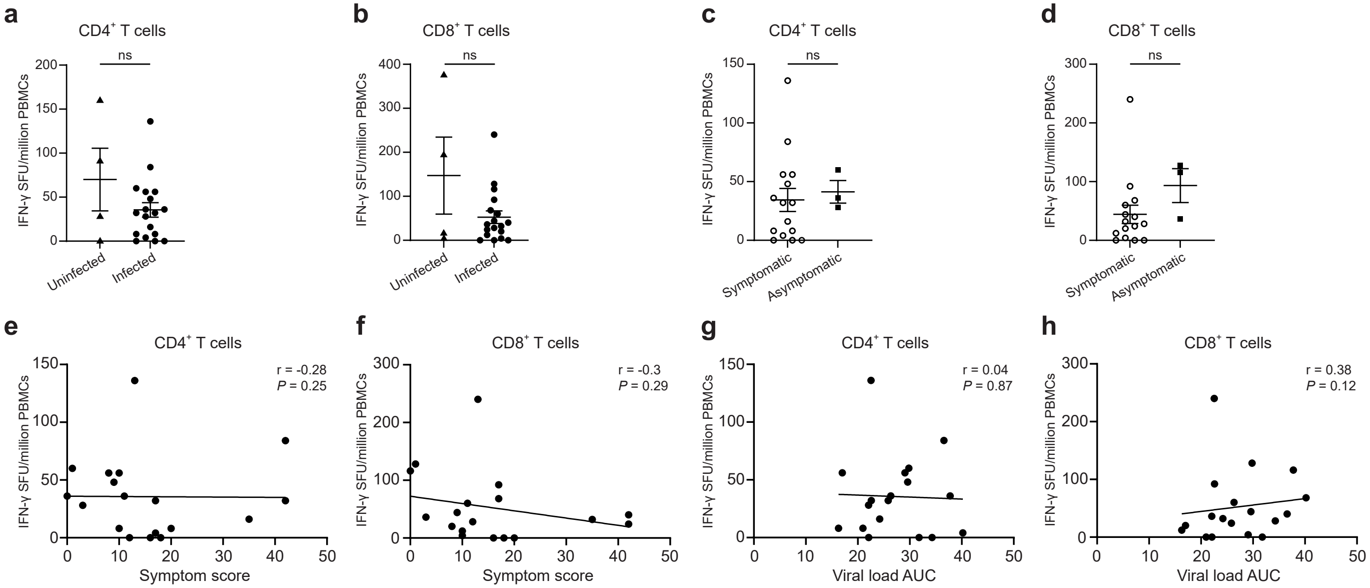

Supplementary Fig. 11. Pre-existing influenza-specific T cell responses. a-d, $CD4^+$ (**a**) and $CD8^+$ (**b**) influenza-specific pre-existing T cell responses for infected (n = 18) and uninfected (n = 4) individuals assessed after Influenza peptide pool stimulation and IFN-γ ELISpot assay, and $CD4^+$ (**c**) and $CD8^+$ (**d**) influenza-specific pre-existing T cell responses for symptomatic (n = 15) and asymptomatic (n = 3) individuals. Influenza-specific T cell numbers are shown as spot-forming units (SFU) per 1 million PBMCs. Data are mean ± S.E.M. Significance was tested by two-sided Mann-Whitney U-tests. **e-f,** Spearman's correlation between symptom score and $CD4^+$ (**e**) or $CD8^+$ (**f**) Influenza-specific pre-existing T cell responses for infected participants (n = 18). **g,h,** Spearman's correlation between viral load area under curve (AUC) and $CD4^+$ (**g**) or $CD8^+$ (**h**) Influenza-specific pre-existing T cell responses for infected participants (n = 18). Significance was assessed using Spearman's rank correlation coefficient (two-sided).
