# Supplementary material for: Innate immune responsiveness predicts enhanced cellular immunity and symptomatic disease after controlled human influenza infection
Source: Nat Med. 2026 Jul 1;32(7):2556–69. doi: 10.1038/s41591-026-04483-7 (PMC13375583; doi:10.1038/s41591-026-04483-7)
Supplement: Supplementary file 2 — Reporting Summary [file 41591_2026_4483_MOESM2_ESM.pdf]

nature portfolio

Corresponding author(s): Christopher Chiu

Last updated by author(s): May 5, 2026

Reporting Summary

Nature Portfolio wishes to improve the reproducibility of the work that we publish. This form provides structure for consistency and transparency in reporting. For further information on Nature Portfolio policies, see our Editorial Policies and the Editorial Policy Checklist.

Statistics

For all statistical analyses, confirm that the following items are present in the figure legend, table legend, main text, or Methods section.

n/a	Confirmed	
☐	☒	The exact sample size (*n*) for each experimental group/condition, given as a discrete number and unit of measurement
☐	☒	A statement on whether measurements were taken from distinct samples or whether the same sample was measured repeatedly
☐	☒	The statistical test(s) used AND whether they are one- or two-sided<br>*Only common tests should be described solely by name; describe more complex techniques in the Methods section.*
☒	☐	A description of all covariates tested
☐	☒	A description of any assumptions or corrections, such as tests of normality and adjustment for multiple comparisons
☐	☒	A full description of the statistical parameters including central tendency (e.g. means) or other basic estimates (e.g. regression coefficient) AND variation (e.g. standard deviation) or associated estimates of uncertainty (e.g. confidence intervals)
☐	☒	For null hypothesis testing, the test statistic (e.g. *F*, *t*, *r*) with confidence intervals, effect sizes, degrees of freedom and *P* value noted<br>*Give P values as exact values whenever suitable.*
☒	☐	For Bayesian analysis, information on the choice of priors and Markov chain Monte Carlo settings
☒	☐	For hierarchical and complex designs, identification of the appropriate level for tests and full reporting of outcomes
☐	☒	Estimates of effect sizes (e.g. Cohen's *d*, Pearson's *r*), indicating how they were calculated

Our web collection on statistics for biologists contains articles on many of the points above.

Software and code

Policy information about availability of computer code

Data collection	Flow cytometry data were collected using BD FACSDiva™ Software v9.0 (FACSymphony A3). Meso Scale Discovery (MSD) multiplex immunoassay data were collected using Meso Scale reader (Meso Scale Diagnostics). Luminex data were collected using Bio-Plex Manager Software 6 (Bio-Plex® 200 system, Bio-Rad). Viral shedding data were collected using AriaMx HRM qPCR Software (Agilent). Transciptional profiling data were collected using NovaSeq Control and HCS 3.4.0 Software. Absorbance (microneutralisation assay) was measured at 450nm using an Omega microplate reader. ELISpot plates were read using an AID vSpot Spectrum Analyser. All other data were collected without software.

Data analysis	Transcriptomics analyses were performed in R (version 4.1.3); annotation with Ensembl gene ID and gene names was performed using the biomaRt Bioconductor package (version 2.50.3); genes were normalised using the DESeq2 package (V1.34.0); principal component analysis (PCA) was performed and visualised using ggplot2 (version 3.3.6); heatmap visualisation was performed using the ComplexHeatmap package (version 2.10.0). Pathway enrichment analysis was performed using Ingenuity Pathway Analysis (IPA) (Qiagen). Longitudinal differential gene expression analyses were conducted using maSigPro package (version 1.66.0). Spearman's correlation tests were visualised using corrplot package (version 0.92). Flow cytometry data were analysed and visualised using GraphPad Prism (version 10.1.1), FlowJo (version 10.10), UMAP_R (v4.0.4 plugin) and FlowSOM (v4.1.0 plugin) software. Soluble mediator data from MSD were analysed using DISCOVERY WORKBENCH 4.0 Analysis Software. Statistical analyses and data visualisation were done using GraphPad Prism (version 10.1.1 to version 10.5.0) and R (version 4.1.3). Diagrams and schematics were created in BioRender. For integrative analyses: Piecewise mixed effects model fitting was performed using MonolixR2019b; temporal dimensionality reduction and interpolation modelling was performed with the MEFISTO framework (https://biofam.github.io/MOFA2/MEFISTO.html) and network-based conditional dependency analysis with the R package GeneNet; conditional temporal dependencies across modalities were inferred using the vector autoregression (VAR) framework and regression coefficients were estimated using LASSO implemented in the glmnet R package (v4.1.8).

For manuscripts utilizing custom algorithms or software that are central to the research but not yet described in published literature, software must be made available to editors and reviewers. We strongly encourage code deposition in a community repository (e.g. GitHub). See the Nature Portfolio guidelines for submitting code & software for further information.

Data

Policy information about availability of data

All manuscripts must include a data availability statement. This statement should provide the following information, where applicable:

- Accession codes, unique identifiers, or web links for publicly available datasets
- A description of any restrictions on data availability
- For clinical datasets or third party data, please ensure that the statement adheres to our policy

Individual participant data that underlie the results reported in this article after deidentification will be made available for individual participant data meta-analysis beginning 12 months and ending 5 years following article publication upon written request. Proposals should be directed to c.chiu@imperial.ac.uk. To gain access, data requestors will need to complete a data request form and sign a data access agreement. Raw viral load, symptom score, soluble mediator, flow cytometry, microneutralisation, ELISpot and any other data will be made available upon reasonable request. In compliance with data privacy restrictions, raw RNA sequencing data from blood samples are under managed access at the European Genome-Phenome Archive (https://ega-archive.org); accession number: EGAD50000000956 (https://ega-archive.org/datasets/EGAD50000000956). Data will be available for investigators whose request will be within the scope of participant consent subject to a data access agreement. RNAseq count data from nasal samples are available in the BioStudies database; accession number E-MTAB-13038 (https://www.ebi.ac.uk/biostudies/). MEFISTO data and codes are available on https://github.com/TruptiAG/MEFISTO. Vector autoregression (VAR) framework codes are available on https://github.com/ah2426/h3n2-challenge.

Research involving human participants, their data, or biological material

Policy information about studies with human participants or human data. See also policy information about sex, gender (identity/presentation), and sexual orientation and race, ethnicity and racism.

Reporting on sex and gender	The study design was aiming to a balanced male and female representation. Out of 27 individuals included in this study 13 were female. Sex was determined based on self-reporting. Principal component analysis on gene expression patterns didn't show any differences. Symptom scores were not significantly different between females and males. The main aim of this study was then focused on the comparison of immune responses in symptomatic and asymptomatic infected participants and no further analyses on sex or gender were performed. Results apply to all sexes.
Reporting on race, ethnicity, or other socially relevant groupings	Race and ethnicity information was based on self-reporting. The study design paid attention to include eligible individuals of any race, ethnicity or other socially relevant groupings, based on age, health and immunological parameters. Principal component analysis on gene expression patterns didn't show any race or ethnicity-related differences. No further analyses were performed based on race or ethnicity information.
Population characteristics	Healthy adult volunteers, aged 18 to 55 years with no known risk factors and low serum neutralising antibody levels against the challenge strain (microneutralisation titre ≤ 1:20) were included in the study.
Recruitment	Recruitment was mainly done via mailshots. Participants who had consented to be contacted again from previous communications were informed about the study, and a database mailshot was sent out. All participants initially contacted the study team via email or telephone. They were provided with the Participant Information Sheet (PIS) and given opportunity to ask questions, while the study team asked them eligibility questions (age, smoking history, health issues, regular medication, allergies, influenza vaccination history, contact with children or elderly persons at home/at work, recent colds, etc.). If all answers were answered satisfactorily, the participants were invited to the Imperial Clinical Research Facility (ICRF) clinic for informed consent and were pre-screened to ensure they are not already immune to the challenge virus. Serum collected and tested for neutralising antibodies against the challenge strain. If the neutralising antibody levels were 1:20 or less, participants was invited to the full safety screening visit by the study team and a consent form was signed in the presence of the study doctor. Past medical history was obtained from the participants GP and was reviewed to ensure suitability for the study.<br>The participant recruitment was biased by the eligibility criteria and self-selection as participation was voluntary. Participants in the study had their travel costs refunded. They were also given a donation of up to £3000 to compensate for the time and inconvenience of taking part in the study.
Ethics oversight	The study was approved by the UK Health Research Authority (London - Fulham Research Ethics Committee, references 11/LO/1826 and 19/LO/1441).

Note that full information on the approval of the study protocol must also be provided in the manuscript.

Field-specific reporting

Please select the one below that is the best fit for your research. If you are not sure, read the appropriate sections before making your selection.

☒ Life sciences ☐ Behavioural & social sciences ☐ Ecological, evolutionary & environmental sciences

For a reference copy of the document with all sections, see nature.com/documents/nr-reporting-summary-flat.pdf

Life sciences study design

All studies must disclose on these points even when the disclosure is negative.

Sample size	A total of 30 study participants were enrolled in two consecutive studies under two protocols with identical study conduct, procedures for influenza challenge and sampling. The sample size calculation was based on the previously established attack rate of 75% using this challenge virus allowing the study of immune responses and symptomatology in infected and uninfected individuals.
Data exclusions	Data from two individuals who were found to be coinfected by rhinovirus, and one who seroconverted in the interval period between screening and inoculation were excluded from all analyses.
Replication	The findings were replicated through the separate cohorts of participants over the course of the two consecutive studies.
Randomization	Study participants were enrolled into the study chronologically based on the recruitment procedures and the capacity of the Imperial Clinical Research Facility (ICRF) clinic to host cohorts of participants in quarantine.
Blinding	As stated in previous work from the study team: 'Study participants and participant-facing staff conducting the clinical study were blinded to the infection status where possible until protocol defined procedures made this apparent. Study participants and clinical staff could not be blinded to the clinical data and symptom scores as they were being generated. Laboratory staff processing the samples were blinded to infection status. Laboratory staff performing assays were not blinded to infection status.'

Reporting for specific materials, systems and methods

We require information from authors about some types of materials, experimental systems and methods used in many studies. Here, indicate whether each material, system or method listed is relevant to your study. If you are not sure if a list item applies to your research, read the appropriate section before selecting a response.

Materials & experimental systems

n/a	Involved in the study	
☐	☒	Antibodies
☐	☒	Eukaryotic cell lines
☒	☐	Palaeontology and archaeology
☒	☐	Animals and other organisms
☒	☐	Clinical data
☒	☐	Dual use research of concern
☒	☐	Plants

Methods

n/a	Involved in the study	
☒	☐	ChIP-seq
☐	☒	Flow cytometry
☒	☐	MRI-based neuroimaging

Antibodies

Antibodies used

Antibody / Fluorochrome / Clone / Species / Supplier name / Catalogue number / Lot number / Dilution
Innate cell panel:
CD86 FITC BU63 Mouse BioLegend 374204 B369732 2:50
CD16 Super Bright 436 3G8 Mouse eBioscience 62-0166-42 2480273 1:50
CD3 (dump) BV510 UCHT1 Mouse BioLegend 300448 B406345 1:50
CD19 (dump) BV510 SJ25C1 Mouse BD Biosciences 562947 2297689 2:50
CD56 (dump) BV510 5.1H11 Mouse BioLegend 362534 B350903 2:50
CCR2 BV605 K036C2 Mouse BioLegend 357214 B332679 2:50
CD11c BV650 3.9 Mouse BioLegend 301637 B325082 2:50
CD40 BV711 5C3 Mouse BioLegend 334334 B358820 2:50
CD141 BV785 M80 Mouse BioLegend 344116 B370735 2:50
CD123 APC 6H6 Mouse BioLegend 306012 B384458 2:50
CD14 APC-H7 MφP9 Mouse BD Biosciences 560180 3009220 2:50
CD1c APC-R700 F10/21A3 Mouse BD Biosciences 566614 3041760 2:50
HLA-ABC PE W6/32 Mouse BioLegend 311406 B381210 0.5:50
HLA-DR PE-CF594 G46-6 Mouse BD Biosciences 562304 1165220 0.5:50
CD169 PE-Cyanine7 7-239 Mouse BioLegend 346014 B354806 2:50

CD11b PerCP/Cyanine5.5 M1/70 Rat BioLegend 101228 B353753 1:50
Viability BV510 N/A N/A Invitrogen L34965 2456951 1:500
NK cell panel:
CD57 eFluor450 TB01 Mouse eBioscience 48-0577-42 2437632 1:20
CD3 BV510 UCHT1 Mouse BioLegend 300448 B369160 1:20
CD16 BV605 3G8 Mouse BioLegend 302039 B367756 0.5:20
NKG2C PE 134591 Mouse R&D Systems FAB138P LCN0821031 1:20
CD38 PE-CF594 HIT2 Mouse BD Biosciences 562288 2353921 0.25:20
CD56 (dump) PE-Cyanine7 NCAM16.2 Mouse BD Biosciences 335826 2276813 0.5:20
Ki-67 BV786 B56 Mouse BD Biosciences 563756 2263445 1:20
Viability UV515 N/A N/A BioLegend 423108 B358473 1:250
T cell panel:
CD8 PerCP/Cyanine5.5 RPA-T8 Mouse eBioscience 45-0088-42 2446952 1:84
CXCR3 BV421 1C6/CXCR3 Mouse BD Biosciences 562558 3012444 5:84
CD3 BV510 UCHT1 Mouse BioLegend 300448 B406345 1:84
CD69 BV711 FN50 Mouse BioLegend 310944 B391063 5:84
CD49d BV786 L25 Mouse BD Biosciences 744751 4120718 5:84
CD4 APC-H7 SK3 Mouse BD Biosciences 641398 3313161 2:84
CD11a Alexa Fluor 700 HI111 Mouse BioLegend 301228 B332663 5:84
CD38 PE-CF594 HIT2 Mouse BD Biosciences 562288 2353921 2:84
Ki-67 FITC B56 Mouse BD Biosciences 556026 1130847 5:10
Viability UV515 N/A N/A BioLegend 423108 B358473 1:250
PBMC Stimulation ICS-including panel:
CD14 APC-H7 MϕP9 Mouse BD Biosciences 560180 3009220 2:50
CD3 BUV805 UCHT1 Mouse BD Biosciences 612896 3311042 1:50
CD11c BV650 3.9 Mouse BioLegend 301638 B353469 2:50
CD16 Super Bright 436 3G8 Mouse eBioscience 62-0166-42 2480273 1:50
CD19 BV605 HIB19 Mouse BioLegend 302244 B402148 0.5:50
CD56 BUV615 NCAM16.2 Mouse BD Biosciences 613001 3191263 1:50
CD123 BV711 9F5 Mouse BD Biosciences 563161 3013203 2:50
HLA-DR R718 G46-6 Mouse BD Biosciences 567035 3283141 1:50
CD8 PerCP/Cyanine5.5 RPA-T8 Mouse eBioscience 45-0088-42 2446952 1:50
CD4 BUV496 SK3 Mouse BD Biosciences 612936 3199162 1:50
IL-10 APC JES3-19F1 Rat BioLegend 506807 B370178 2:40
IFNγ BV785 4S.B3 Mouse BioLegend 502542 B374171 2:40
IL-1β PE 8516 Mouse R&D Systems IC201P LHV0720121 4:40
TNF PE-Cyanine7 MAb11 Mouse eBioscience 25-7349-82 2698074 0.5:40
IL-6 FITC MQ2-13A5 Rat BioLegend 501104 B395110 2:40
Viability BV510 N/A N/A Invitrogen L34965 2456951 1:500

All primary antibodies used were commercially sourced and validated for flow cytometry on human cells by the manufacturers stated above.

Validation

All used antibodies were commercially purchased.

Eukaryotic cell lines

Policy information about cell lines and Sex and Gender in Research

Cell line source(s)

MDCK-SIAT1 cells (source: kidney of an adult female Cocker Spaniel [Madin-Darby Canine Kidney], cellular sub-clone that has been genetically engineered to overexpress the human 2,6-sialyltransferase [SIAT1] enzyme)

Authentication

MDCK-SIAT1 cells were not authenticated.

Mycoplasma contamination

MDCK-SIAT1 cells were tested negative for mycoplasma contamination.

Commonly misidentified lines
(See ICLAC register)

N/A

Plants

Seed stocks

Report on the source of all seed stocks or other plant material used. If applicable, state the seed stock centre and catalogue number. If plant specimens were collected from the field, describe the collection location, date and sampling procedures.

Novel plant genotypes

Describe the methods by which all novel plant genotypes were produced. This includes those generated by transgenic approaches, gene editing, chemical/radiation-based mutagenesis and hybridization. For transgenic lines, describe the transformation method, the number of independent lines analyzed and the generation upon which experiments were performed. For gene-edited lines, describe the editor used, the endogenous sequence targeted for editing, the targeting guide RNA sequence (if applicable) and how the editor was applied.

Authentication

Describe any authentication procedures for each seed stock used or novel genotype generated. Describe any experiments used to assess the effect of a mutation and, where applicable, how potential secondary effects (e.g. second site T-DNA insertions, mosiacism, off-target gene editing) were examined.

Flow Cytometry

Plots

Confirm that:

☒ The axis labels state the marker and fluorochrome used (e.g. CD4-FITC).

☒ The axis scales are clearly visible. Include numbers along axes only for bottom left plot of group (a 'group' is an analysis of identical markers).

☒ All plots are contour plots with outliers or pseudocolor plots.

☒ A numerical value for number of cells or percentage (with statistics) is provided.

Methodology

Sample preparation	Cryopreserved PBMC were thawed in warm RPMI 1640 (Sigma-Aldrich), washed, and counted using Countess II cell counter (ThermoFisher). Up to 10^6 PBMCs per condition were stained with LIVE/DEAD Fix Aqua Kit (Invitrogen) or Zombie UV Fixable Viability Kit (BioLegend) for 20min at room temperature followed by blockade of nonspecific binding using FcBlock (Miltenyi Biotec) for 10min and then incubated with antibodies against surface markers in FACS buffer (PBS supplemented with 2% FBS and 2mM EDTA) at 4oC for 30min. Following washing with FACS buffer, cells were either fixed with BD CellFIX (BD) or fixed and permeabilized for intracellular staining using Foxp3/Transcription Factor Staining Buffer Set (ThermoFisher).
Instrument	FACSymphony A3
Software	Data collection: BD FACSDiva™ Software v9.0; data analysis: FlowJo (version 10.10), UMAP_R plugin software and GraphPad Prism (version 10.1.1).
Cell population abundance	No cell sorting was performed.
Gating strategy	Gating strategies are described and shown in the Extended Data Fig. 6.<br>Gating strategy for PBMC subpopulation phenotyping was as follows: Live monocytes have been assigned as CD14+/dim excluding other lineage positive and CD14dimCD16- cells and have been separated into three subgroups: CD14+CD16- classical monocytes (CMs), CD14+CD16+ intermediate monocytes (IMs) and CD14dimCD16+ non-classical monocytes (NCMs). Live DCs have been assigned as CD14-HLA-DR+ after excluding other lineage positive cells. DCs were then separated into: CD11c+CD141+ conventional DC1 (cDC1s), CD11c+CD141-CD1c+ conventional DC2s (cDC2s), CD11c+CD141-CD1c- DCs and CD123+ plasmacytoid DCs (pDCs). Live NK cells were assigned as CD56+ and have been separated into CD56bright and CD56dim. Ki-67+ CD56bright and CD56dim NK cells have been assessed as percentage of total CD56bright and CD56dim NK cells, respectively. Live T cells were assigned as CD3+ and have been separated into CD4+ and CD8+ T cells. CD69+ and CD38 +Ki-67+ double positive and CD4+ and CD8+ T cells were assessed as percentage of total CD4+ and CD8+ T cells, respectively.<br><br>Gating strategy for analysis of intracellular production of cytokines in PBMCs was as follows: Live T cells were assigned as CD3 + and have been separated into CD4+ and CD8+ T cells. Live NK cells were assigned as CD3-CD56+ and live B cells as CD3-CD19+. Live monocytes have been assigned as CD14+/dim excluding other lineage positive and CD14dimCD16- cells and have been separated into three subgroups: CD14+CD16- classical monocytes (CMs), CD14+CD16+ intermediate monocytes (IMs) and CD14dimCD16+ non-classical monocytes (NCMs). Live DCs have been assigned as CD14-HLA-DR+ after excluding other lineage positive cells and separated into two populations: CD123+ plasmacytoid DCs (pDCs) and CD11c+CD123- conventional DC (cDCs).

☒ Tick this box to confirm that a figure exemplifying the gating strategy is provided in the Supplementary Information.
