# Supplementary material for: Innate immune responsiveness predicts enhanced cellular immunity and symptomatic disease after controlled human influenza infection
Source: Nat Med. 2026 Jul 1;32(7):2556–69. doi: 10.1038/s41591-026-04483-7 (PMC13375583; doi:10.1038/s41591-026-04483-7)
Supplement: Supplementary file 3 — Peer Review File [file 41591_2026_4483_MOESM3_ESM.pdf]

nature portfolio

Peer Review File

Innate immune responsiveness predicts enhanced cellular immunity and symptomatic disease after controlled human influenza infection

Corresponding Author: Professor Christopher Chiu

This file contains all reviewer reports in order by version, followed by all author rebuttals in order by version.

Version 0:

Reviewer comments:

Reviewer #1

(Remarks to the Author)
In this paper by Papargyris and colleagues, the authors evaluate immunologic predictors of disease outcomes among individuals undergoing controlled human influenza infection. They performed a multimodal analyses, leveraging longitudinal transcriptomics, proteomics, cell phenotyping by flow cytometry, and in vitro stimulation experiments. Among 27 individuals challenged, 22 individuals developed PCR-confirmed infection, and of those, 18 had symptomatic infection and 4 had asymptomatic infection. Days post-inoculation of peak viral and peak symptoms were similar in symptomatic individuals (~day 3-5 post-inoculation). Interestingly, differences in symptom severity were not clearly explained by differences in viral load during the acute phase of infection, but there were trends to more rapid viral clearance in symptomatic vs. asymptomatic individuals. Prior to inoculation, there were minimal differences in whole blood gene expression between clinical outcome groups (infected vs uninfected; symptomatic vs. asymptomatic); although there were more differences in nasal swab gene expression. Following inoculation, there were significant differences in whole blood and nasal swab gene expression in infected vs,. uninfected individuals, and moreso in symptomatic vs. asymptomatically infected individuals. The authors performed longitudinal trajectory modeling of DEGs using maSigPro, finding multiple clusters of differentially expressed genes with distinct temporal responses to infection. These data implicated an early peak in ISGs around Day 3 in symptomatics, cell cycle/proliferation at Day 7, and complement cascade, Fcy receptor dependent phagpocytosis, and B cell pathways at Day 10 – generally higher in symptomatics vs. asymptomatics. Plasma and nasal fluid proteomics generally paralleled these findings, and several markers (Type 1 and 2 IFNs, IL-6, peaked in blood prior to the peak onset of symptoms. Interestingly, asymptomatic infections were associated with significantly higher IL-10, CCL22, and CCl13. IL-10 in blood, in contrast, was higher in symptomatics. When analyzing cell phenotypes by flow cytometry, the authors identified changes in monocyte populations (namely, intermediate monocytes and HLA-expressing monocytes/DCs, along with NKs/CD8s) between symptomatics and asymptomatics. Finally, the authors tested the hypothesis that differences in pre-infection innate cell responsivity might be predictive of symptomatic disease, They cultured PBMC obtained prior to inoculation with heat-inactived virus, and found that levels of IL1B measured in supernatants were higher in symptomatics vs. asymptomatics. They perform additional experiments and further show that responses to in vitro stimulation were lower at Day 14 than prior to infection, suggesting some “refractoriness” of PBMC to restimulation.

Overall, I found the manuscript easy to read and to follow, with intriguing results implicating innate immune activation early following controlled human influenza infection to be associated with symptomatic disease.

The main limitation as acknowledged by the authors is the limited number of participants, particularly among the asymptomatic group (n=4). There isn’t too much the authors can do about this limitation.

What is missing in my opinion is an integrative analysis across modalities. The authors could consider MEFISTO (PMID 35027765) or Bayesian approaches (PMID 38146838). By integrating the multiple related datasets, this might provide additional insight into relationships between genes/proteins/cells beyond individual factor analysis. For example, what might be driving differential IL-10 protein levels? Could such an approach help explain the contradictory results between IL-10 measured in the nasal fluid and IL-10 measured in plasma? Could this help explain why Day 14 responses to stimulation were lower than pre-infection responses? I think this would significantly improve the manuscript and interpretability for readers.

In addition, although the authors analyzed comparisons between symptomatic and asymptomatic individuals, did the authors look to see whether immune responses influenced other outcomes? Rates of viral clearance? Post-infection levels of neutralizing antibodies?

Line 206-208/Figure 5a/Supplementary Figure 6: Was the flow cytometry performed with overlapping panels? If so would share those panels and better describe in the methods how this was performed. I find it curious that the authors only show the UMAP data for monocytes/DCs; would be interested to see data for different cell subsets as well. And, did the authors perform any clustering of cell phenotype data using unsupervised approaches?

Minor: Line 106: In contrast, following infection…. Suggest changing following infection to "among infected individuals"

Reviewer #2

(Remarks to the Author)
In this work, Papagyris et al present immunological results from a controlled human infection studies with influenza A (H3N2) with detailed sampling of blood and nasal samples prior to and post-infection. The frequent sampling allows for temporal analysis of immune cell populations and underlies their main finding that innate immune activation (of monocytes and DCs) precedes T and NK cell activation, and that innate immune activation was associated with the development of symptoms in those who became infected.

The major strength of the work comes from the clinical trial design, with longitudinal sampling pre- and post-infection and analysis of immune parameters at multiple timepoints. This has allowed for the authors to define in detail how the immune response has developed over time. In particular, the availability of pre-infection samples to profile innate immune responsiveness represents a valuable addition to the literature. This is study is elegant, sound and of a great importance for defining the link between the early innate immunity and symptomatic influenza virus infection as well as differential immunity in asymptomatic versus symptomatic individuals.

Data and methodology
-The definition of symptomatic infection was "…total adjusted Jackson symptom score of 6 or more over the first 6 days post-inoculation and reporting having had a cold/flu or reporting nasal discharge on 3 or more consecutive post-inoculation days". The Jackson score is a validated tool that encompasses all the symptoms usually associated with having a "cold/flu" including nasal discharge – so it is unclear why the additional definition was needed. Can the authors justify the use of this composite definition for symptomatic infection?

Interpretation
-One of the key conclusions made by the authors is that pre-existing innate immune responsiveness was associated with the subsequent development of symptoms. Individuals were selected for inclusion on the basis of an absence of neutralising antibodies against the infecting strain, but there was no assessment of prior T cell responses. Cross-reactive T cell responses have been associated with protection against symptoms in infection with both seasonal and pandemic influenza strains (e.g. Sridhar et al 2013 Nat Med DOI: 10.1038/nm.3350, Wilkinson et al 2012 Nat Med DOI: 10.1038/nm.2612, Hayward et al 2015 DOI: 10.1164/rccm.201411-1988OC). An assessment of influenza-specific T cell responses prior to infection is needed and should therefore be considered as part of this work.

-The authors describe their immunological findings as "correlates of severity" (e.g. line 241) and alternate using the terms "disease severity" and "symptom severity" to describe findings. This study involves young people with mild influenza infection (no hospitalisation or oxygen requirements) and these immunological findings may not be the same pathways involved in severe disease. The authors should be consistent about describing their results as reflecting symptom severity in mild disease. Furthermore, in the limitation of the study section, it would be important to mention that the early innate immune response might differ in patients with life-threatening influenza outcomes.

-The conclusion of the manuscript does not reflect the content of the work well. The manuscript describes a nasal inoculation challenge model, but the conclusion focuses around vaccine responses / reactogenicity – it does not necessarily follow that these would be the same. Consider rephrasing this conclusion to reflect the key findings.

Analytical approach
The longitudinal approaches to analyses of all immune parameters measured, in particular the transcriptomic data presented posed a challenge. The authors describe a number of comparisons in the text (nasal versus blood, infected versus uninfected, symptomatic versus asymptomatic). Overall, the presentation and interpretation of the transcriptomic data is not easy to navigate and should be improved to maximise clarity. In particular:
-In Figure 2b and Figure 2d the axes have been condensed so that the gene names are distorted and too small to read. These should either be removed (if the overall goal is to see the clusters only) or expanded to be legible.

-The data in Figures 2f, 2g, 2h, 2i and 2j show differentially expressed genes in symptomatics and asymptomatics at all time points based on selected pathways from pathway analysis (shown in Figure 2c). By selecting genes for inclusion in each plot based on pathway analysis, there is overlap of the genes shown such that several genes are represented in multiple of plots 2f-j (e.g. SOCS3, IRF7, HLA-B-C, C5) as well as in Figure 2b. Figures 2f-j add little value over 2b and 2c, and make the overall interpretation of the data in Figure 2 difficult. Consider removing these.

-The authors should consider formally showing a two-way comparison of the most relevant data to the narrative. Much of the narrative is around differences between gene expression in blood between symptomatics and asymptomatics but this comparison is not formally shown (with all differentially induced genes shown relative to baseline). Consider including this comparison for a timepoint of interest (for example a volcano plot of differentially expressed genes between asymptomatics and symptomatics at Day 2 or Day 3).

-The transcriptomic data have been analysed using maSigPro to identify temporal trends over time, resulting in the identification of gene clusters. In the description of the analysis this describes that this identifies differentially expressed genes “between groups and over time”. Are the differentially expressed genes shown in Figure 3 relative to baseline (i.e. each timepoint relative to pre-inoculation sample) or between the two groups?

-In describing the results in Figure 3a-b the authors compare the lines between symptomatics and asymptomatics (e.g. line 143 states “lower but more prolonged response for asymptomatics”). It does not appear that the conclusion that the response in Figure 3a is more prolonged in asymptomatics is valid based on data presented. Do these trajectories have a statistical test to indicate they are different between groups?

Minor points:
-The term PBMC is used in the abstract without definition – an alternative term or definition should be included here.

-Similarly, in first use in the manuscript (line 283) the term PBMCs is not defined, but it is later defined in the methods – this definition should be moved to the first use.

-The manuscript is lacking key demographic details on participants in each group (sex, age) which are important for generalisability and interpretation of findings. These should be included for each group analysed. Some of this information (sex only) is contained in the reporting summary but should be moved to the manuscript.

-In the ethics statement the term “ICH” should be defined.

-In description of flow cytometry data in line 270 the authors describe “flatter but more prolonged patterns of T cell activation”. This appears to relate to Extended Data Fig 9e where there is no difference between these groups at later time points and it does not appear that it is valid to suggest the response is more prolonged.

-In the results presenting data from flow cytometry experiments there is variation in the number of samples presented (e.g. Figure 5 and 6 describe sample numbers “up to 18” or “up to 4”) but it is not described in the Methods why the sample number varies. What were the inclusion / exclusion criteria for a sample to be part of the results presented?

-For the cytokine stimulation data in Figure 7c the authors comment that the number of individuals included (14) did not allow for comparison between symptomatic and asymptomatic patients (line 294-295). Subsequently Figure 7e compares symptomatic and asymptomatic individuals with either 14 (or fewer) samples included at each timepoint. How were decisions made regarding data inclusion here?

-In the Methods the quantification of influenza RNA based on M gene presence is described (lines 572-574) but the primer sequences are not provided. Please include further detail on how this qPCR was performed.

-In the representative gating (Extended data Figure 6) there are no numbers (percentages) on the gates shown; in the data reporting statement it is indicated that this is required as part of the journal format and should be added.

Reviewer #3

(Remarks to the Author)
Summary of key results
This is a controlled human influenza infection study involving 27 seronegative volunteers, aimed at identifying immune correlates of symptomatic versus asymptomatic disease. A range of immune parameters were profiled longitudinally in both blood and the nasal mucosa, including transcriptomics, soluble mediator analysis, flow cytometry, and ev vivo PBMC stimulation assays. The authors report that symptomatic infection is associated with earlier and more robust activation of innate pathways (notably interferon signaling and monocyte/DC activation), which in turn correlates with enhanced cellular responses. PBMC responsiveness prior to challenge also differed by eventual outcome, suggesting possible predisposing immune factors that determine clinical outcome.

Originality and significance
The dataset is very rich and the study design is rigorous, leveraging the unique advantages of a controlled infection model to capture very early immune events that are hard to study otherwise. As a result, this study provides unusually detailed temporal resolution, tracking multiple innate measurements as the infection course progresses. The idea that baseline responsiveness is a predictor of clinical trajectory is especially interesting. It’s a thoroughly interesting piece of work and I think many readers will find it thought-provoking.

Data and methodology

The transcriptional profiling, the daily sampling, symptom scoring, challenge modalities, and the statistical analyses are all rigorous and executed to an exceptionally high technical standard. I would note however that the basis of the post-infection analyses appears to rest on the assumption that pre-challenge immunity is comparable between symptomatic and asymptomatic individuals. This is especially critical for the nasal mucosa, which is the first point of contact between the host and the virus, but where 431 genes were differentially expressed pre-challenge between the outcome groups. This hints at the possibility of unmeasured confounding, potentially due to recent or existing, subclinical infections, or other mucosal factors. Such differences could potentially modify the post-challenge transcriptional/soluble mediator response and the clinical trajectory (symptomatic/asymptomatic).

As definitive exclusion of such confounding is impossible, it is essential to show parity in the expression of key pathway-defining genes (e.g., IFNs, ISGs, cytokines, chemokines etc etc) at baseline. Including baseline gene expression profiles representative of major innate/antiviral pathways (e.g., MX1, IFI27, IFITM3, etc.) alongside cluster-level summaries as presented in fig 3 would strengthen the paper.

Appropriate use of statistics and treatment of uncertainties
Use of DESeq2, IPA, and maSigPro is fully justified, and the longitudinal treatment of data is well executed. However, I noted that more than 6,500 genes were differentially expressed in nasal tissue at day 3. That’s >25% of the protein-coding transcriptome. This seems rather a lot for a mild, self-limiting, local infection. Is there precedent for this scale of transcriptional response from a nasal inoculation? Could some of this be technical?

Conclusions: robustness and validity
The conclusion that stronger early innate responses lead to both symptoms and better adaptive immunity is well supported, but this point could be further buttressed by demonstrating that baseline mucosal immunity was largely similar in all volunteers, irrespective of eventual clinical outcome. Also, plasma IFNα2a is only detectable in symptomatics, but ISGs are still upregulated in both groups, albeit lower in asymptomatics. Could this be due to MSD detection sensitivity? Or maybe unmeasured IFNs (like IFN-beta or IFN-lambda) are contributing. This discordance is interesting and could use a bit more discussion.

Suggested improvements
1. Please clarify how baseline neutralising antibody titres were measured. Were positive controls included? A detailed presentation of this data would help support the notion that there were no pre-existing differences in susceptibility pre-inoculation.
2. At day 3 post-inoculation, more than 6,500 genes (> 25% of the protein-coding transcriptome) were differentially expressed in nasal tissue. This is rather high for a mild, self-limiting, local infection. Can the authors provide an explanation or provide precedent for this magnitude of response following localised antigenic stimulation?
3. Possible confounding by other respiratory pathogens: Were participants screened for other respiratory viruses at baseline and during quarantine? Could subclinical viral priming (e.g., from common respiratory viruses) have differentially conditioned innate responsiveness?
4. The observation that type I IFN responses and ISG expression peak earlier in blood than in the nasal mucosa is quite interesting for a locally infecting respiratory virus. This inversion also appears in the soluble mediator data (fig 4). The paper notes this but does not explore potential mechanistic explanations (for example systemic spillover from early mucosal activation).
5. It would be useful for readers if the authors presented scatter or violin plots of canonical ISGs (e.g., MX1, IFI27, IFITM3) at all timepoints, including baseline. This would provide a more granular, intuitive sense of the temporal changes in these genes in both blood and nasal compartments.
6. The term nose is somewhat imprecise and carries connotations of anterior nares. The actual sampling site appears to be nasal scrapes (likely mid-turbinate or inferior turbinate). Please consider using “nasal mucosa” or specify the anatomical site more precisely throughout the text.
7. In plasma and mucosal lining fluid, IFNα2a is detectable only in symptomatics, yet ISGs are clearly upregulated in both groups (albeit to a lesser degree in asymptomatics). Could this reflect MSD detection sensitivity? Or might other type I IFNs (IFN-beta, IFN-lamda) be playing a role? A clearer discussion of this discordance would strengthen the argument.
8. I’m curious as to why we observe a biphasic postinfection soluble mediator response for IL-10 and IFN-g?
9. In the ex vivo PBMC stimulation experiments, only IL-1b exhibited a significantly higher plasma concentration in the supernatants of symptomatic individuals compared to asymptomatic individuals when stimulated with heat-inactivated virus (fig 7a). None of the other mediators showed a stastistically significant difference. I note that the Jackson symptom scoring system on line 558 does not include fever. Was there a difference in this symptom, considering the potent pyrogenic effects of this cytokine?
10. Can the authors speculate on why some individuals remained uninfected despite undetectable neutralising antibodies at baseline?

References
The paper is well referenced and appropriately credits prior work.

Clarity and context
The manuscript is clearly written overall, and the integration of multiple lines of evidence from transcriptomics, to plasma proteomics to ex vivo stimulation is done expertly and I commend the authors for this herculean effort.

Version 1:

Reviewer comments:

Reviewer #1

(Remarks to the Author)
In this revised manuscript, Papagyris and colleagues were highly responsive to prior critiques, with inclusion of new integrative analyses (Figure 7) and analysis of pre-existing T cell responses (Extended Data Figure 11), among other changes. A minor critique: Lines 336-341 of the revised integrative analyses needs to be re-written (at present it seems like a run-on sentence)? However, the new Figure 7 is excellent, and I appreciate the inclusion of additional authors (e.g. Davenport, Tsang) to assist with the analyses. The discussion and conclusion have been appropriately tempered as suggested by other reviewers.

Reviewer #2

(Remarks to the Author)
The authors addressed all my concerns. I have no further comments.

Reviewer #3

(Remarks to the Author)
Comments to the Authors

I would like to thank the authors for their thorough point-by-point responses to my comments. I appreciate the clarity and detail with which each concern was addressed.

In particular, I am grateful for the additional analyses and clarifications provided to strengthen the interpretation of early innate immune responses and their relationship to subsequent clinical and immunological outcomes. The expanded discussion and the clearer presentation of the relevant figures improve the manuscript and make the overall story easier to follow.

I also appreciate the revisions made to ensure consistent terminology when referring to symptom severity in the context of mild-to-moderate controlled human infection, as well as the strengthened limitations section. These changes appropriately temper interpretation and improve the balance of the discussion.

Overall, I believe the authors have addressed my comments satisfactorily and that the manuscript has been substantially improved as a result. I have no further major concerns.

Open Access This Peer Review File is licensed under a Creative Commons Attribution 4.0 International License, which permits use, sharing, adaptation, distribution and reproduction in any medium or format, as long as you give appropriate credit to the original author(s) and the source, provide a link to the Creative Commons license, and indicate if changes were

made.
In cases where reviewers are anonymous, credit should be given to 'Anonymous Referee' and the source.
The images or other third party material in this Peer Review File are included in the article’s Creative Commons license, unless indicated otherwise in a credit line to the material. If material is not included in the article’s Creative Commons license and your intended use is not permitted by statutory regulation or exceeds the permitted use, you will need to obtain permission directly from the copyright holder.
To view a copy of this license, visit https://creativecommons.org/licenses/by/4.0/

Response to Referees

In a revised manuscript, we consider it of priority to address concerns raised by reviewers about integrating analyses, performing additional follow up in T cells, and providing a clearer description of the transcriptomic analyses.

- As requested, integrative analyses, additional T cell experiments and clarifying descriptive text surrounding transcriptomics have been added in Figures: Fig. 7a-k, Fig. 8g, Extended Data Fig. 13a-i (integrative analyses), Extended Data Fig. 11a-h (additional T cell experiments) and Lines: 334-410, 479-480, 551, 911-970 (integrative analyses), 300-310, 896-909 (additional T cell experiments), 107-109, 113-121, 129, 138-141, 164-173, 825, 1096-1106 (clarifying descriptive text surrounding transcriptomics) and further detail has been provided to specific reviewers' comments below.

Importantly, we think this manuscript would be strengthened with a greater discussion about the clinical importance of these findings, as well as a greater discussion about the participants that remained uninfected.

- Further discussion regarding clinical importance (lines 536-546 and 570-575) and uninfected participants (lines 557-567) has been added.

As this was not registered as a trial, please report this as an observational study throughout the paper and present the results in a descriptive manner.

- The manuscript has been thoroughly reviewed with this in mind and amended. A statement has been added to the Methods (line 724-726) to state this was not a Clinical Trial. Since virus inoculation is an intervention, we have not specifically stated that the study is "observational" to avoid confusion but all results have been presented in a descriptive manner.

Please also provide a data availability statement. The Data Availability Statements must indicate any restrictions on data availability. Please indicate whether individual-level data will be made available, when, and for how long. Please provide a transparent path detailing how external requests will be evaluated, including contact information and an estimated timeframe for response when applicable.

- The Data Availability Statement has been added on Lines 1032-1047.

When revising your manuscript:

*** Please highlight all changes in the manuscript text file.**
*** Include a "Response to referees" document detailing, point-by-point, how you addressed each referee comment. If no action was taken to address a point, you must provide a compelling argument. This response will be sent back to the referees along with the revised manuscript.**
*** If you have not done so already please begin to revise your manuscript so that it conforms to our policy and format instructions here: https://www.nature.com/nm/submission-guidelines/aip-and-formatting Refer also to any guidelines provided in this letter.**
*** Include a revised version of any required reporting checklist. It will be available to referees (and, potentially, statisticians) to aid in their evaluation if the manuscript goes back for peer review. A revised checklist is essential for re-review of the paper.**
***Nature journals have recently announced an update to our guidance on reporting on sex and gender in research studies (see here). We strongly encourage researchers to follow the 'Sex and Gender Equity in Research – SAGER – guidelines' and to include sex and gender considerations for studies involving humans, vertebrate animals and cell lines where relevant to the topic of study (an overview can be found here). Authors should use the terms sex (biological attribute) and gender (shaped by social and cultural circumstances) carefully in order to avoid confusing both terms.**
***When preparing your revised manuscript, please be aware of our guidance on Sex and Gender reporting). Please note that:**
2b. For studies involving human research participants- The Reporting Summary should include whether sex and/or gender was considered in the study design and whether sex and/or gender of participants was determined based on self-report or assigned (and methodology used).
3. Data should be reported disaggregated for sex and gender where this information has been collected and consent has been obtained for reporting and sharing individual-level data; disaggregated numbers for individual experiments must be provided in the source data as appropriate whereas overall numbers may be provided in the Nature Portfolio Reporting Summary. Information on the 3 points above should be included in the revised manuscript and detailed in the cover letter.
***In addition, please note that if sex- and gender-based analyses have been performed a priori, results should be reported regardless of positive or negative outcome. We discourage conducting post hoc sex- and gender-based analysis if the study design is insufficient (for example, low sample size) to enable meaningful conclusions. If no sex- and gender-based analyses have been performed, please indicate the reasons for the lack of these analyses in the Reporting Summary.**

Reviewer #1 (Remarks to the Author):

Overall, I found the manuscript easy to read and to follow, with intriguing results implicating innate immune activation early following controlled human influenza infection to be associated with symptomatic disease.

The main limitation as acknowledged by the authors is the limited number of participants, particularly among the asymptomatic group (n=4). There isn't too much the authors can do about this limitation.

- We thank the reviewer for their detailed review of the manuscript and the supportive comments on the study findings overall.

What is missing in my opinion is an integrative analysis across modalities. The authors could consider MEFISTO (PMID 35027765) or Bayesian approaches (PMID 38146838).

- We are grateful to the reviewer for suggesting this and have taken the opportunity to explore a number of mathematical modelling and machine learning approaches to identify the relationships between immune markers and their underlying putative regulatory structures. Results from unsupervised multi-modal MEFISTO analysis revealed 4 factors with distinct temporal kinetics that define the primarily interferon-driven response in both blood and nose; the inflammatory mediator responses restricted to the nasal compartment; the later adaptive immune response; and pro-inflammatory cytokine-related genes in the blood that distinguish symptomatic from asymptomatic individuals. Using conditional dependency network analysis, we analysed the top-weighted features that contribute to these factors and thus identified the relationships between the most explanatory variables. This is shown in Figure 7f-g and described on lines 918-934.

By integrating the multiple related datasets, this might provide additional insight into relationships between genes/proteins/cells beyond individual factor analysis. For example, what might be driving differential IL-10 protein levels? Could such an approach help explain the contradictory results between IL-10 measured in the nasal fluid and IL-10 measured in plasma?

- While informative, the large volume of gene expression data included in the MEFISTO analysis appeared to swamp the viral load and cellular immune measures, which did not appear amongst the top-weighted factors. To better address the reviewer's comment regarding IL-10, we undertook further analysis using vector autoregression (VAR), a framework for multivariate time-series modelling, without the inclusion of the

RNAseq dataset. This enabled the characterisation of directional associations among immune markers with directionality inferred from prediction across successive timepoints. This indicated IFN-γ to be strongly driven by viral load and was in turn strongly associated with IL-10 levels in blood, explaining the early peak. We hypothesise that the later peak is due to the coincidental peak of activated and proliferating T cells. This is shown in Fig. 7h-i and described on lines 360-377.

- Pairwise correlations between blood and nasal IL-10 levels across timepoints (shown below) were weak and largely non-significant, suggesting distinct compartmentalization of these responses. This partly explains why the same relationships were not seen in integrative analysis of nasal mucosa data.

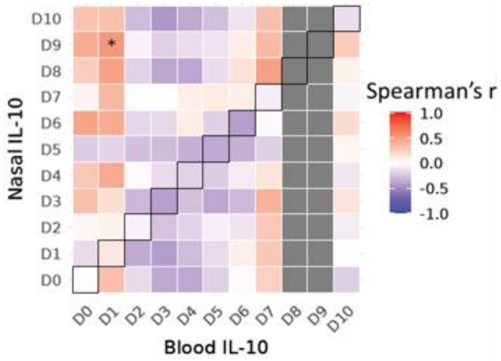

Figure 1: Heatmap showing pairwise correlations between blood and nasal IL-10 levels across timepoints (*$P < 0.05$).

Could this help explain why Day 14 responses to stimulation were lower than pre-infection responses? I think this would significantly improve the manuscript and interpretability for readers.

- Pairwise correlation analysis between *in vivo* soluble mediator concentrations during the infection and *in vitro* day 14 PBMC mediator secretion indeed revealed negative correlations between day 3 *in vivo* cytokines and chemokines and day 14 *in vitro* levels (mainly IFN-γ). This provides an explanatory link between the magnitude of the early systemic innate activation after infection and the refractoriness in mediator secretion upon secondary viral stimulation. This has now been added in the manuscript (Fig. 8g and lines 444-451).

In addition, although the authors analyzed comparisons between symptomatic and asymptomatic individuals, did the authors look to see whether immune responses influenced other outcomes? Rates of viral clearance? Post-infection levels of neutralizing antibodies?

- Piecewise linear regression modelling has now been undertaken to see whether immune responses influenced virological outcomes, including dynamic readouts such as viral growth rate and clearance. This showed earlier nasal IFN-γ activation time to be correlated with lower VL growth rate and peak size (r = -0.6, $P = 0.003$ and r = -0.63, $P = 0.005$, respectively), implying an association between IFN-γ production and restraint of viral replication. In addition, higher VL growth rate correlated with higher peak symptom score (r = 0.55 and $P = 0.008$). This is now described on lines 336-341.
- We thank the reviewer for suggesting further comparing acute immune responses with antibody production. This is being incorporated into a separate manuscript, where we will have more space to include the comprehensive and detailed antibody analyses that would not be possible here. We hope this is acceptable.

Line 206-208/Figure 5a/Supplementary Figure 6: Was the flow cytometry performed with overlapping panels? If so would share those panels and better describe in the methods how this was performed.

- Flow cytometry was undertaken using three separate panels for the analysis of the phenotype and activation status of (1) monocytes and dendritic cells, (2) NK cells and (3) T cells. These are detailed in Extended Data Table 7 with markers and antibodies used in the panels named "Innate cell panel", "NK cell panel" and "T cell panel", respectively. These did not sufficiently overlap to enable population clustering with combined data from all three panels. A 4th panel was used to analyse cellular responses after *in vitro* PBMC activation and is also detailed in Extended Data Table 7, named "ICS for PBMC Stimulation". Additional detail has now been added to the Methods (lines 859-890).

I find it curious that the authors only show the UMAP data for monocytes/DCs; would be interested to see data for different cell subsets as well.

- We apologise for omitting these UMAP plots from the T cell and NK cell figures. On further consideration, we have inserted revised UMAP data for myeloid cells, NK cells and T cells to show grouped participants rather than a single representative individual in Fig. 5a, 6a and 6f. We believe these plots now provide a more comprehensive overview of the changes in cellular composition over the course of infection.

And, did the authors perform any clustering of cell phenotype data using unsupervised approaches?

- As suggested, unsupervised approaches to clustering of flow phenotype data were explored. However, while analysis of myeloid cell data using FlowSOM clustering on UMAP mapping confirmed the relationships between major population clusters, they did not adequately delineate the monocyte subpopulations of interest. This is likely due to the transitory and phenotypically less distinct nature of monocyte subpopulations meaning that definitions of monocytes as they differentiate from classical to intermediate and non-classical are not clear-cut. In light of this, we undertook both FlowSOM clustering (Extended data Fig. 7b) and manually gated subpopulations for this particular plot (Fig. 5a), explaining why we have done so (lines 233-235). This provides a comprehensive overview of changes in these subpopulations over the course of infection, with quantitative data and summary statistics shown later in Fig. 5, Extended Data Fig. 7c and Extended Data Fig. 8,9.
- FlowSOM clustering on UMAP analysis of NK and T cell has now been added in Fig. 6a,b (NK cells) and f,g (T cells) and in Extended Data Fig. 10b,c (NK cells) and Extended Data Fig. 12b,c (T cells). Further analysis of cluster percentage changes over the course of infection has also been added in Fig. 6c,h and Extended Data Fig. 10c (NK cells) and Extended Data Fig. 12c (T cells) and described on lines 280-287 (NK cells) and on lines 312-317 (T cells).

Minor: Line 106: In contrast, following infection…. Suggest changing following infection to "among infected individuals"

- This has been updated (lines 127-128)

Reviewer #2 (Remarks to the Author):

This is study is elegant, sound and of a great importance for defining the link between the early innate immunity and symptomatic influenza virus infection as well as differential immunity in asymptomatic versus symptomatic individuals.

- We thank the reviewer for their careful assessment of the manuscript and positive comments.

Data and methodology

-The definition of symptomatic infection was "…total adjusted Jackson symptom score of 6 or more over the first 6 days post-inoculation and reporting having had a cold/flu or reporting nasal discharge on 3 or more consecutive post-inoculation days". The Jackson score is a validated tool that encompasses all the symptoms usually associated with having a "cold/flu" including nasal discharge – so it is unclear why the additional definition was needed. Can the authors justify the use of this composite definition for symptomatic infection?

- This definition was based on the Jackson score as originally published (https://jamanetwork.com/journals/jamainternalmedicine/article-abstract/561967), the only modification being a reduced scoring threshold for increased stringency as described in Gwaltney *et al*. J Infect Dis (1980) (https://academic.oup.com/jid/article/142/6/811/934840?login=true). Sensitivity analyses were performed for the composite vs score-only endpoints and there was no difference. The modified Jackson's score has a lower threshold for defining symptomatic infection (a score of 6 or more over the first 6 days post-inoculation) compared with the original Jackson score (score of 14 or more) and sensitivity analyses showed that the modified scoring system showed greater discrimination of symptomatics vs asymptomatics by multiple measures.

Interpretation

-One of the key conclusions made by the authors is that pre-existing innate immune responsiveness was associated with the subsequent development of symptoms. Individuals were selected for inclusion on the basis of an absence of neutralising antibodies against the infecting strain, but there was no assessment of prior T cell responses. Cross-reactive T cell responses have been associated with protection against symptoms in infection with both seasonal and pandemic influenza strains (e.g. Sridhar et al 2013 Nat Med DOI: 10.1038/nm.3350, Wilkinson et al 2012 Nat Med DOI:

10.1038/nm.2612, Hayward et al 2015 DOI: 10.1164/rccm.201411-1988OC). An assessment of influenza-specific T cell responses prior to infection is needed and should therefore be considered as part of this work.

- We thank the reviewer for highlighting the need to rule out pre-existing T cells and any correlation with symptoms as a confounder. To address this, we used IFN-γ ELISpot to quantify influenza-specific T cells in PBMCs from peri-inoculation timepoints. Due to shortage of pre-inoculation PBMCs, day 1 and 2 samples were used for some individuals but based on extensive data from the literature and in our hands, we are confident that no T cell proliferation had yet occurred at these timepoints and they are therefore sufficiently analogous to pre-inoculation samples for the analysis of antigen-specific T cell frequencies. These were stimulated with two influenza peptide megapools developed by Alessandro Sette's group (La Jolla Institute of Immunology) (https://currentprotocols.onlinelibrary.wiley.com/doi/10.1002/cpz1.934) comprising $CD4^+$ and $CD8^+$ T cell-biased epitopes, made up of a combination of previously experimentally defined epitopes and overlapping peptides. No significant differences were seen between infected and uninfected or symptomatic and asymptomatic individuals, nor any significant correlation between frequency of T cells and viral load or symptoms. Thus, there was no evidence of T cells having significantly contributed to differences between symptomatic and asymptomatic infection in this group of participants. This is now described in Extended Data Fig. 11 and lines 300-310, 516-519 and 896-909.

The authors describe their immunological findings as "correlates of severity" (e.g. line 241) and alternate using the terms "disease severity" and "symptom severity" to describe findings. This study involves young people with mild influenza infection (no hospitalisation or oxygen requirements) and these immunological findings may not be the same pathways involved in severe disease. The authors should be consistent about describing their results as reflecting symptom severity in mild disease. Furthermore, in the limitation of the study section, it would be important to mention that the early innate immune response might differ in patients with life-threatening influenza outcomes.

- We have now revised the wording to make clear that these findings are in the context of mild-moderate disease only (lines 37, 72, 74, 269-270, 463, 485) and have highlighted in the limitations section that these differences may differ in patients with severe infection (lines 553-556).

The conclusion of the manuscript does not reflect the content of the work well. The manuscript describes a nasal inoculation challenge model, but the conclusion focuses around vaccine responses / reactogenicity – it does not necessarily follow that these would be the same. Consider rephrasing this conclusion to reflect the key findings.

- We thank the reviewer for flagging this. We have revised the Discussion to avoid over-extrapolation (lines xxx).

Analytical approach

The longitudinal approaches to analyses of all immune parameters measured, in particular the transcriptomic data presented posed a challenge. The authors describe a number of comparisons in the text (nasal versus blood, infected versus uninfected, symptomatic versus asymptomatic). Overall, the presentation and interpretation of the transcriptomic data is not easy to navigate and should be improved to maximise clarity. In particular:
-In Figure 2b and Figure 2d the axes have been condensed so that the gene names are distorted and too small to read. These should either be removed (if the overall goal is to see the clusters only) or expanded to be legible.

- Thank you for pointing this out. The gene names in Figure 2b and Figure 2d have now been removed from the main figure for clarity, and a supplementary figure (Supplementary Fig. 1) has been added showing a high-definition version of the panels, allowing the reader to zoom in and scrutinise the gene names. The genes are also listed in a supplementary table (Supplementary Table 2), which is signposted in the figure legend and on lines 1105-1106. The other panels have also been reviewed for improved clarity.

The data in Figures 2f, 2g, 2h, 2i and 2j show differentially expressed genes in symptomatics and asymptomatics at all time points based on selected pathways from pathway analysis (shown in Figure 2c). By selecting genes for inclusion in each plot based on pathway analysis, there is overlap of the genes shown such that several genes are represented in multiple of plots 2f-j (e.g. SOCS3, IRF7, HLA-B-C, C5) as well as in Figure 2b. Figures 2f-j add little value over 2b and 2c, and make the overall interpretation of the data in Figure 2 difficult. Consider removing these.

- As suggested, the previous figures 2f, 2g, 2h, 2i and 2j have been removed from Fig. 2 and transferred to Extended Data Fig. 3.

The authors should consider formally showing a two-way comparison of the most relevant data to the narrative. Much of the narrative is around differences between gene expression in blood between symptomatics and asymptomatics but this comparison is not formally shown (with all differentially induced genes shown relative to baseline). Consider including this comparison for a timepoint of interest (for example a volcano plot of differentially expressed genes between asymptomatics and symptomatics at Day 2 or Day 3).

- We thank the reviewer for the suggestion and agree that this can be an additionally informative analysis method with some datasets. Here, comparisons of symptomatic and asymptomatic groups were undertaken but found to be substantially less revealing than the analyses shown in the main figures. There are two main reasons for this. First, the asymptomatic group is small, a recognized limitation that we emphasise in the discussion, and therefore power to detect differences is limited. Second, there is substantial inter-individual variation in translational studies such as this, with diverse genetics and antigenic exposure history. By defining differentially expressed genes relative to each individual's baseline and taking advantage of the entirety of longitudinal data, we are able to better control for these factors, thus revealing more of the changes related to infection. This is further explained in lines 109-118.
- As suggested, a comparison between symptomatic and asymptomatic participants in the blood and nasal mucosa at day 3 has now been added in Extended Data Fig. 2g and h, respectively, and described in lines 118-121.

The transcriptomic data have been analysed using maSigPro to identify temporal trends over time, resulting in the identification of gene clusters. In the description of the analysis this describes that this identifies differentially expressed genes "between groups and over time". Are the differentially expressed genes shown in Figure 3 relative to baseline (i.e. each timepoint relative to pre-inoculation sample) or between the two groups?

- maSigPro models each gene's expression as a polynomial regression over time with group-specific effects, then uses a second regression step and clustering to identify

and summarize genes whose temporal trajectories differ between each group. Thus, it uses the dataset in its entirety to generate the model and determine statistically significant differences in gene expression pattern between groups rather than either defining DEGs relative to baseline or by comparing between groups at each separate timepoint. This is therefore a third approach that enhances statistical power to define true positives and supports our conclusions by the consistency of its findings.

In describing the results in Figure 3a-b the authors compare the lines between symptomatics and asymptomatics (e.g. line 143 states "lower but more prolonged response for asymptomatics"). It does not appear that the conclusion that the response in Figure 3a is more prolonged in asymptomatics is valid based on data presented. Do these trajectories have a statistical test to indicate they are different between groups?

- On further review, we agree that the statement of a "more prolonged response for asymptomatics" is not sufficiently supported by the data. This has now been removed from the manuscript (ex-lines 143 and 270).

The term PBMC is used in the abstract without definition – an alternative term or definition should be included here.

- The definition of PBMC has been added in the abstract.

Similarly, in first use in the manuscript (line 283) the term PBMCs is not defined, but it is later defined in the methods – this definition should be moved to the first use.

- The definition of PBMC has now been added to the first use (line 303).

The manuscript is lacking key demographic details on participants in each group (sex, age) which are important for generalisability and interpretation of findings. These should be included for each group analysed. Some of this information (sex only) is contained in the reporting summary but should be moved to the manuscript.

- Demographic characteristics have now been added in Extended Data Table 1.

In the ethics statement the term "ICH" should be defined.

- The definition of ICH (International Council for Harmonisation) has now been added (line 721).

In description of flow cytometry data in line 270 the authors describe “flatter but more prolonged patterns of T cell activation”. This appears to relate to Extended Data Fig 9e where there is no difference between these groups at later time points and it does not appear that it is valid to suggest the response is more prolonged.

- We agree and the expression “more prolonged” has now been removed (ex-lines 143 and 270).

In the results presenting data from flow cytometry experiments there is variation in the number of samples presented (e.g. Figure 5 and 6 describe sample numbers “up to 18” or “up to 4”) but it is not described in the Methods why the sample number varies. What were the inclusion / exclusion criteria for a sample to be part of the results presented?

- The exact numbers of samples included are now detailed in the Methods section and explanation provided for missing samples/datapoints (lines 870-881).

For the cytokine stimulation data in Figure 7c the authors comment that the number of individuals included (14) did not allow for comparison between symptomatic and asymptomatic patients (line 294-295). Subsequently Figure 7e compares symptomatic and asymptomatic individuals with either 14 (or fewer) samples included at each timepoint. How were decisions made regarding data inclusion here?

- Regarding now Fig. 8c (previously Fig. 7c), comparative statistics were not done between symptomatic and asymptomatic participants as only two asymptomatics could be assessed due to limited availability of cells. In now Fig. 8e (previously Fig. 7e), samples were available for 3 asymptomatic participants and a two-way mixed-effects model was used. To simplify the analysis and make explicit the number of participants in each comparison, we have replaced the mixed-effects model with Mann-Whitney test for day 0 (symptomatics, n = 11 and asymptomatics, n = 3) and day 14 p.i. (symptomatics, n = 10 and asymptomatics, n = 3). Fig. 8 (previously Fig. 7) has been updated and text clarified on lines 1213-1214 and 1220-1221.

In the Methods the quantification of influenza RNA based on M gene presence is described (lines 572-574) but the primer sequences are not provided. Please include further detail on how this qPCR was performed.

- Primer sequence information is now included in the Methods and further detail on the qPCR provided by citation of a previous study using the same method (lines 768-770).

In the representative gating (Extended data Figure 6) there are no numbers (percentages) on the gates shown; in the data reporting statement it is indicated that this is required as part of the journal format and should be added.

- We apologise for this omission and percentages have now been added to the representative plots in now Extended data Fig. 7a, Extended data Fig. 10a, Extended data Fig. 12a and Extended data Fig. 14b.

Reviewer #3 (Remarks to the Author):

Summary of key results
This is a controlled human influenza infection study involving 27 seronegative volunteers, aimed at identifying immune correlates of symptomatic versus asymptomatic disease. A range of immune parameters were profiled longitudinally in both blood and the nasal mucosa, including transcriptomics, soluble mediator analysis, flow cytometry, and ev vivo PBMC stimulation assays. The authors report that symptomatic infection is associated with earlier and more robust activation of innate pathways (notably interferon signaling and monocyte/DC activation), which in turn correlates with enhanced cellular responses. PBMC responsiveness prior to challenge also differed by eventual outcome, suggesting possible predisposing immune factors that determine clinical outcome.

Originality and significance
The dataset is very rich and the study design is rigorous, leveraging the unique advantages of a controlled infection model to capture very early immune events that are hard to study otherwise. As a result, this study provides unusually detailed temporal resolution, tracking multiple innate measurements as the infection course progresses. The idea that baseline responsiveness is a predictor of clinical trajectory is especially interesting. It's a thoroughly interesting piece of work and I think many readers will find it thought-provoking.

- We thank the reviewer for their detailed assessment of the manuscript and the supportive comments.

Data and methodology
The transcriptional profiling, the daily sampling, symptom scoring, challenge modalities, and the statistical analyses are all rigorous and executed to an exceptionally high technical standard. I would note however that the basis of the post-infection analyses appears to rest on the assumption that pre-challenge immunity is comparable between symptomatic and asymptomatic individuals. This is especially critical for the nasal mucosa, which is the first point of contact between the host and the virus, but where 431 genes were differentially expressed pre-challenge between the outcome groups. This hints at the possibility of unmeasured confounding, potentially due to recent or existing, subclinical infections, or other mucosal factors. Such differences could potentially modify the post-challenge transcriptional/soluble mediator response and the clinical trajectory (symptomatic/asymptomatic).

As definitive exclusion of such confounding is impossible, it is essential to show parity in the expression of key pathway-defining genes (e.g., IFNs, ISGs, cytokines, chemokines etc etc) at baseline. Including baseline gene expression profiles representative of major innate/antiviral pathways (e.g., MX1, IFI27, IFITM3, etc.) alongside cluster-level summaries as presented in fig 3 would strengthen the paper.

- We thank the reviewer for this suggestion. A detailed list of the 431 DEGs pre-challenge between symptomatic and asymptomatic participants is now provided as an Excel file (Supplementary Table 1). On detailed review, while the list contains some immune-related genes (shown below in Table 1), no IFNs, ISGs or cytokines/chemokines associated with viral infections are present. The top 10 DEGs are now shown in Extended Data Table 2 to show that there are no immune-related genes amongst the most differentially expressed genes. Furthermore, IPA analysis of all 431 genes showed no enrichment for any immune-related genes except for non-canonical NF-κB signalling (Extended Data Fig. 2e). This is further explained on lines 114-118 and 1250-1251.

Table 1. Immune-related genes among the 431 DEGs appeared between symptomatic and asymptomatic in the nasal mucosa pre-inoculation.

<u>Innate immunity / inflammation:</u>
MASP2 – Lectin complement pathway (innate immunity)
PGLYRP3 – Pattern-recognition receptor (bacterial sensing)
RNASE7 – Antimicrobial peptide (epithelial innate defense)
HP (Haptoglobin) – Acute-phase protein
CST3 (Cystatin C) – Immune regulation, antigen presentation
HMGN1 – Alarmin / danger-associated molecular pattern (DAMP)
NINJ1 – Innate immune signaling, cell death regulation
GSDMA – Inflammatory cell death (pyroptosis-related)
A2ML1 – Protease inhibition, inflammatory response
SERPINB11 – Serine protease inhibitor (immune-associated)
SCGB1A1 – Immune-modulatory epithelial secreted protein
<u>Immune signaling / regulation:</u>
TRAF5 – TNF receptor / NF-κB signaling
MAPK3 (ERK1) – Immune signal transduction
IL1RN – Interleukin-1 receptor antagonist
ILF2 – Immune gene transcription regulation
LAMTOR1 – mTOR signaling in immune cells
SKAP1 – T-cell receptor signaling
LMO2 – Hematopoiesis and immune cell development
ACVRL1 – TGF-β signaling, immune-vascular interface
SDC1 (CD138) – Plasma cells, immune cell marker
ANXA2 – Inflammation and immune cell migration

ABHD12 – Immune regulation, microglial function
NK / immune surveillance
ULBP2 – NKG2D ligand (NK-cell activation)
NKTR – Natural killer cell-associated protein
<u>Immune-associated ubiquitin / transcription factors:</u>
TRIM52
TRIM66
RNF213-AS1 (linked to immune vascular disease)
RNF167

- At the reviewer's suggestion, key genes involved in major innate/antiviral pathways MX1, IFI27, IFITM3 (or others like IFI6, ISG15, IFI44, RSAD2, IFIT1, IFIT3, OAS3, CCL2, USP18) that had been found significantly differentially expressed post-infection were systematically compared between symptomatic and asymptomatics and now shown in Extended Data Fig. 5. In all cases, expression levels at baseline showed no significant correlation with symptom scores after infection as shown below:

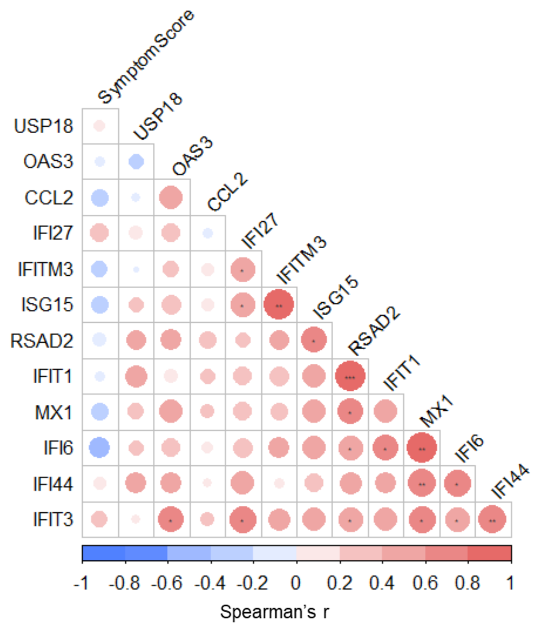

Figure 2. Spearman's correlations between baseline gene counts from ISGs in the nasal mucosa and modified Jackson's symptom score.

- Thus, together with data from soluble mediators and immune cell phenotyping, no significant differences were identified between symptomatic and asymptomatic participants in pathway-defining genes, proteins or immune cell populations that might imply a biologically-relevant signature at baseline associated with differential outcome.

Appropriate use of statistics and treatment of uncertainties
Use of DESeq2, IPA, and maSigPro is fully justified, and the longitudinal treatment of data is well executed. However, I noted that more than 6,500 genes were differentially expressed in nasal tissue at day 3. That's >25% of the protein-coding transcriptome. This seems rather a lot for a mild, self-limiting, local infection. Is there precedent for this scale of transcriptional response from a nasal inoculation? Could some of this be technical?

- We agree that >6500 DEGs in nasal tissue is a large number but note that not all of these are protein-coding genes. While we filtered out low-expressed transcripts and ribosomal RNA before differential expression analysis, non-protein coding genes including lncRNA and miRNAs remain. Studies of PBMC stimulated *in vitro* with influenza virus have previously shown 2,906 DEGs at 6 hours, with 3,888 DEGs in purified monocytes (https://www.frontiersin.org/journals/immunology/articles/10.3389/fimmu.2023.1168784/full). Meanwhile, infection of the Calu-3 airway epithelial cell line with SARS-CoV-2 resulted in 2473 upregulated and 3611 downregulated genes at 24 hours post-infection (https://pmc.ncbi.nlm.nih.gov/articles/PMC7723856/), so the numbers of DEGs are similar to these models.
- There are no equivalent studies of naturally-infected patients as pre-infection timepoints are rarely available to permit longitudinal analysis. Previous influenza CHIM studies have focused on whole blood transcriptomics and primarily used microarrays so cannot be directly compared. Unpublished data from our group in healthy volunteers challenged with RSV showed 1236 DEGs in nasal tissue at day 7 compared to baseline (shown below). However, the RNAseq platform and analysis pipeline differ between the two studies and RSV is known to be less virulent with symptoms less marked compared to influenza challenge. Therefore, in summary, thousands of genes can be differentially expressed following respiratory virus infection of mucosal cells and the literature does not contradict our findings, which we are confident are technically robust.

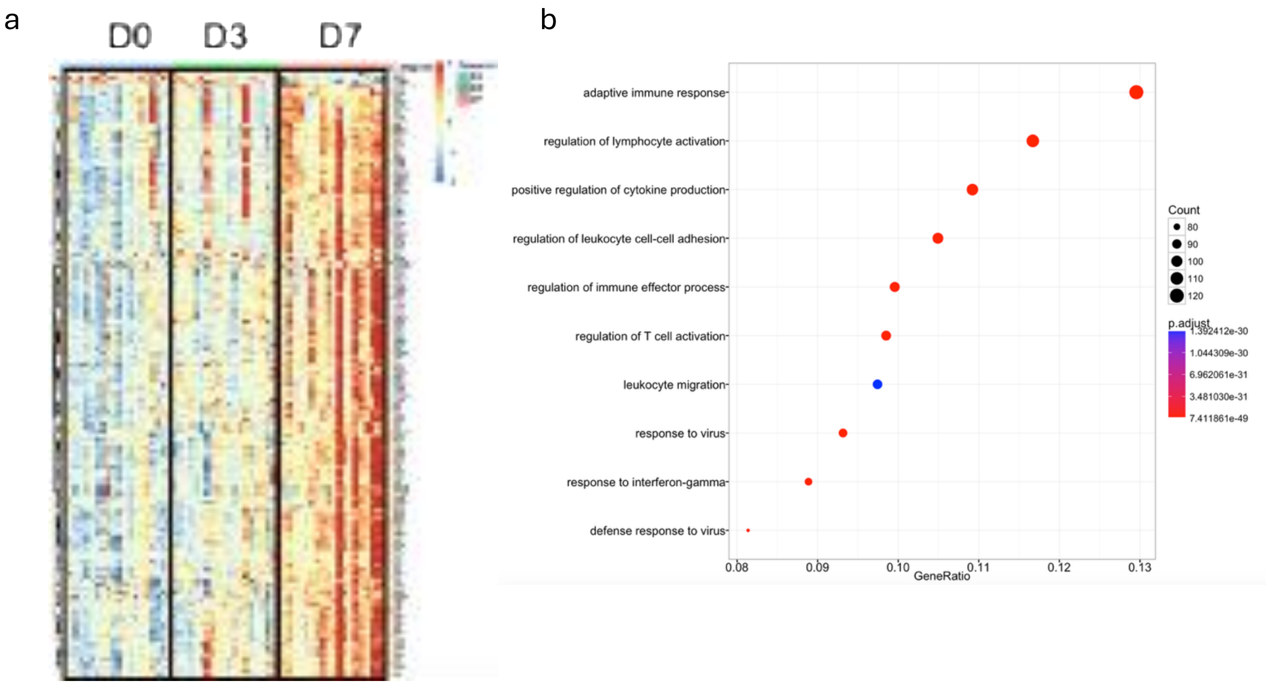

Figure 3. (a) Heatmap representation of DEGs at day 7 compared to baseline in nasal tissue of healthy volunteers challenged with RSV. (b) DEG-enriched pathways (DEGs from Figure 5a) as shown by Gene Ontology (GO) analysis.

Conclusions: robustness and validity

The conclusion that stronger early innate responses lead to both symptoms and better adaptive immunity is well supported, but this point could be further buttressed by demonstrating that baseline mucosal immunity was largely similar in all volunteers, irrespective of eventual clinical outcome. Also, plasma IFNα2a is only detectable in symptomatics, but ISGs are still upregulated in both groups, albeit lower in asymptomatics. Could this be due to MSD detection sensitivity? Or maybe unmeasured IFNs (like IFN-beta or IFN-lambda) are contributing. This discordance is interesting and could use a bit more discussion.

- As suggested, we have now further clarified the point based on transcriptomics, soluble mediator and (blood only) flow cytometry, ELISpot and serum microneutralisation assay that all volunteers had similar immune profiles prior to virus inoculation (lines 557-559). As implied by the reviewer, we cannot categorically rule out baseline differences using the assays available as indeed there are differences in sensitivity depending on assay and analyte. Indeed, plasma and nasal IFN-α2a levels measured by MSD were often below the detection level, mostly in the asymptomatics.

- IFN-β and IFN-λ1 were indeed measured using MSD. IFN-β, shown in Extended Data Fig. 6c and d, was significantly higher in symptomatic individuals at pre-inoculation and at day 2 and day 3 post-inoculation. Regarding IFN-λ1, shown in Fig. 5a,b, and Extended Data Fig.6a,b, there was a non-significant trend for higher responses post-infection for symptomatic participants in both plasma and nasosorption samples. However, plasma and nasosorption levels of all three interferons were frequently undetectable depending on timepoints and individuals, limiting interpretation. This is now discussed in the limitations section on lines 560-563.

Suggested improvements
1. Please clarify how baseline neutralising antibody titres were measured. Were positive controls included? A detailed presentation of this data would help support the notion that there were no pre-existing differences in susceptibility pre-inoculation.

- Baseline neutralising antibody titres were measured by serum microneutralisation assay. A description of the assay has been added in the Methods section (lines 772-785). An in-house serum standard of known high titre was used as positive control across the whole study. Mean titres of all participants and groups based on infection status and symptomatology are now shown in Extended Data Table 1.

2. At day 3 post-inoculation, more than 6,500 genes (> 25% of the protein-coding transcriptome) were differentially expressed in nasal tissue. This is rather high for a mild, self-limiting, local infection. Can the authors provide an explanation or provide precedent for this magnitude of response following localised antigenic stimulation?

- This has been addressed above.

3. Possible confounding by other respiratory pathogens: Were participants screened for other respiratory viruses at baseline and during quarantine? Could subclinical viral priming (e.g., from common respiratory viruses) have differentially conditioned innate responsiveness?

- Nasal wash collected from participants the day before inoculation and at day 4 post-inoculation was tested by multiplex PCR for common respiratory viruses and bacteria (Influenza A and B Viruses, Respiratory Syncytial Virus, Rhinovirus/Enterovirus, Parainfluenza 1-4 Viruses, Adenovirus, Human Metapneumovirus, Bordetella pertussis, Bordetella holmesii, a subset of Bordetella bronchiseptica and Mycoplasma pneumoniae) in an accredited diagnostic lab. A negative PCR test at day -1 was required

for inclusion in the study (a positive - other than influenza - PCR test during quarantine meant exclusion from analysis [shown in Fig. 1a]). This has now been added to Methods lines 738-744.

4. The observation that type I IFN responses and ISG expression peak earlier in blood than in the nasal mucosa is quite interesting for a locally infecting respiratory virus. This inversion also appears in the soluble mediator data (fig 4). The paper notes this but does not explore potential mechanistic explanations (for example systemic spillover from early mucosal activation).

- In a previous study of SARS-CoV-2 controlled human infection conducted by our group, this difference was also noted. Taking advantage of single-cell RNAseq data in that study, we showed that type I IFN was produced mainly by monocytes. While monocytes are present in the nasal mucosa, they are much more plentiful in the circulation and we therefore speculated that the IFN signal triggered by viral infection reached detectability more quickly in blood for this reason, although a number of other hypotheses exist. We now include a short section about this in the Discussion (lines 468-473) and cite the SARS-CoV-2 paper for more detail.

5. It would be useful for readers if the authors presented scatter or violin plots of canonical ISGs (e.g., MX1, IFI27, IFITM3) at all timepoints, including baseline. This would provide a more granular, intuitive sense of the temporal changes in these genes in both blood and nasal compartments.

- The manuscript has been updated to include scatter plots showing individual longitudinal expression counts of the same canonical ISGs discussed in a previous comment (MX1, IFI27, IFITM3, IFI6, ISG15, IFI44, RSAD2, IFIT1, IFIT3, OAS3, CCL2, USP18) (lines 165-169 and 171-173). The plots are now shown in Extended Data Fig. 5a,b.

6. The term nose is somewhat imprecise and carries connotations of anterior nares. The actual sampling site appears to be nasal scrapes (likely mid-turbinate or inferior turbinate). Please consider using "nasal mucosa" or specify the anatomical site more precisely throughout the text.

- As suggested, we have amended the text to use "nasal mucosa" instead of "nose" where relevant.

7. In plasma and mucosal lining fluid, IFNα2a is detectable only in symptomatics, yet ISGs are clearly upregulated in both groups (albeit to a lesser degree in asymptomatics). Could this reflect MSD detection sensitivity? Or might other type I IFNs (IFN-beta, IFN-lamda) be playing a role? A clearer discussion of this discordance would strengthen the argument.

- We address this in a response to a similar comment above

8. I'm curious as to why we observe a biphasic postinfection soluble mediator response for IL-10 and IFN-g?

- This is indeed an interesting observation, with both IFN-γ and IL-10 showing two peaks at day 3 and day 6 post-infection in blood. We hypothesise that this reflects the innate and adaptive phases of the antiviral response, with IFN-γ initially driven by viral load and later produced at high levels by activated T cells. The newly-added machine learning analysis partly supports this by showing strong associations between VL and IFN-γ. Interestingly, IFN-γ is then shown to promote IL-10 expression, potentially explaining their similar patterns of expression. We now discuss this on lines 201-203, 366-369, 375-377 and 400-402.

9. In the ex vivo PBMC stimulation experiments, only IL-1b exhibited a significantly higher plasma concentration in the supernatants of symptomatic individuals compared to asymptomatic individuals when stimulated with heat-inactivated virus (fig 7a). None of the other mediators showed a stastistically significant difference. I note that the Jackson symptom scoring system on line 558 does not include fever. Was there a difference in this symptom, considering the potent pyrogenic effects of this cytokine?

- Fever (> 37.7 °C) occurred in only two participants, at one and three timepoints respectively. Fever is seldom a feature of the mild-moderate infections that occur following human influenza challenge. It therefore has limited discriminatory value in this setting and we were unable to examine the relationship between IL-1β and fever.

10. Can the authors speculate on why some individuals remained uninfected despite undetectable neutralising antibodies at baseline?

- In the context of low levels of strain-specific antibodies, protection is likely multi-factorial. Antibodies below the limit of detection or unmeasured (such as mucosal IgA

or non-neutralising antibodies) will differ between individuals, T cells in the nasal mucosa may play a role in immediate clearance of inoculum and intrinsic and innate immunity are likely to modulate risk of infection. These factors have been explored in the context of influenza, RSV and SARS-CoV-2 in a series of publications from our group and others. We have now added a section discussing this (lines 563-567) and cited those papers.

References
The paper is well referenced and appropriately credits prior work.

Clarity and context
The manuscript is clearly written overall, and the integration of multiple lines of evidence from transcriptomics, to plasma proteomics to ex vivo stimulation is done expertly and I commend the authors for this herculean effort.

- We thank the reviewer for their positive comments.
